# Proteomic and Physiological Analysis of the Response of Oat (*Avena sativa*) Seeds to Heat Stress under Different Moisture Conditions

**DOI:** 10.3389/fpls.2016.00896

**Published:** 2016-06-22

**Authors:** Lingling Chen, Quanzhu Chen, Lingqi Kong, Fangshan Xia, Huifang Yan, Yanqiao Zhu, Peisheng Mao

**Affiliations:** ^1^Beijing Key Laboratory of Grassland Science, Forage Seed Lab, China Agricultural UniversityBeijing, China; ^2^Chifeng Academy of Agricultural and Animal SciencesChifeng, China; ^3^Chengdu Municipal Development and Reform CommissionChengdu, China; ^4^Institute of Grassland Research of Chinese Academy of Agricultural ScienceHohhot, China

**Keywords:** oat seed, heat stress, moisture content, seed deterioration, proteomic, ROS

## Abstract

Seeds lose their viability when they are exposed to high temperature and moisture content (MC) during storage. The expression and metabolism of proteins plays a critical role in seed resistance to heat stress. However, the proteome response to heat stress in oat (*Avena sativa*) seeds during storage has not been revealed. To understand mechanisms of heat stress acclimation and tolerance in oat seeds, an integrated physiological and comparative proteomic analysis was performed on oat seeds with different MC during heat stress. Oat seeds with 10% and 16% MC were subjected to high temperatures (35, 45, and 50°C) for 24 and 2 days, respectively, and changes in physiological and biochemical characteristics were analyzed. The results showed that seed vigor decreased significantly with temperature increase from 35 to 50°C. Also, the proline content in 10% MC seeds decreased significantly (*p* < 0.05) whereas that in 16% MC seeds increased significantly (*p* < 0.05) during heat treatment from 35 to 50°C. There were no significant differences in malondialdehyde content in 10% MC seeds with temperature from 35 to 50°C, but a significant (*p* < 0.05) decline occurred in 16% MC seeds at 45°C. Proteome analysis revealed 21 significantly different proteins, including 19 down-regulated and two up-regulated proteins. The down-regulated proteins, notably six heat shock proteins and two ATP synthases, have important roles in the mobilization of carbohydrates and energy, and in the balance between synthesis and degradation of other proteins during seed deterioration. The up-regulation of argininosuccinate synthase participated in proline biosynthesis at 16% MC, which is important for maintaining reactive oxygen species homeostasis for the resistance of heat stress. In summary, heat-responsive protein species and mitochondrial respiratory metabolism were sensitive to high temperature and MC treatment. These studies provide a new insight into acclimation and tolerance to heat stress in oat seeds.

## Introduction

Crop seeds may deteriorate and lose their ability to germinate following storage at elevated temperatures and for extended periods of time. Seed deterioration (SD) is defined as deteriorative alterations occurring with time, increasing the vulnerability of seeds to external challenges and decreasing the ability of seeds to survive (Shaban, 2013). SD occurs during prolonged storage and escalates when seeds are stored under improper conditions, especially at high temperature and moisture content (MC) (McDonald, 1999; Arc et al., 2011). In agriculture, deteriorated seed germinates poorly and negatively affects seedling growth and yield. Optimum storage conditions have been profitable in delaying the rate of SD and in conserving for the long term the value of germplasm resources. For orthodox seeds, low temperature and MC were helpful in prolonging storage lifespan (Walters et al., 2005), while high temperature and humidity induced and accelerated the SD process (El-Maarouf-Bouteau et al., 2011). Some researchers observed that SD caused series deleterious changes within cells, such as protein denaturation, destruction of the nucleic acid synthesis system and damage of the cell membrane, eventually leading to the irreversible loss of seed vigor (Abdulbaki, 1980; Bewley et al., 2013; Wojtyla et al., 2016). However, the effects of increased seed MC and storage temperature, or the mutual relationship between them, on the deterioration process needs to be clarified.

According to the “free radical theory of aging”, reactive oxygen species (ROS) activity is considered to be the cause of alterations occurring during seed aging. Lipid peroxidation in dry seeds may occur as a result of non-enzymatic processes operating under low MC. However, when seeds are hydrated to a certain extent, biochemical processes including enzymatic reactions and respiratory activities also occur. Malondialdehyde (MDA) is a final product of lipid peroxidation, which accumulates with the enhancement of SD under higher environmental temperature in many crops, such as *Brassica napus* (Yin et al., 2015), cotton (*Gossypium hirsutum*) (Goel and Sheoran, 2003), sunflower (*Helianthus annuus*) (Kibinza et al., 2006), and soybean (*Glycine max*) (Sung and Chiu, 1995). Zhang et al. (2011) observed that MDA content was poorly correlated with seed vigor in *Elymus canadensis* and *Elymus sibiricus* at 42°C, which suggested that lipid peroxidation might not play an important role in the SD. The changes in MDA content varied with species, seed moisture and environmental temperature. Thus, MC seems to be a key factor during SD (Kibinza et al., 2006).

Kong et al. (2015) indicated that some enzymatic antioxidants could protect against oxidative stress in stored oat (*Avena sativa*) seeds with low MC, but the activities of these enzymes were limited at high MC, and proline appeared to play a more important role in response to oxidative stress. Furthermore, several researchers have demonstrated that proline accumulates in large quantities in response to high-temperature stress (Kishor et al., 2005; Verbruggen and Hermans, 2008), and that proline content increases significantly while seed vigor declines during SD in plants such as *Scorzonera pusilla* (Tian et al., 2007) and rice (*Oryza sativa*) (Momayezi et al., 2009). In addition to acting as an osmolyte for osmotic adjustment, proline contributes to radical detoxification and enzyme protection in plants and seeds under stress conditions (Ashraf and Foolad, 2007).

Proteomic technology has been widely applied to systematic research on responses to all kinds of abiotic stress, such as salt, drought, cold, and heat (Zhang et al., 2013). Some researchers have focused on the relationship between storage proteins and seed vigor and have implied that a lack of storage proteins could result in the loss of seed vigor. Heat shock proteins (HSPs) accumulating during storage or under heat stress play a prominent role in seed germination and survival (Liu et al., 2001). Xin et al. (2011) successfully identified 22 differentially expressed proteins related to energy, disease/defense and storage from the maize (*Zea mays*) embryo proteome under different conditions of seed vigor. Changes in seed vigor were affected by many factors, including energy metabolism, the defense system and the synthesis of biological macromolecules in cells. Although, these studies have provided a great deal of information about seed vigor under heat stress, the key biochemical processes essential for determining the relationship among SD, MC, and storage temperature are still unknown.

Oat is the fifth most widespread cultivated crop for grain production in the world. Oat seeds contain high concentrations of nutrients including amino acids, crude fat and soluble dietary fiber, proven to prevent coronary heart disease and reduce serum cholesterol (Berg et al., 2003; Chen and Yen, 2007). Oat seeds with higher lipid content deteriorate more easily (Pekka et al., 2003), causing great economic losses. Grain yield and nutritional characteristics, as well as seed vigor, of oat can be affected by environmental conditions such as extreme temperature during storage (Peterson et al., 2005). Therefore, it is necessary to obtain a comprehensive understanding of the mechanisms underlying SD in oat seeds. Unfortunately, investigation of this topic in oat seeds is very limited. To redress this, a proteomics approach and SD treatment were adopted to determine the relationship between seed vigor and the proteome. Seed samples with safe (10%) and high (16%) MC were utilized for heat-stress treatments at 35, 45, and 50°C, with the aim of analyzing the differences in seed vigor, physiological characteristics and protein expression levels under heat stress.

## Materials and methods

### Plant material

Oat (“Triple crown”) seeds were supplied by the Forage Seed Laboratory of China Agricultural University, Beijing, China. The original MC and germination percentage of the oat seeds were 9.0 and 93%, respectively. The experiment was initiated in May 2014. Oat seeds were moistened and attained the safe MC (10%) and high MC (16%) according to the humid atmosphere method (Rodo and Filho, 2003). Samples were then immediately packaged in hermetically sealed aluminum foil bags with a high-power electric sealer (SINBO DZ-280/2SD, Hong Kong, China).

### Heat-stress treatments

Seed samples with 10% MC in aluminum foil bags were placed in water baths (CU-600, Nanjing, China) at 35, 45, and 50°C, respectively, for 24 days. Seed samples with 16% MC were placed in water baths at 35, 45, and 50°C, respectively, for 2 days. All experiments were performed with three biological replicates. After the heat-stress treatments, the seed samples were frozen in liquid nitrogen immediately and stored at −80°C for further analysis.

### Germination test

A germination test was performed within 1 h after the completion of the heat-stress treatment. Three replications of 50 seeds for each treatment were distributed in plastic boxes (11.5 × 11.5 × 2.5 cm) containing two sheets of filter paper moistened to saturation with distilled water. Plastic boxes were put in a germination chamber (GXZ, Ningbo, China) at a constant temperature of 20°C, 60% relative humidity, 8 h light and 16 h darkness. Normal seedlings were recorded every day following incubation until 10 days; germination percentage and germination index were evaluated according to criteria in the International Rules for Seed Testing (ISTA, 2013).

### Determination of MDA and proline content

The MDA content was determined as described by Sofo et al. (2004). Powdered seeds (about 500 mg) were suspended in 5 mL of 5% (w/v) trichloroacetic acid, and the material was centrifuged at 10000 × g for 10 min at 4°C. Three milliliters of 0.06% (w/v) 2-thiobarbituric acid was added to the 3 mL of resulting supernatant, and the mixture was boiled for 15 min. The MDA content was quantified by measuring the absorbance at 450, 532, and 600 nm.

The proline content was determined as described by Momayezi et al. (2009). Powdered seeds (about 300 mg) were suspended in 3 mL of 3% (w/v) 5-sulfosalicylic acid, and the material was boiled for 10 min with shaking and centrifuged at 1000 × g for 10 min at room temperature. After the addition of 2.5% (w/v) acid ninhydrin, the supernatant was heated at 100°C for 30 min in a water bath; the reaction was then stopped by immersion in an ice bath. The mixture was extracted with toluene and the absorbance was measured at 520 nm.

### Protein extraction

Total proteins were extracted by the phenol extraction method (Faurobert et al., 2007). Three individual seed samples (1 g) were homogenized in liquid nitrogen. The powdered seeds were suspended in 3 mL extraction buffer and then an equal volume of tris-buffered phenol was added. The phenolic phase at the top of the tube was recovered by centrifuging (6000 × g for 10 min at 4°C) and then precipitated overnight at −20°C with four volumes of cooled precipitation solution (methanol containing 0.1 M ammonium acetate). Following centrifugation (6000 × g for 10 min at 4°C), the pellet recovered was rinsed with cooled precipitation solution and 80% (w/v) acetone containing 0.07% (w/v) 2-mercaptoethanol. Finally, the pellet was air-dried and resuspended in solubilization buffer {7 M urea, 2 M thiourea, 40 mM Tris, 4% (w/v) 3-[(3-cholamidopropyl) dimethylammonio]-1-propane sulfonate (CHAPS), 1% (w/v) dithiothreitol (DTT), 1 mM EDTA}. Protein concentrations in extracts were determined by the Bradford method (Li et al., 2013), using bovine serum albumin as the standard. The quantified protein samples were stored at −20°C until further use.

### Two-dimensional electrophoresis

Proteins were first separated by isoelectric focusing (IEF). For preparative IEF, IPG strips (24 cm, pH 3-10, nonlinear; GE Healthcare, Uppsala, Sweden) were passively rehydrated overnight with 450 μl of rehydration buffer [7 M urea, 2% (w/v) CHAPS, 2% (w/v) DTT, 0.7% (v/v) IPG buffers at pH 3–10, 0.001% bromophenol blue] containing 1 mg of proteins. IEF was performed at 20°C in an Ettan IPGphor 3 IEF system (GE Healthcare) for a total run of 100 kVh (50 V for 6.5 h, 500 V for 2 h, 1 kV for 2 h, 3 kV for 2 h, 5 kV for 2 h and 10 kV for 9 h). After IEF, the IPG strips were incubated in equilibration buffer [1.5 M Tris-HCl (pH 8.8), 6 M urea, 30% (v/v) glycerol, 2% (w/v) sodium dodecyl sulfate (SDS), and 0.002% bromophenol blue with 1% (w/v) DTT or 2.5% (w/v) iodoacetamide]. The second-dimension electrophoresis was carried out using SDS-polyacrylamide gels (13%, w/v, acrylamide) in an Ettan DALTsix electrophoresis Unit (GE Healthcare) until the dye line reached the end of the gel.

Gels were stained overnight with Coomassie Brilliant Blue (PhastGel Blue R-350; GE Healthcare, Uppsala, Sweden) and scanned at 300 dpi resolution using an image scanner (ImageScanner III; GE Healthcare). Image analysis was carried out with Image Master 2D Platinum 7.0 software (GE Healthcare). Each sample was analyzed at three biological replicates. To compensate for subtle variability resulting from sample loading and gel staining, spot volumes were normalized as a percentage of the total volume of all spots on the gel. Only spots with a significant difference (*p* < 0.05) were considered as varying spots, and the protein spots with an abundance ratio of at least 1.5-fold between control and heat-treated samples were selected as differentially expressed proteins and identified by MS analysis.

### Protein identification by nanoLC-MS/MS

The identified protein spots were excised from gels and distained with 50 mM ammonium bicarbonate in 50% acetonitrile, then dehydrated in 100% acetonitrile. The gels pieces were rehydrated with digestion solution (10 mg/ml trypsin in 50 mM ammonium) for 16 h or overnight at 37°C. The supernatant were vacuum-dried and dissolved in 30 mL 30% acetonitrile and 0.1% formic acid, shake for 30 min, and repeat with 60% acetonitrile. Dry the combined supernatant in vacuum concentrator, and add 10 mL 0.1% formic acid to reconstitute the residue. The digested fragments were analyzed using nanoelectrospray tandem mass spectrometry (nanoLC-MS/MS). The nanoLC separation was achieved with a Waters (Milford, MA, USA) nanoACQUITY Nano HPLC. Nanospray ESI-MS was then performed on a Thermo Q-Exactive high-resolution mass spectrometer (Thermo Scientific, Waltham, MA, USA). The MS/MS scans were acquired in data dependent mode. Survey scans (m/z 300–2000) were acquired with the resolution adjusted to 70,000 FWHM. The 10 most intensive peptide signals from the full scan were selected for MSMS scans. Analyses of MS/MS scans were performed with FWHM set at 17,500. The dynamic exclusion parameter was set to 10 s. Raw data from the mass spectrometer were preprocessed with Mascot Distiller 2.5 for peak picking. The resulted peak lists were searched against Swissprot database using Mascot 2.5 search engine compared against green plant EST database of the NCBInr protein database. The search parameters are as follows: fixed modifications: carbamidomethyl (C), variable modifications: oxidation (M); decoy database: yes; enzyme: trypsin; max missed cleavages: 2; MS mass tolerance: 10 ppm; MSMS mass tolerance: 0.02 Da. For all proteins, the best match was based on the one peptide sequences with an individual score above 21, and the coverage of the protein by the matching peptides must reach a minimum of 5%. Theoretical molecular masses and isoelectric points of the identified proteins were predicted by the ExPASy server (http://www.expasy.org/protparam).

### Functional annotation and classification

All the identified proteins were used for gene ontology (GO) and cluster of orthologous groups of proteins (COG) annotation to determine the functional classification and biological properties. GO annotation was performed using BLAST2GO (http://www.blast2go.com; Conesa and Götz, 2008). The identified proteins were classified into three functional categories: cellular component, biological process, and molecular function. COG analysis was conducted using the NCBI database (http://www.ncbi.nlm.nih.gov/COG/). In addition, pathway annotation was performed using the Kyoto Encyclopedia of Genes and Genomes (KEGG) database (http://www.kegg.jp/kegg/pathway.html).

### Hierarchical cluster analysis of protein abundance

Protein abundance values were estimated using Image Master 2D Platinum 7.0 software (GE Healthcare). Each sample was analyzed at three biological replicates. Hierarchical cluster analysis was performed using MeV (version 4.9) software after log2 transformation of the expression abundance values.

### Statistical analysis

All experiments were replicated three times independently. Vigor parameter, physiological parameters and spot intensities were all analyzed statistically using analysis of variance (ANOVA) by SPSS 18.0. Treatment means were separated using Duncan's multiple range test taking *p* < 0.05 as significant.

## Results

### Effect of heat-stress treatments on vigor of oat seeds with 10% and 16% MC

**Figure 1:**
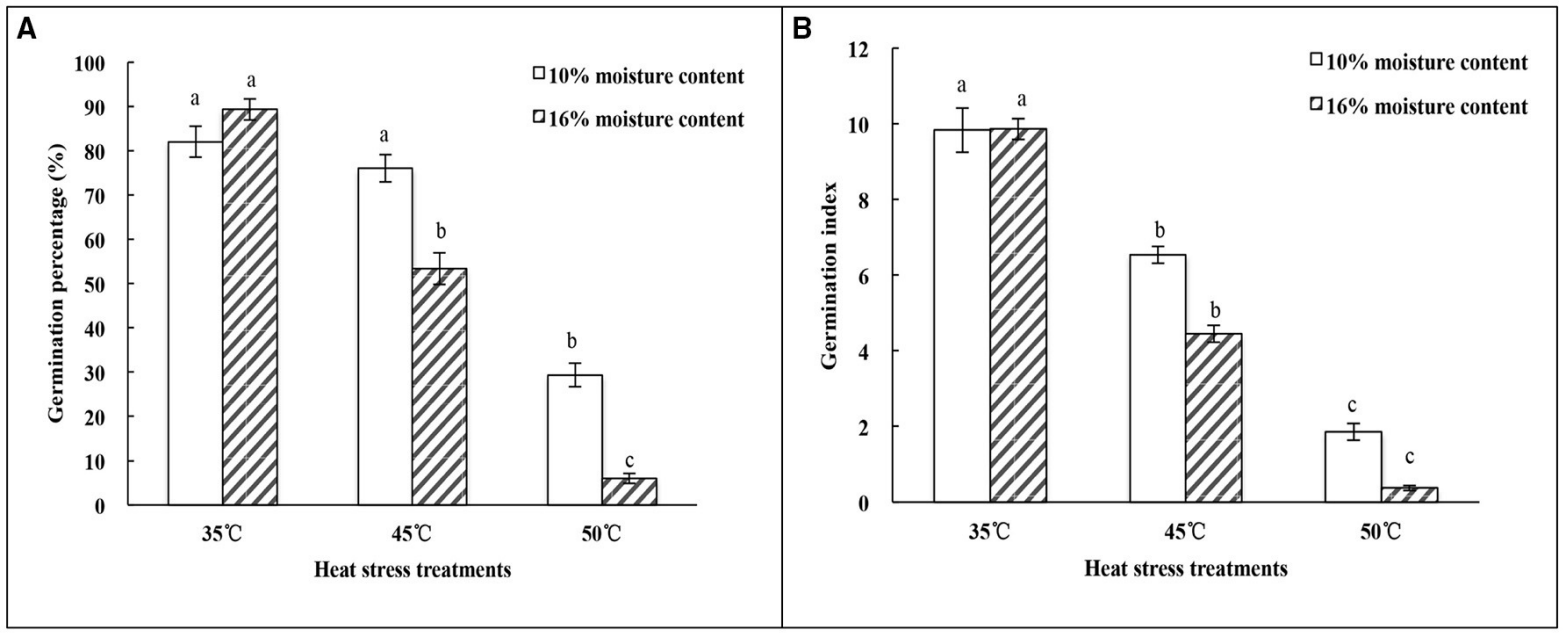
**Effect of heat-stress treatments on the vigor of oat seeds with 10 and 16% MC. (A)** Germination percentage; **(B)** Germination index. Means with different lowercase letters show significant differences at the 0.05 level among treatments at the same MC, as determined by the Duncan's multiple range test. Each bar is the mean ± SE (*n* = 3) for each treatment.

The germination percentage of oat seeds showed a trend toward decrease with temperature increase from 35 to 50°C (Figure 1A). Germination percentage of oat seeds with 16% MC declined significantly (*p* < 0.05) with increasing temperature of the heat treatments. For seeds with 10% MC, there was no significant difference (*p* > 0.05) in germination percentage between heat treatments of 35 and 45°C. The germination index of seeds with 10 and 16% MC showed a similar tendency to decline significantly (*p* < 0.05) under heat treatments from 35 to 50°C (Figure 1B).

### Effect of heat-stress treatments on MDA and proline content of oat seeds with 10 and 16% MC

**Figure 2:**
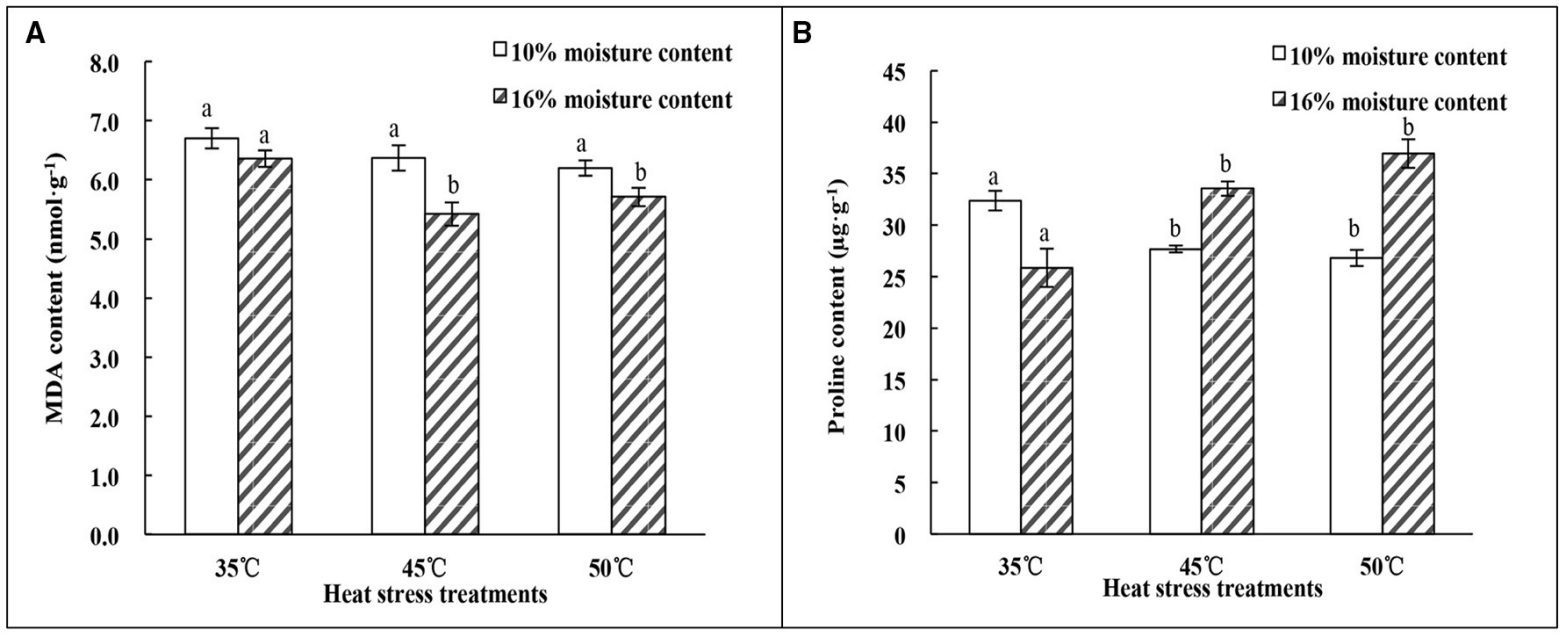
**Effect of heat stress treatments on the MDA and proline content of oat seeds with 10 and 16% MC. (A)** MDA content; **(B)** Proline content. Means with different lowercase letters show significant differences at the 0.05 level among treatments at the same MC, as determined by the Duncan's multiple range test. Each bar is the mean ± SE (*n* = 3) for each treatment.

The results of MDA content analysis showed different tendencies under heat treatments for seeds with different MC. There was no significant (*p* > 0.05) difference in MDA content of seeds with 10% MC as the temperature increased from 35 to 50°C. However, the MDA content of seeds with 16% MC showed a significant (*p* < 0.05) decrease at temperatures of 45 and 50°C; there was no significant (*p* > 0.05) difference between these temperatures (Figure 2A). MDA content in 16% MC seeds was lower than that in 10% MC seeds under heat-stress treatment.

The changes in proline content presented the opposite trend with heat treatments from 35 to 50°C (Figure 2B). The proline content of seeds with 10% MC decreased significantly (*p* < 0.05) as the temperature increased to 45°C, and no significant difference (*p* > 0.05) was found between 45 and 50°C. However, the proline content in seeds with 16% MC increased significantly (*p* < 0.05) as the temperature increased to 45°C. There was also no significant difference (*p* > 0.05) found between 45 and 50°C.

### Changes in protein abundance of oat seeds with 10 and 16% MC under heat-stress treatments

From the three 2D-gel replicates, a total of 94 and 32 protein spots were detected in oat seeds with 10 and 16% MC, respectively, and these showed statistically significant differences at abundance ratios over 1.5-fold in relation to heat-stress treatment. Twenty-one proteins were successfully identified by searching against the MASCOT database (Table 1, Supplementary Figure 1). Most of these proteins covered the isoelectric points that ranging from 5 to 7, and their molecular masses ranged from 10 to 20 kDa, indicating that weakly acidic proteins with molecular masses of between 10 and 20 KDa play very important roles in the deteriorated oat seeds. Similar changes were observed between experimental and theoretical isoelectric point and molecular mass, except for spot 31 and spot 33 (Supplementary Figure 2).

**Table 1:** **List of differentially expressed proteins identified by LC-MS/MS in oat seeds with 10 and 16% MC under heat stress**.

**Spot**	**ANOVA (*P*-value)**	**Protein name**	**Species**	**Accession no.^a^**	**Score^b^**	**Coverage (%)^c^**	**Theor Mw/pI^d^**	**Exper Mw/pI^e^**
**10% MOISTURE CONTENT**								
**Response to stimulus**								
51	0.037	ADP-ribosylation factor 1	*Chalamydomonas reinhardtii*	gi|1703374	475	35.9	20.70/6.92	20.75/6.92
78	0.019	18.3 kDa class I heat shock protein	*Oxybasis rubra*	gi|462322	966	20.5	18.27/6.76	18.26/6.77
92	0.007	Late embryogenesis abundant protein B19.3	*Hordeum vulgare*	gi|547818	526	43.6	14.60/5.38	14.60/5.38
99	0.020	16.9 kDa class I heat shock protein 1	*Triticum aestivum*	gi|123545	1179	34.4	16.88/5.83	16.87/5.83
515	0.033	EMB-1 protein	*Daucus carota*	gi|119316	47	14.1	9.92/6.74	9.91/6.74
758	0.030	Luminal-binding protein 2	*Zea mays*	gi|6016150	434	18.7	73.08/5.10	73.21/5.10
889	0.024	ATP synthase subunit d, mitochondrial	*Arabidopsis thaliana*	gi|25089786	268	26.0	19.59/5.09	19.57/5.09
**Metabolic process; Cellular process**								
46	0.049	14 kDa zinc-binding protein	*Zea mays*	gi|162464249	1540	24.2	14.30/6.19	14.35/6.19
51	0.037	ADP-ribosylation factor 1	*Chalamydomonas reinhardtii*	gi|1703374	475	35.9	20.70/6.92	20.75/6.92
106	0.029	Eukaryotic translation initiation factor 1A	*Triticum aestivum*	gi|1352427	938	27.1	16.29/5.07	16.45/5.07
515	0.033	EMB-1 protein	*Daucus carota*	gi|119316	47	14.1	9.92/6.74	9.91/6.74
758	0.030	Luminal-binding protein 2	*Zea mays*	gi|6016150	434	18.7	73.08/5.10	73.21/5.10
889	0.024	ATP synthase subunit d, mitochondrial	*Arabidopsis thaliana*	gi|25089786	268	26.0	19.59/5.09	19.57/5.09
**Cellular component**								
99	0.020	16.9 kDa class I heat shock protein 1	*Triticum aestivum*	gi|123545	1179	34.4	16.88/5.83	16.87/5.83
758	0.030	Luminal-binding protein 2	*Zea mays*	gi|6016150	434	18.7	73.08/5.10	73.21/5.10
889	0.024	ATP synthase subunit d, mitochondrial	*Arabidopsis thaliana*	gi|25089786	268	26.0	19.59/5.09	19.57/5.09
**Biological regulation; Regulation of biological process**								
51	0.037	ADP-ribosylation factor 1	*Chalamydomonas reinhardtii*	gi|1703374	475	35.9	20.70/6.92	20.75/6.92
92	0.007	Late embryogenesis abundant protein B19.3	*Hordeum vulgare*	gi|547818	526	43.6	14.60/5.38	14.60/5.38
515	0.033	EMB-1 protein	*Daucus carota*	gi|119316	47	14.1	9.92/6.74	9.91/6.74
**Developmental process; Reproduction; Reproductive process; Multicellular organismal process**								
92	0.007	Late embryogenesis abundant protein B19.3	*Hordeum vulgare*	gi|547818	526	43.6	14.60/5.38	14.60/5.38
515	0.033	EMB-1 protein	*Daucus carota*	gi|119316	47	14.1	9.92/6.74	9.91/6.74
**Signaling; Localization**								
51	0.037	ADP-ribosylation factor 1	*Chalamydomonas reinhardtii*	gi|1703374	475	35.9	20.70/6.92	20.75/6.92
515	0.033	EMB-1 protein	*Daucus carota*	gi|119316	47	14.1	9.92/6.74	9.91/6.74
**Establishment of localization**								
51	0.037	ADP-ribosylation factor 1	*Chalamydomonas reinhardtii*	gi|1703374	475	35.9	20.70/6.92	20.75/6.92
**Unclassified proteins**								
33	0.033	Avenin	*Avena sativa*	gi|75220602	509	23.8	25.47/8.15	24.67/7.53
71	0.008	17.9 kDa class II heat shock protein	*Helianthus annuus*	gi|1170365	243	25.0	17.85/7.70	17.96/7.71
362	0.047	12S Seed storage globulin 2	*Avena sativa*	gi|134919	1023	33.2	58.67/7.64	59.04/7.64
**16% MOISTURE CONTENT**								
**Response to stimulus**								
15	0.028	Em protein CS41	*Triticum aestivum*	gi|119315	7145	41	9.99/5.29	9.98/5.28
90	0.050	17.3 kDa class I heat shock protein	*Glycine max*	gi|123534	780	29	17.35/6.17	17.34/6.17
95	0.046	18.3 kDa class I heat shock protein	*Oxybasis rubra*	gi|462322	966	20.5	18.27/6.76	18.26/6.77
96	0.039	17.9 kDa class I heat shock protein	*Oryza sativa subsp. Japonica*	gi|75298023	2926	19	17.91/5.79	17.90/5.80
963	0.048	ATP synthase subunit alpha, mitochondrial	*Oryza sativa*	gi|148886790	943	20	55.37/5.85	55.62/5.85
**Metabolic process; Cellular process**								
446	0.036	Argininosuccinate synthase, chloroplastic	*Arabidopsis thaliana*	gi|78099761	150	12	53.84/6.25	54.15/6.25
963	0.048	ATP synthase subunit alpha, mitochondrial	*Oryza sativa*	gi|148886790	943	20	55.37/5.85	55.62/5.85
**Developmental process; Reproduction; Reproductive process; Multicellular organismal process**								
15	0.028	Em protein CS41	*Triticum aestivum*	gi|119315	7145	41	9.99/5.29	9.98/5.28
**Localization; Establishment of localization**								
963	0.048	ATP synthase subunit alpha, mitochondrial	*Oryza sativa*	gi|148886790	943	20	55.37/5.85	55.62/5.85
**Unclassified proteins**								
31	0.014	Avenin	*Avena sativa*	gi|75220602	509	23.8	25.47/8.15	24.67/7.53
100	0.043	12S Seed storage globulin 1	*Avena sativa*	gi|134918	612	16	58.54/8.78	58.96/8.78
412	0.048	12S Seed storage globulin 2	*Avena sativa*	gi|134919	1023	33.2	58.67/7.64	59.04/7.64

Proteins are grouped by biological process of GO functional annotation. Theor., theoretical; Exper., experimental.

a Accession number from the NCBI database.

b MASCOT protein score from the LC-MS/MS analysis.

c Sequence coverage percentage.

d Theoretical molecular mass/isoelectric point.

e Experimental molecular mass/isoelectric point.

**Figure 3:**
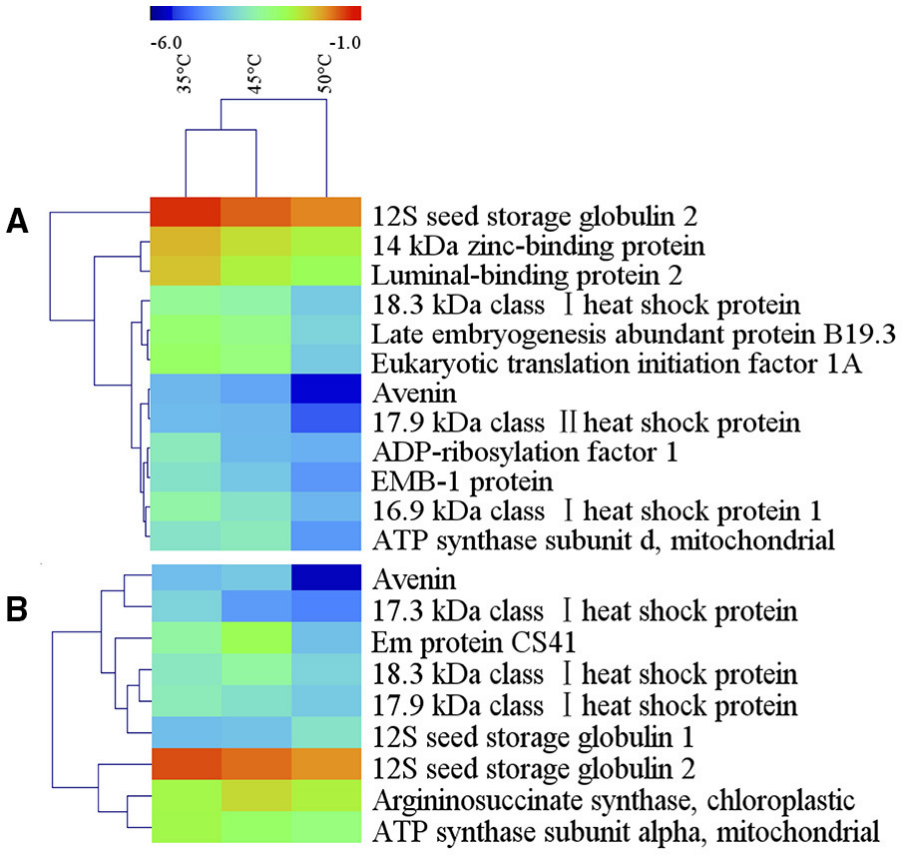
**Hierarchical cluster analysis of heat-resistance proteins in oat seeds with 10 and 16% MC**. Dataset clustering was performed with MeV software after log2 transformation of the expression abundance values. Each colorized cell represents the spot quantity, according to the color scale at the top of the figure. (**A**, 10% MC; **B**, 16% MC).

**Figure 4:**
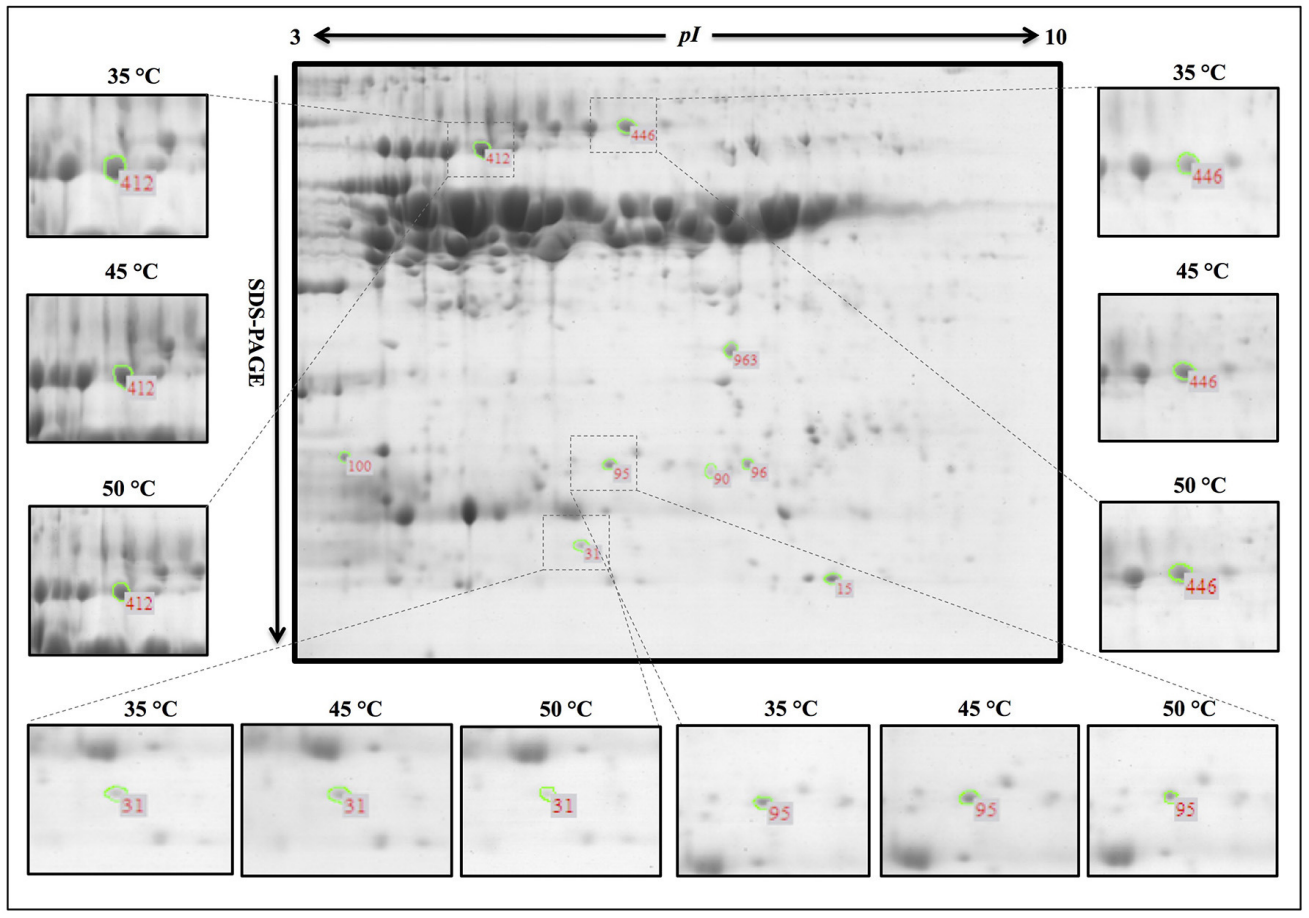
**Representative 2-DE pattern of the oat seed proteome under heat stress**. Spot numbers indicate the protein spots analyzed by Image Master 2D Platinum 7.0 software. Zoomed images show the main differentially expressed protein spots in deteriorated oat seeds. Spot 446, up-regulation in oat seeds with 16% MC after heat-stress treatment; spots 31, 95, 412, down-regulation in oat seeds with 10 and 16% MC after heat-stress treatment. The three biological replicates of the 2-DE gels are shown in Supplementary Figure 1.

Among the differentially accumulated protein spots, there were 94 protein spots altered significantly (*p* < 0.05) and 12 protein spots further identified by nanoLC-MS/MS in 10% MC seeds (Table 1). According to their expression patterns, these 12 spots could be grouped into four types (Figure 3): type I, spots decreased slowly and then dropped rapidly with temperature increasing from 35 to 50°C (spots 33, 71, 78, 92, and 106); type II, spots first decreased rapidly and then slowly (spots 51 and 758); type III, spots were down-regulated consistently with temperature increases from 35 to 50°C (spots 46, 99, 362, and 515); type IV, spots were up-regulated as the temperature increased from 35 to 45°C and down-regulated from 45 to 50°C (spot 889). For oat seeds with 16% MC, a total of 32 protein spots were altered significantly (*p* < 0.05) and there were nine protein spots further identified by nanoLC-MS/MS analysis (Table 1). These nine spots grouped into three types (Figure 3): type I, spots increased with temperature from 35 to 45°C and decreased from 45 to 50°C (spots 15, 31, 95, and 446); type II, spots were down-regulated consistently with temperature increases from 35 to 50°C (spots 90, 96, 412, and 963); type III, spots were up-regulated consistently with temperature increases from 35 to 50°C (spot 100). Interestingly, three down-regulated spots (spots 31, 95, and 412) were also identified in oat seeds with 10% MC (spots 33, 78, and 362), and were also down-regulated as the temperature increased from 35 to 50°C (Figure 4).

### Functional annotation and classification of proteins identified in oat seeds with 10 and 16% MC

**Figure 5:**
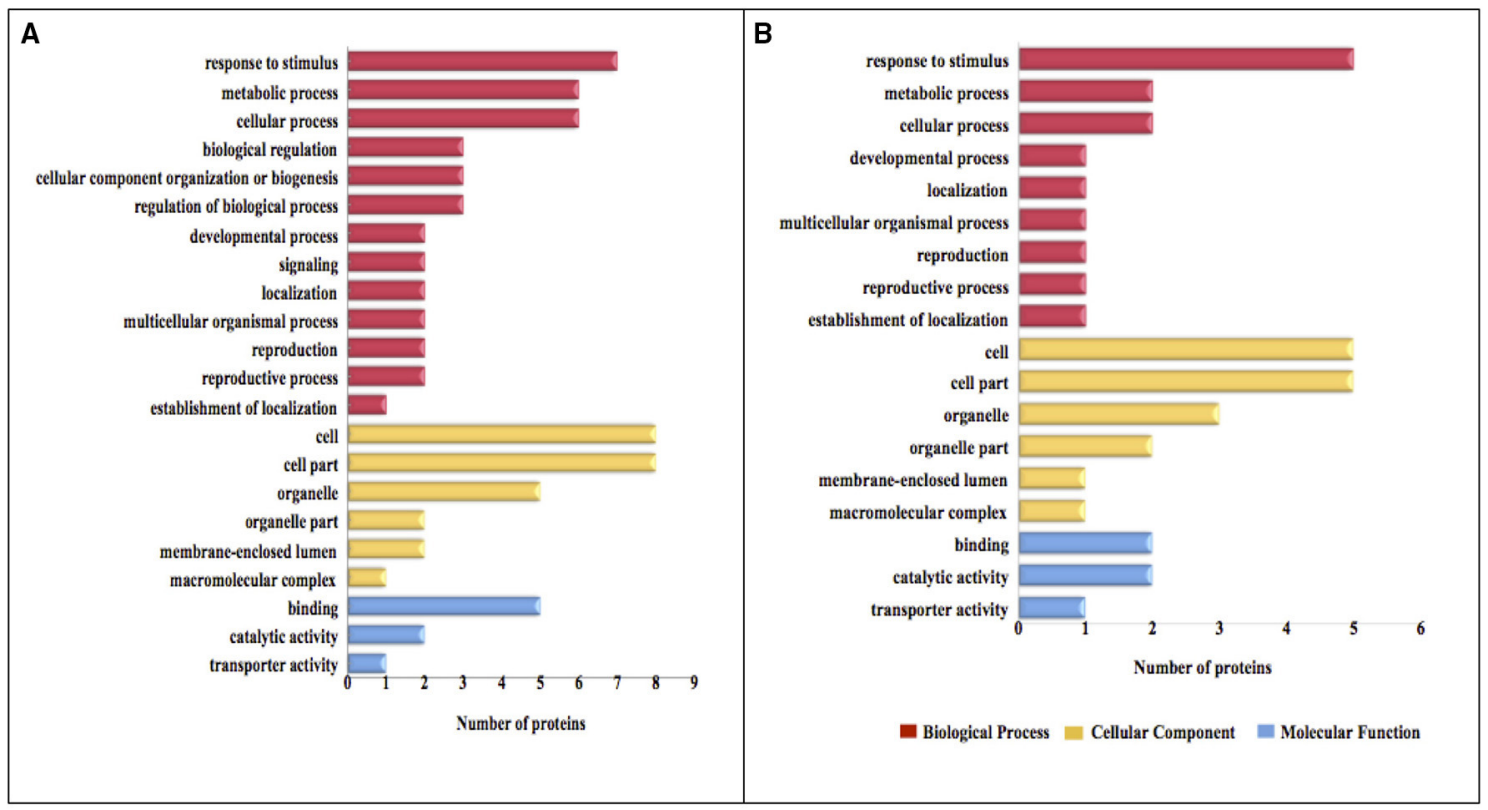
**GO categories of the identified proteins in oat seeds with 10 and 16% MC under heat stress**. Results are summarized under three main GO categories: biological process, cellular component, and molecular function. (**A**, 10% MC; **B**, 16% MC).

**Figure 6:**
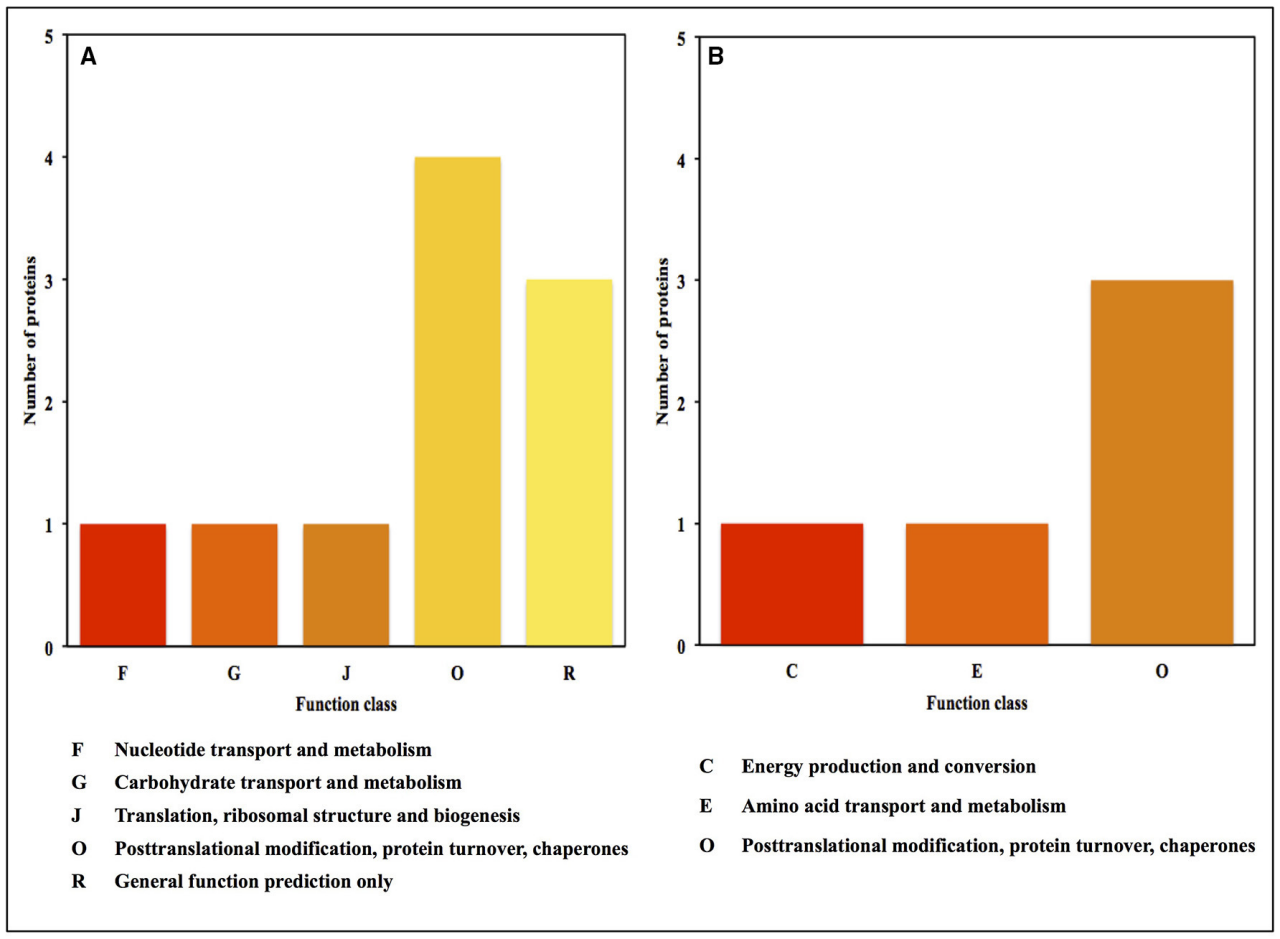
**COG functional classification for the proteins identified in oat seeds with 10 and 16% MC under heat stress**. (**A**, 10% MC; **B**, 16% MC).

The functional categories of differential expression proteins were determined using GO analysis (Figure 5) and COG analysis (Figure 6). For oat seeds with 10% MC after heat treatment, a total of 10 proteins were divided into 22 GO terms to describe biological processes, cellular components, and molecular functions. The major functional categories in the biological processes were response to stimulus (7; 17.07%), cellular processes (6; 14.63%) and metabolic processes (6; 14.63%). Among cellular components, cell (8; 30.77%) and cell part (8; 30.77%) were the most abundant groups, whereas binding (5; 62.50%) and catalytic activity (2; 25.00%) accounted for the most abundant groups in terms of molecular functions (Figure 5A). Proteins were also aligned to the COG database to predict and classify possible functions. There were eight proteins distributed into five COG categories (Figure 6A, Supplementary Table 1), of which the COG category “posttranslational modification, protein turnover, chaperones” represented the largest group (3; 37.5%), followed by “general function prediction only” (2; 25%); the smallest groups were “nucleotide transport and metabolism” (1; 12.5%), “carbohydrate transport and metabolism” (1; 12.5%) and “translation, ribosomal structure and biogenesis” (1; 12.5%). A total of seven proteins were assigned to eight pathways by the KEGG analysis (Supplementary Table 2). Among these pathways, protein processing in endoplasmic reticulum and disease-related metabolic pathways were the highlighted pathways.

For oat seeds with 16% MC, there were eight proteins to be classified into 18 GO terms to describe biological processes, cellular components, and molecular functions. The major functional categories in the biological processes were response to stimulus (5; 33.33%), cellular processes (2; 13.33%) and metabolic processes (2; 13.33%). Among cellular components, cell (5; 29.41%) and cell part (5; 29.41%) were the most abundant groups, whereas binding (2; 40.00%) and catalytic activity (2; 40.00%) accounted for the most abundant groups in terms of molecular functions (Figure 5B). According to COG function annotation, five proteins were distributed into three COG categories (Figure 6B, Supplementary Table 1), among which COG category “posttranslational modification, protein turnover, chaperones” represented the largest group (3; 60%), followed by “energy production and conversion” (1; 20%) and “amino acid transport and metabolism” (1; 20%). Notably, these five proteins, including ATP synthase subunit alpha, argininosuccinate synthase (ASS), and 17.3, 17.9, and 18.3 kDa class I heat shock proteins, were also mapped in the 10 KEGG pathways (Supplementary Table 2). This indicated that most of these related proteins might be playing an important role in the deterioration of oat seeds.

## Discussion

### High temperature and seed MC accelerated SD

Temperature and MC are two important environmental factors that influence seed longevity. For the longevity of orthodox seeds in storage, the optimal MC was shown to be 10–11% with a temperature lower than 25°C (Ellis et al., 1989). In this experiment, the decrease of seed germination percentage and germination index in oat seeds with 10 or 16% MC as temperature was raised from 45 to 50°C confirmed the occurrence of SD. Furthermore, seed vigor at 16% MC declined much faster than that at 10% MC under heat stress (Figure 1). This meant that there was a synergy between higher temperature and MC affecting seed vigor during storage. Xia et al. (2015) observed that the germination percentage of oat seeds decreased gradually during seed aging, and the extent of the declining rate of germination was related to seed MC. Moreover, Kong et al. (2015) observed that the germination percentage of oat seeds decreased with increasing seed MC. When in storage for 12 months, the germination percentage of oat seeds with 4% MC did not change significantly, but decreased significantly in oat seeds with 16% MC and reached zero in seeds with 28% MC. In this study, similar results were obtained in oat seeds during heat stress.

Although, the biochemical mechanisms of SD are not yet fully understood, several studies identified oxidative free radical attack as one of the main causes of SD during seed storage (Galleschi et al., 2002). In addition, some researchers found that vigor loss or seed death was well correlated with accumulation of MDA in plant species such as neem (*Azadirachta indica*) (Kumar and Mishra, 2014), cotton (Goel and Sheoran, 2003) and onion (*Allium cepa*) (Rao et al., 2006). However, MDA content declined under the combination of higher temperature and MC conditions in this study; there were no significant (*p* > 0.05) differences in MDA content among heat treatments in oat seeds with 10% MC. A significant decrease in MDA content was observed at 45°C in 16% MC seed (Figure 2A). Similar results have been reported for wheat (*Triticum aestivum*) (Lehner et al., 2008). These results indicated that the loss of seed vigor might not be attributed to lipid peroxidation at higher MC (16%). Meanwhile, proline accumulation during heat treatments was only evident in 16% MC seeds. The changes in proline content between 10 and 16% MC showed the opposite tendency with temperature alteration from 35 to 50°C (Figure 2B). The accumulation of proline could be regarded as a signal of response to abiotic stress (Floyd and Nagy, 1984). It has been demonstrated that proline accumulates as plants suffer from environmental temperature stress in species such as alfalfa (*Medicago sativa*) (Li et al., 2013), chickpea (*Cicer arietinum*) leaves (Kaushal et al., 2011) and *Phylanthus emblica* seeds (Li et al., 2009). This suggests that lipid peroxidation was affected by the level of oat seed MC during storage and that MC was a primary factor in causing SD.

### Proteome expression of oat seeds with different MC under heat stress

In this study, there were 21 proteins differentially expressed in oat seeds under heat stress, and these were mainly involved in stress/disease responses, energy metabolism, carbohydrate and amino acid transport and metabolism, posttranslational modification, etc. among all heat-responsive proteins.

#### Heat shock proteins regulated with increasing temperature

Six HSPs were down-regulated in oat seeds with 10 and 16% MC during heat-stress treatment, and these were mainly involved in response to stimulus, posttranslational modification, protein turnover, chaperones, folding, sorting and degradation. Most of them were small heat shock proteins (sHSPs), including sHSP16.9 class I (spot 99), sHSP17.3 class I (spot 90), sHSP17.9 class I (spot 96), sHSP17.9 class II (spot 71), and sHSP18.3 class I (spots 78 and 95). As the most ubiquitous HSP subgroup, sHSPs have been confirmed to have molecular chaperone functions that regulate the degradation and accumulation of proteins and could mediate the correct folding of proteins to protect from damage (Waters et al., 1996). Many abiotic and biotic stresses result in endoplasmic reticulum (ER) stress (Liu et al., 2012). ER-localized sHSPs accumulate in all higher plants (Zhao et al., 2007), and these sHSPs might have important functions in protection against stress. In this study, five spots (spots 78, 90, 95, 96, and 99) were identified as class I sHSPs, while one spot (spot 71) identified as a class II sHSPs was localized in the ER (Supplementary Table 2). There were four HSPs (spots 71, 78, 99, and 758) in 10% MC oat seeds, and the protein number decreased to three HSPs (spots 90, 95, and 96) in 16% MC seeds, in which spot 758 disappeared. Also, these proteins (spots 90, 95, and 96) belonging to the class I sHSPs were only maintained in seeds with 16% MC. Luminal-binding protein 2 (spot 758) is a homolog of the HSP70 family, which facilitates the assembly of multimeric protein complexes inside the ER. The HSP70 family is a group of evolutionarily highly conserved chaperone proteins, and is involved in translocation processes, protein degradation and protein import (Wang et al., 2004). Over-expression of luminal-binding protein was able to restore efficient alpha-amylase synthesis, which might be a regulatory mechanism to alleviate ER stress (Leborgne-Castel et al., 1999).

Previous reports documented that significant up-regulation of HSPs was a key part of the heat-shock response (Xiao et al., 2009; Kosova et al., 2011). On the contrary, some HSPs were down-regulated in our study and mainly belonged to the sHSPs. It was reported that HSPs were up-regulated in short-term heat stress (Lee et al., 2007; Valcu et al., 2008) and down-regulated in long-term heat stress (Xu and Huang, 2010). Similarly, HSC70 abundance in thermal *Agrostis scabra* leaves showed a reduction after 2 days of heat stress, while abundance continued to decline after 10 days in *Agrostis stolonifera* leaves (Xu and Huang, 2010). In this study, decreases in sHSP16.9 class I and luminal-binding protein 2 were identified in oat seeds with 10% MC treated at 35, 45, and 50°C for 24 days, which suggested that a decrease in these HSPs might contribute to superior long-term thermotolerance in oat seeds. The identified sHSPs, including 17.9 and 18.3, might also contribute to the superior heat tolerance in oat seeds. The sHSP17.3 protein could regulate protein degradation when oat seeds suffer high MC and temperature, and adjust the correct folding of proteins to prevent further damage and repair intracellular injury. Therefore, we propose that a reduction in the abundance of HSPs might create a balance between synthesis and degradation of other proteins to prevent SD in oat seeds in response to heat stress and high MC.

#### Energy metabolism regulated by related proteins

High-vigor seeds could mobilize storage proteins to get sufficient energy and carbohydrate supply for germination (Yang et al., 2007). However, deteriorated seeds are less active in mobilizing their protein reserves, affecting the speed, homogeneity and final extent of germination. Correspondingly, energy production and conversion are strongly affected (Basavarajappa et al., 1991). Two ATPases, which catalyze the synthesis of ATP in the citric acid cycle of respiration metabolism, were identified in the proteomic analysis, including ATP synthase subunit delta (spot 889) in 10% MC seeds and ATP synthase subunit alpha (spot 963) in 16% MC seeds (Table 1). ATP synthase subunits alpha and delta are the primary catalytic sites for ATPase (Jansch et al., 1996). In this study, the intensity of ATP synthase subunit delta increased while that of subunit alpha decreased as heat stress increased from 35 to 45°C, and levels of both declined from 45 to 50°C. At the same time, the vigor of oat seed at both 10 and 16% MC was lowest at 50°C. There was a positive correlation between seed vigor and ATPase activity at 16% MC, and the heat treatment at 45°C for 24 days might enhance the thermotolerance of oat seed with 10% MC. Furthermore, there was a similar report on six ATP synthase subunits that were down-regulated in maize seeds (13.58% MC) as they were artificially aged at 50°C for 5 or 13 days. Seed germinating time was delayed, and only 9.6% seeds could germinate after aging for 13 days (Xin et al., 2011). This indicated that the reduction in the abundance of ATPase in oat seeds resulted in a decreasing energy supply and limited seed germination speed.

#### Up-regulated proteins responding to heat stress

There were two proteins, including ASS (spot 446) and 12S seed storage globulin 1 (spot 100), that were up-regulated in oat seeds at 16% MC with heat treatment. According to GO, COG functional classification (Supplementary Table 1) and KEGG pathway analysis (Supplementary Table 2), ASS was classified under amino acid transport and metabolism. Proline synthesis occurs via two routes, involving glutamic acid and ornithine respectively, and ASS could catalyze the synthesis of ornithine (Kumar et al., 1985; Husson et al., 2003). It has been proven that the ornithine pathway in proline biosynthesis could be activated by salt stress in barley (*Hordeum vulgare*) seedlings (Zhao et al., 2001). Proline is well known to accumulate in large quantities in plants in response to environmental stress. Proline acts as a molecular chaperone in osmotic adjustment and protection of proteins, membranes and cellular structures during osmotic stress (Delauney and Verma, 1993). In this study, the increased proline content of oat seed with 16% MC from 35 to 45°C might be due to up-regulation of ASS (spot 446). ASS is also involved in the synthesis of arginine, alanine, aspartate and glutamate (Supplementary Table 2). The increased ASS might regulate some important metabolic pathways, and protect against a reduction of proteins that could lead to aging, division and growth of abnormal cells that ultimately results in SD. The function of ASS in the response of oat seeds to high temperature and MC needs further in-depth study.

The abundance of 12S seed storage globulin 1 (spot 100) increased with decrease in seed vigor, but this protein could not be classified according to functional annotation. The regulation of this protein needs further study in deteriorated oat seeds.

#### Stress response proteins and redox-regulated proteins during heat stress

Oxidative damage induced by abiotic stress triggers disturbances in the cellular redox balance, and a complex regulatory mechanism such as the heat-shock response was adopted to prevent cellular injury by regulating the homeostasis of ROS in aged seeds (Circu and Aw, 2010; Poehlmann et al., 2011). HSPs with chaperone activities are favored targets for oxidation and prevent cytotoxic aggregates (Fink, 1999). In this study, six HSPs with chaperone activities were involved in redox homeostasis and found to be down-regulated during heat treatment. Also, Rajjou et al. (2008) found that three HSP70 proteins with chaperone activities were favored targets for oxidation in deteriorated *Arabidopsis* seeds.

Proline is considered as a scavenger of ROS and could reduce the damage of oxidative stress induced by abiotic stress (Smirnoff and Cumbes, 1989). Under stress conditions, proline content could be modulated either through increased expression of proline synthase or inhibition of proline degradation (Hong et al., 2000). In this study, up-regulation of ASS might be efficient during stress-induced proline accumulation and ROS declination, then reduce the oxidative damage and protect oat seeds from heat stress.

It could be suggested that the combination of high temperature and MC would be conducive to the loss of vigor of oat seed, which was not the result of lipid peroxidation according to the changes observed in MDA content. The proteomics analysis showed that changes in sHSPs species and mitochondrial respiration metabolism were more sensitive to the higher temperature and MC treatments, and activated biosynthesis and accumulation of proline with related up-regulation of ASS, which played an important role in heat tolerance by maintaining ROS homeostasis. Furthermore, increasing seed MC would accelerate mitochondrial respiration under high temperature conditions; however, the relationship between respiration metabolism and proline needs to be explored further.

## Author contributions

PM designed the study and revised the manuscript. LC and QC carried out the study and wrote the manuscript. LK and FX provided advices for the study. HY and YZ carried out the bioinformatics analysis. All authors discussed the results and reviewed the manuscript.

### Conflict of interest statement

The authors declare that the research was conducted in the absence of any commercial or financial relationships that could be construed as a potential conflict of interest.

## Abbreviations

SD: seed deterioration
MC: moisture content
ROS: reactive oxygen species
MDA: malondialdehyde
2-DE: two-dimensional electrophoresis
MS: mass spectrometry
IEF: isoelectric focusing
HSPs: heat shock proteins
DTT: dithiothreitol
CHAPS: 3-[(3-cholamidopropyl) dimethylammonio] -1-propane sulfonate
SDS: sodium dodecyl sulfate
nanoLC-MS/MS: nanoelectrospray tandem mass spectrometry
GO: gene ontology
COG: cluster of orthologous groups of proteins
KEGG: Kyoto encyclopedia of genes and genomes
sHSPs: small heat shock proteins
ER: endoplasmic reticulum
ASS: argininosuccinate synthase.
