# Supplementary material for: Proteomic and Physiological Analysis of the Response of Oat (Avena sativa) Seeds to Heat Stress under Different Moisture Conditions
Source: Front Plant Sci. 2016 Jun 22;7:896. doi: 10.3389/fpls.2016.00896 (PMC4916207; doi:10.3389/fpls.2016.00896)
Supplement: Supplementary file 1 [file Table1.DOCX]

Supplementary Table 1. COG function classification of identified proteins in oat seeds with 10% and 16% moisture content under heat stress.

| **Functional Categories** | **Differentially Expressed Protein** | **Protein Number** |
| --- | --- | --- |
| **10% moisture content** |  |  |
| Nucleotide transport and metabolism | 14 kDa zinc-binding protein | 1 |
| Carbohydrate transport and metabolism | 14 kDa zinc-binding protein | 1 |
| Translation, ribosomal structure and biogenesis | Eukaryotic translation initiation factor 1A | 1 |
| Posttranslational modification,  Protein turnover, chaperones | 16.9 kDa class Ⅰheat shock protein | 4 |
|  | 17.9 kDa class Ⅱheat shock protein |  |
|  | 18.3 kDa class Ⅰheat shock protein |  |
|  | Luminal-binding protein 2 |  |
| General function prediction only | 14 kDa zinc-binding protein | 3 |
|  | ADP-ribosylation factor 1 |  |
|  | ADP-ribosylation factor 1 |  |
| Energy production and conversion | ATP synthase subunit alpha | 1 |
| Amino acid transport and metabolism | Argininosuccinate synthase, chloroplastic | 1 |
| Posttranslational modification,  Protein turnover, chaperones | 17.3 kDa class Ⅰheat shock protein | 3 |
|  | 18.3 kDa class Ⅰheat shock protein |  |
|  | 17.9 kDa class Ⅰheat shock protein |  |
