# Supplementary material for: Proteomic and Physiological Analysis of the Response of Oat (Avena sativa) Seeds to Heat Stress under Different Moisture Conditions
Source: Front Plant Sci. 2016 Jun 22;7:896. doi: 10.3389/fpls.2016.00896 (PMC4916207; doi:10.3389/fpls.2016.00896)
Supplement: Supplementary file 2 [file Table2.DOCX]

Supplementary Table 2. KEGG pathway analysis of identified proteins in oat seeds with 10% and 16% moisture content under heat stress.

| **Pathway** | **Differentially Expressed Protein** | **Protein Number** | |
| --- | --- | --- | --- |
| **10% moisture content** |  |  |  |
| *Global and overview maps* |  |  |  |
| Metabolic pathways | ATP synthase subunit d, mitochondrial | 1 | |
| *Energy metabolism* |  |  |  |
| Oxidative phosphorylation | ATP synthase subunit d, mitochondrial | 1 | |
| *Folding, sorting and degradation* | | | |
| Protein export | Luminal-binding protein 2 | 1 | |
| Protein processing in endoplasmic reticulum | 16.9 kDa class Ⅰheat shock protein 1  17.9 kDa class Ⅱheat shock protein  18.3 kDa class Ⅰheat shock protein  Luminal-binding protein 2 | 4 | |
| *Translation* | | | |
| RNA transport | Eukaryotic translation initiation factor 1A | 1 | |
| *Neurodegenerative diseases* | | | |
| Huntington's disease | ATP synthase subunit d, mitochondrial | 1 | |
| Parkinson's disease | ATP synthase subunit d, mitochondrial | 1 | |
| Alzheimer's disease | ATP synthase subunit d, mitochondrial | 1 | |
| Prion diseases | Luminal-binding protein 2 | 1 | |
| **16% moisture content** | | | |
| *Global and overview maps* | | | |
| Metabolic pathways | ATP synthase subunit alpha, mitochondrial  Argininosuccinate synthase, chloroplastic | 2 | |
| Biosynthesis of secondary metabolites | Argininosuccinate synthase, chloroplastic | 1 | |
| *Amino acid metabolism* |  |  |  |
| Alanine, aspartate and glutamate metabolism | Argininosuccinate synthase, chloroplastic | 1 | |
| Arginine and proline metabolism | Argininosuccinate synthase, chloroplastic | 1 | |
| *Energy metabolism* |  |  |  |
| Oxidative phosphorylation | ATP synthase subunit alpha, mitochondrial | 1 | |
| Photosynthesis | ATP synthase subunit alpha, mitochondrial | 1 | |
| *Folding, sorting and degradation* | | | |
| Protein processing in endoplasmic reticulum | 17.3 kDa class Ⅰheat shock protein  18.3 kDa class Ⅰheat shock protein  17.9 kDa class Ⅰheat shock protein | 3 | |
| *Neurodegenerative diseases* |  |  |  |
| Parkinson's disease | ATP synthase subunit alpha, mitochondrial | 1 | |
| Huntington's disease | ATP synthase subunit alpha, mitochondrial | 1 | |
| Alzheimer's disease | ATP synthase subunit alpha, mitochondrial | 1 | |
