# Supplementary material for: Proteomic and Physiological Analysis of the Response of Oat (Avena sativa) Seeds to Heat Stress under Different Moisture Conditions
Source: Front Plant Sci. 2016 Jun 22;7:896. doi: 10.3389/fpls.2016.00896 (PMC4916207; doi:10.3389/fpls.2016.00896)
Supplement: Supplementary file 3 [file Image1.PDF]

Supplementary Figure 1. Three biological replicates of the 2-DE gels in oat seeds with 10% and 16% moisture content under heat stress. Circles indicate the identified proteins by LC-MS/MS. (A: 10% moisture content; B: 16% moisture content).

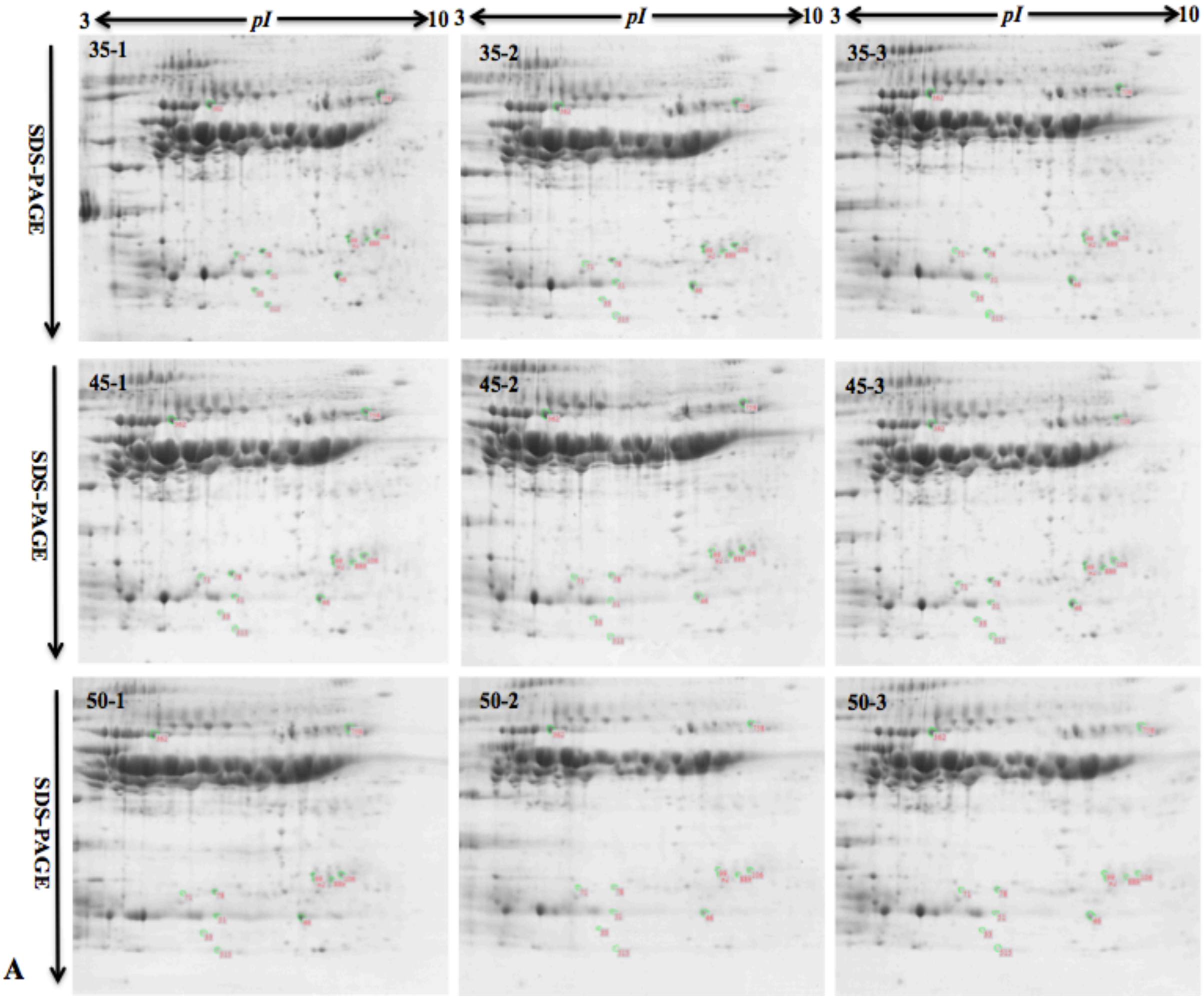

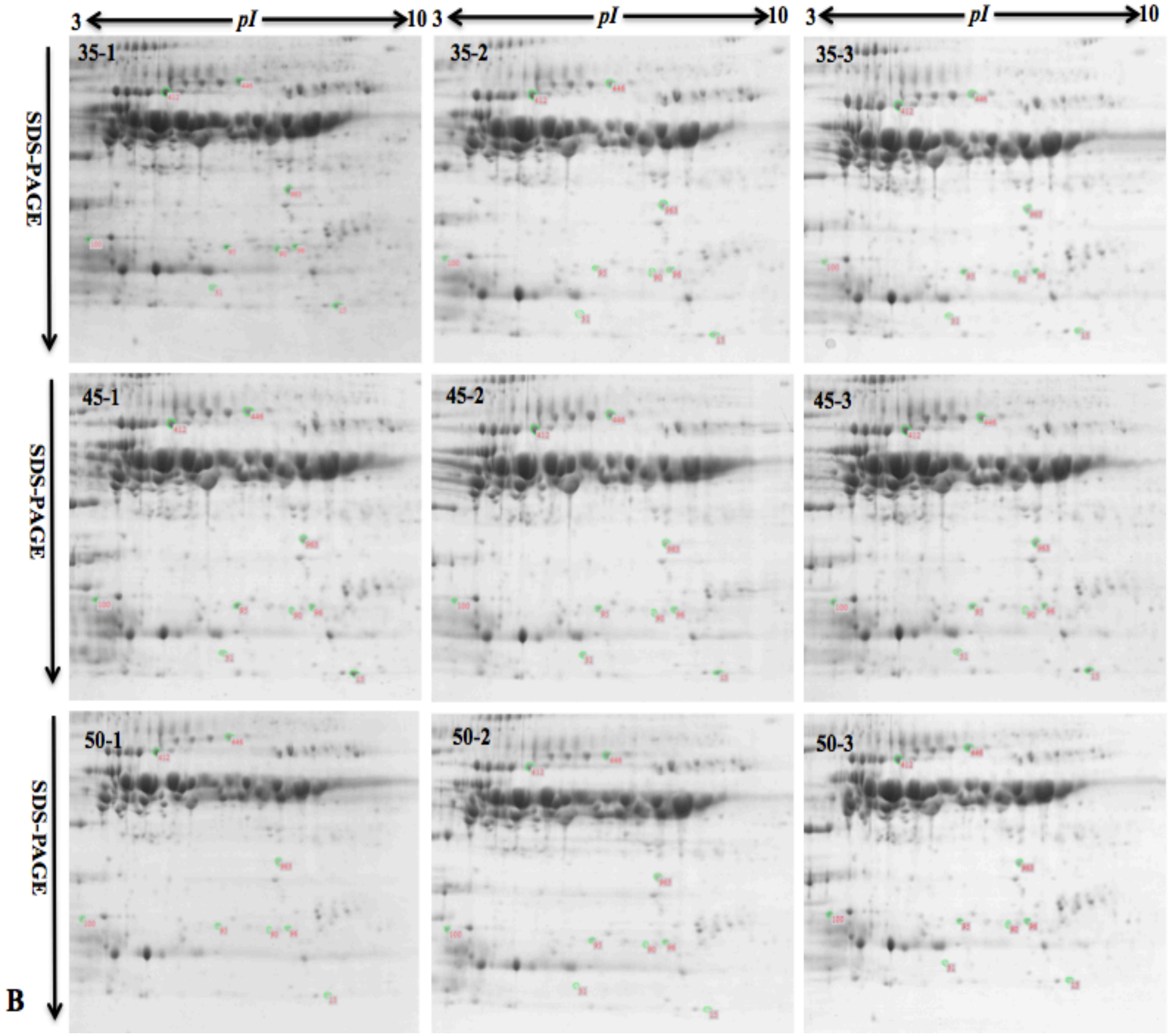
3
pI
10
35-1
35-2
35-3
45-1
45-2
45-3
50-1
50-2
50-3
SDS-PAGE
SDS-PAGE
SDS-PAGE
B
