# Supplementary material for: Proteomic and Physiological Analysis of the Response of Oat (Avena sativa) Seeds to Heat Stress under Different Moisture Conditions
Source: Front Plant Sci. 2016 Jun 22;7:896. doi: 10.3389/fpls.2016.00896 (PMC4916207; doi:10.3389/fpls.2016.00896)

Supplementary Figure 2. Peptide sequences of identified proteins and MS/MS fingerprint spectrum of each peptide

10% moisture content

Spot 33, Protein name: Avenin； Protein ID: Q09072

Peptide sequence: DFPITWPWK

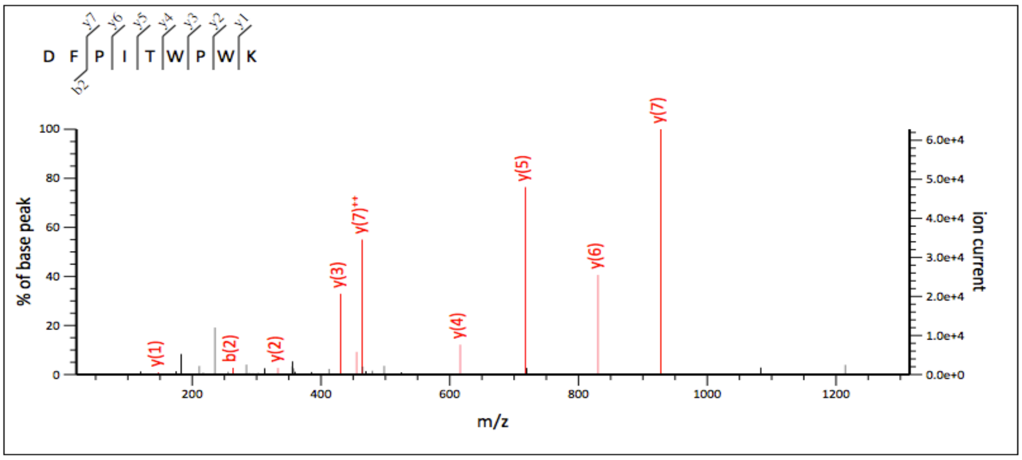

Peptide sequence: NECCQLLGQMPSECR

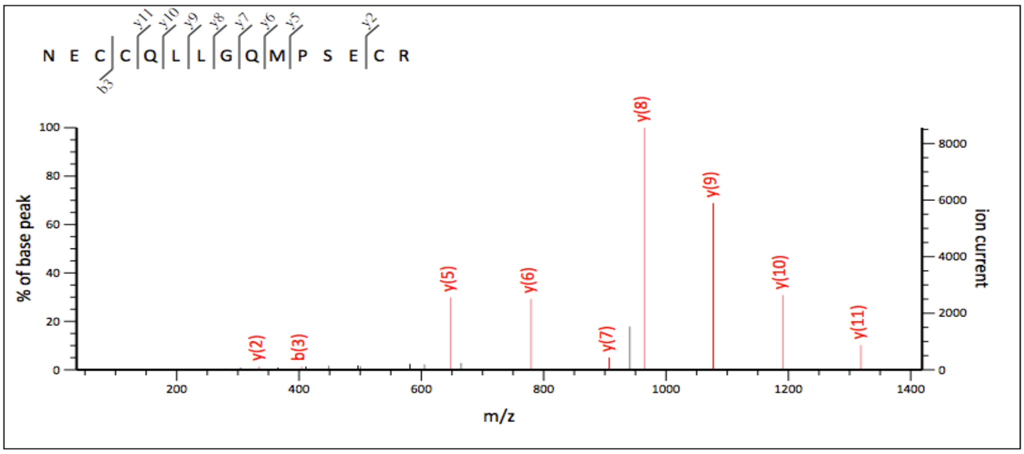

Spot 46, Protein name: 14 kDa zinc-binding protein； Protein ID: P42856

Peptide sequence: EIPSTVVYEDEKVLAFR

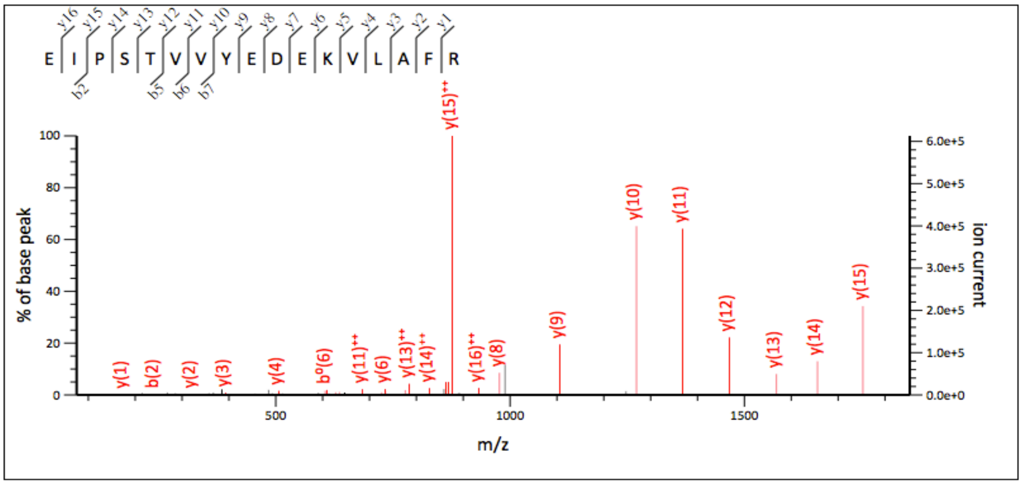

Peptide sequence: VVAKQEGLEDGYR

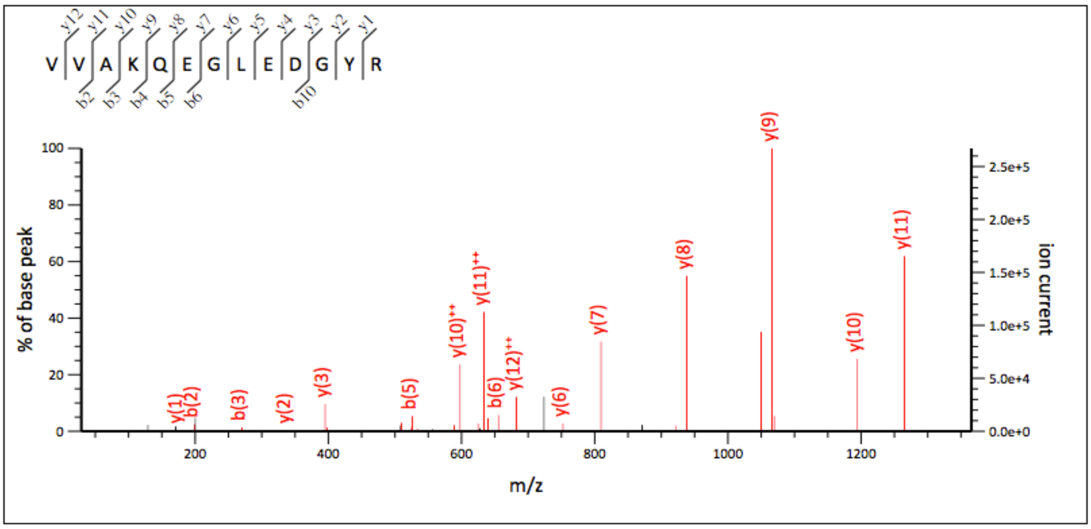

Spot 51, Protein name: ADP-ribosylation factor 1； Protein ID: P51821

Peptide sequence: ILMVGLDAAGK

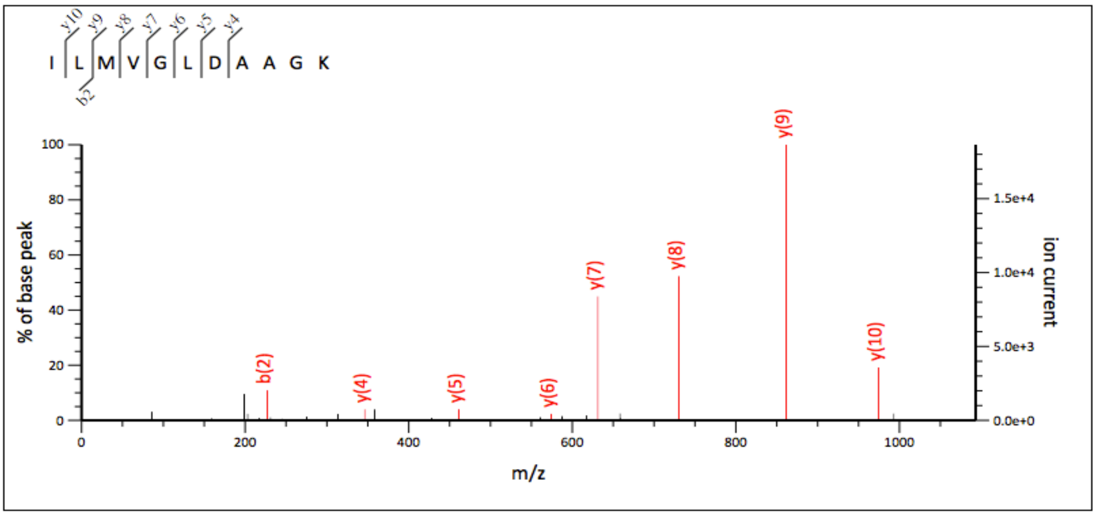

Peptide sequence: NISFTVWDVGGQDK

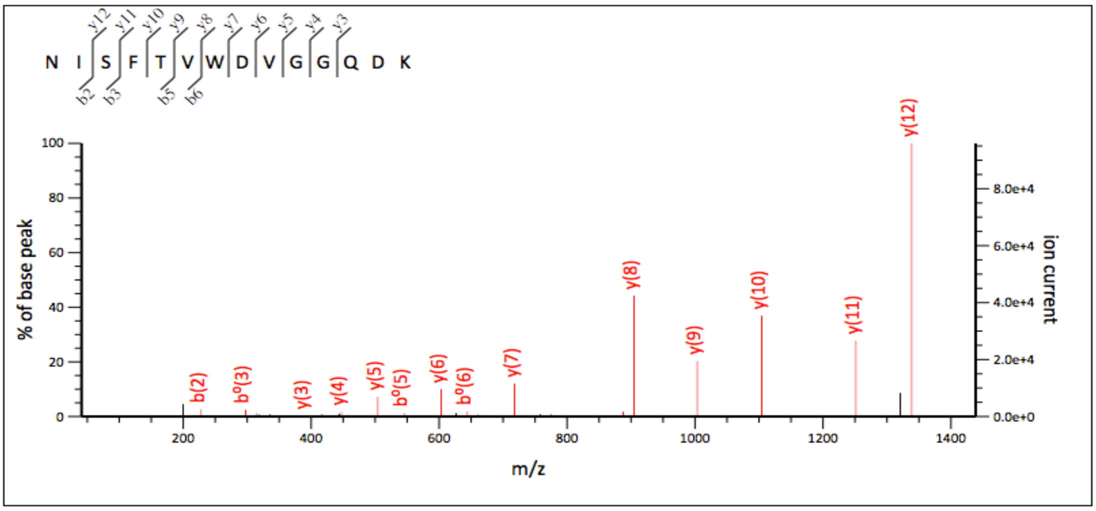

Peptide sequence: HYFQNTQGLIFVVDSNDR

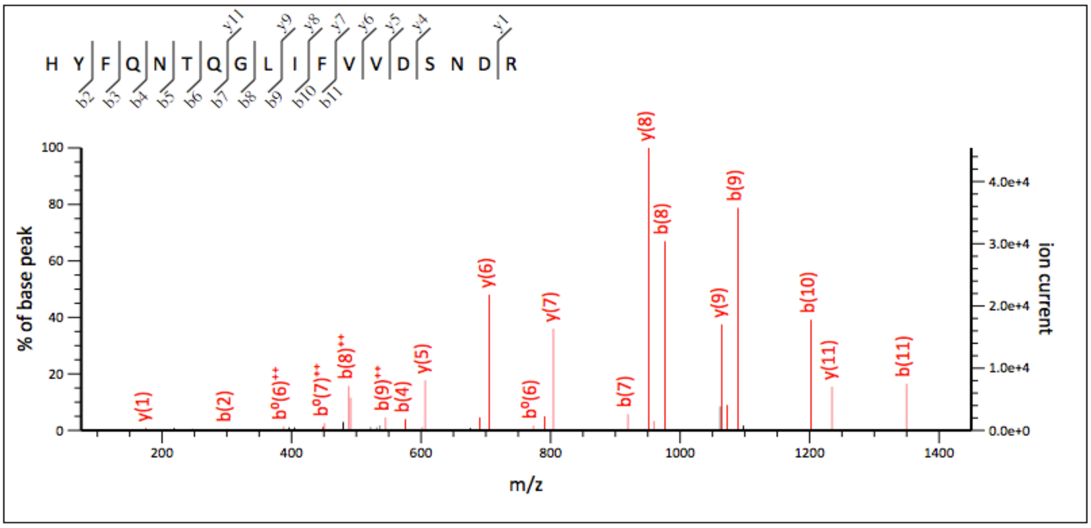

Spot 71, Protein name: 17.9 kDa class II heat shock protein； Protein ID: P46516

Peptide sequence: AMAATPADVK

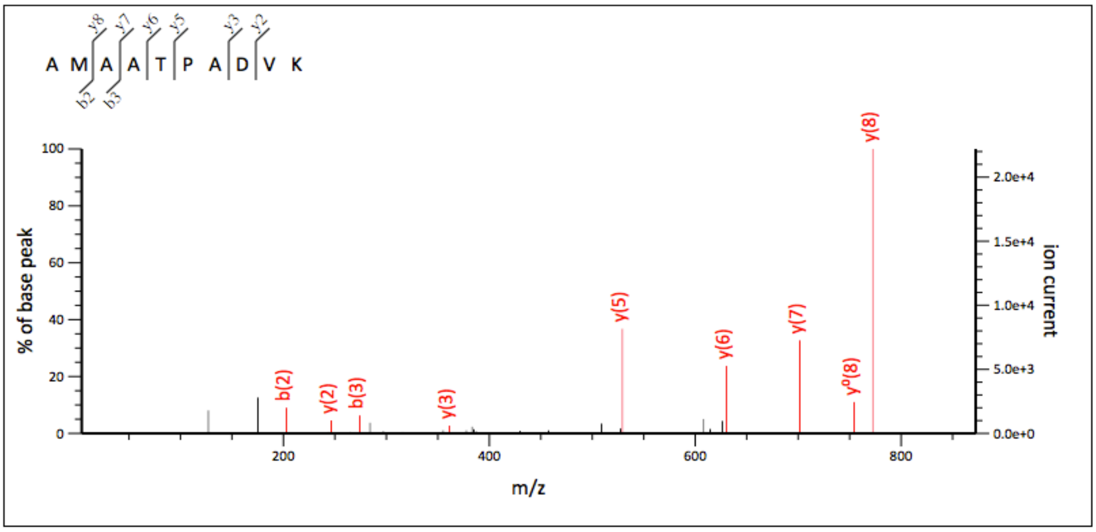

Peptide sequence: LPPPEPK

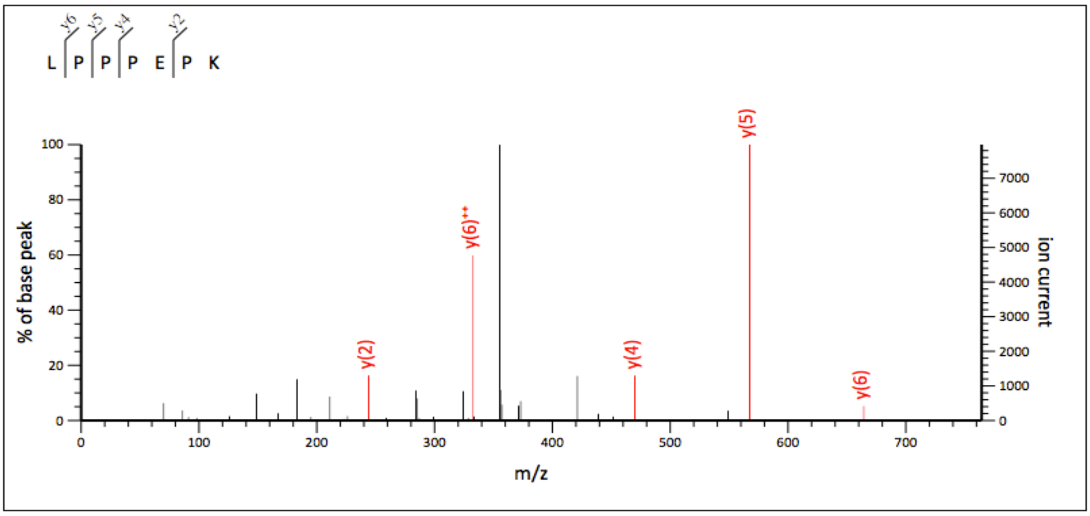

Spot 78, Protein name: 18.3 kDa class I heat shock protein; Protein ID: Q05832

Peptide sequence: ETPEAHVFKADLPGVK

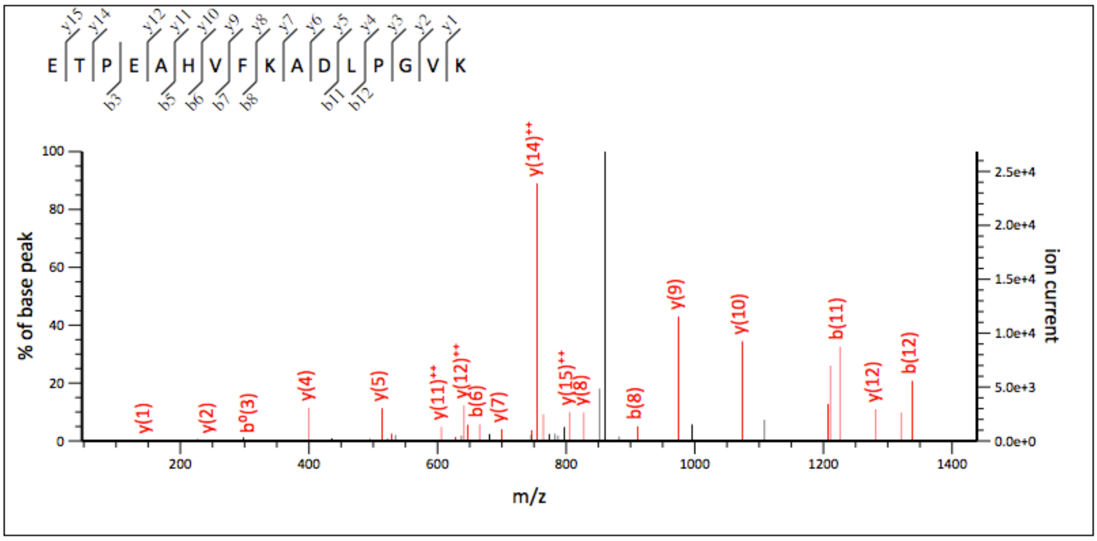

Peptide sequence: ADLPGVKKEEVK

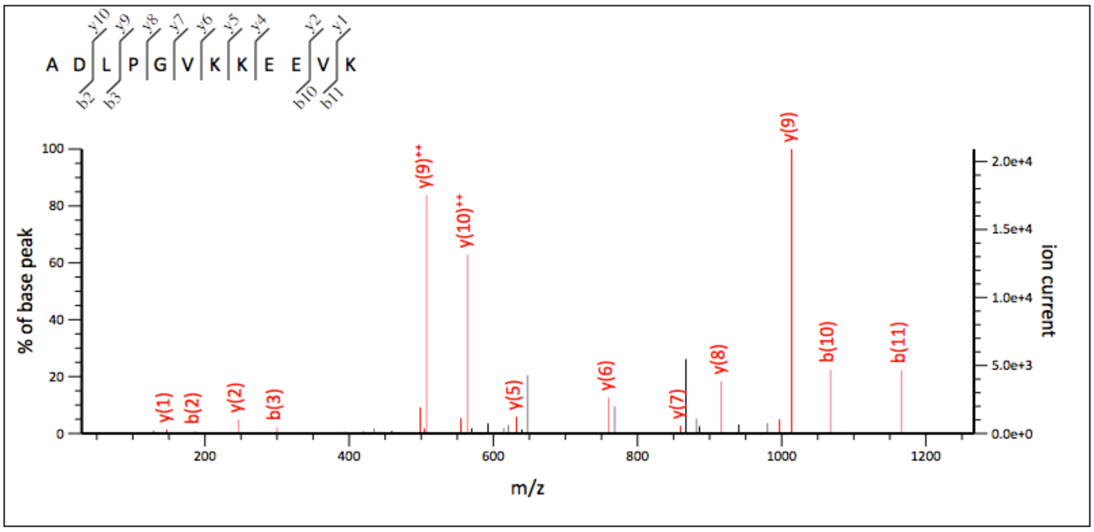

Spot 92, Protein name: Late embryogenesis abundant protein B19.3; Protein ID: Q02400

Peptide sequence: EGETVVPGGTGGK

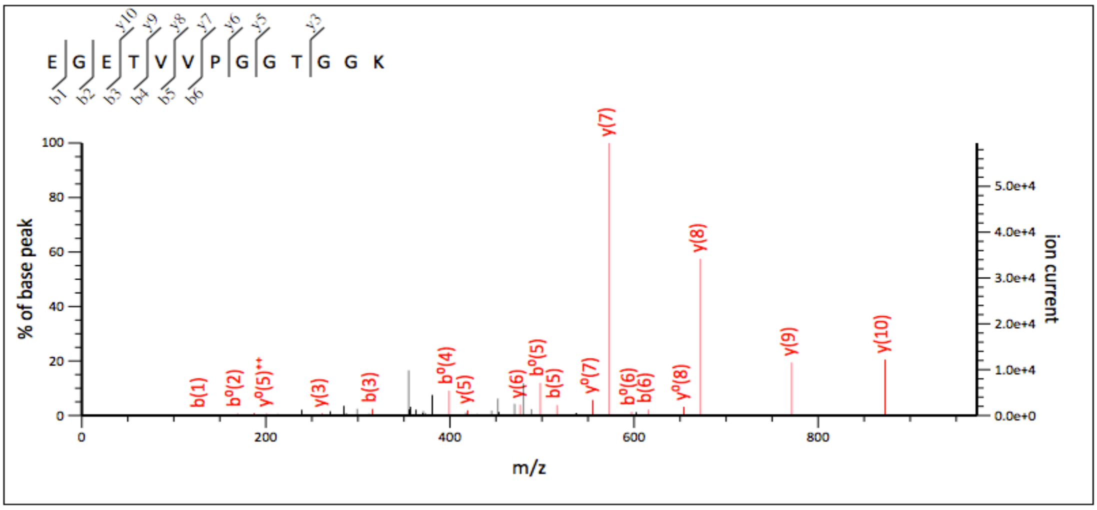

Peptide sequence: KEQLGEEGYR

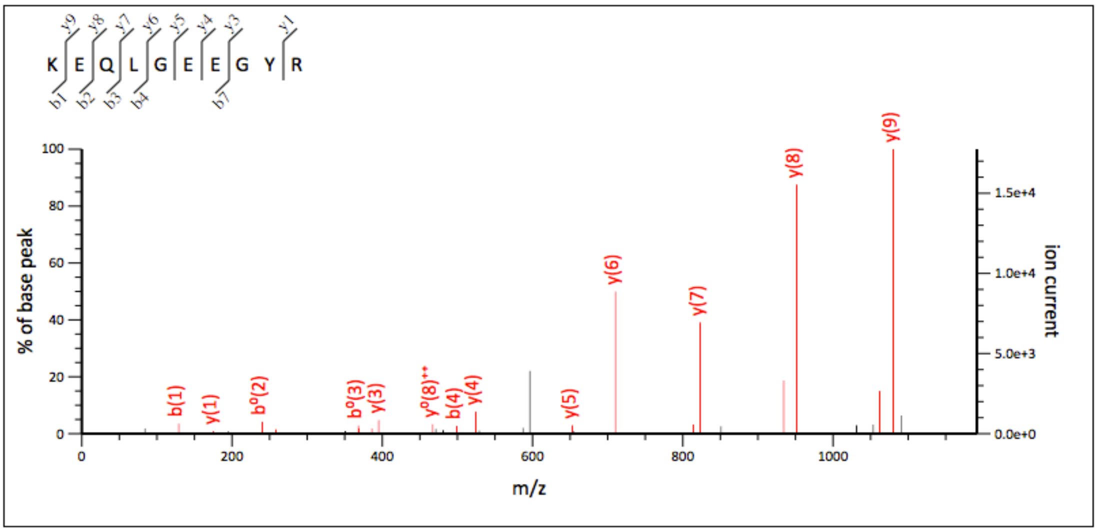

Peptide sequence: EGIDIDESKFK

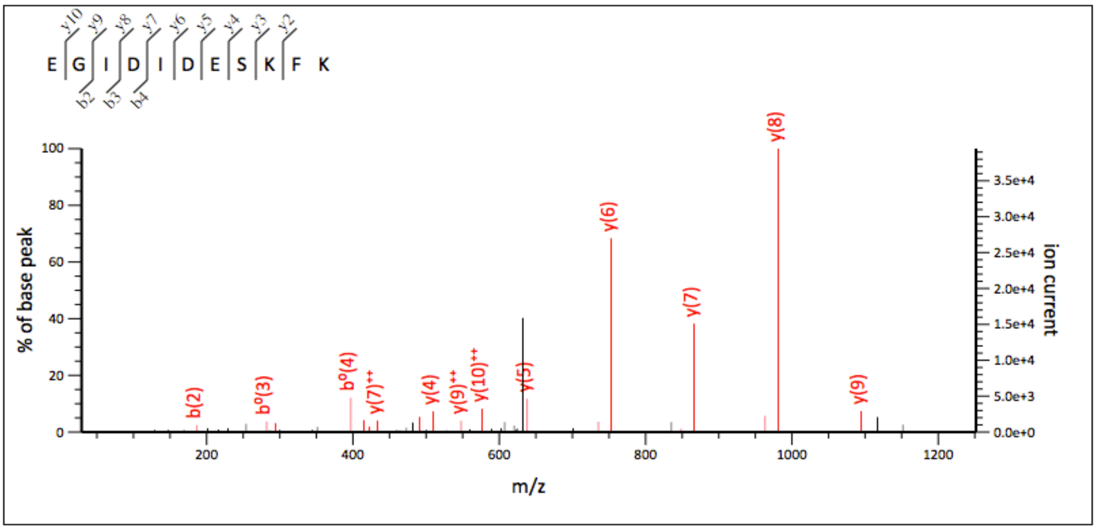

Spot 99, Protein name: 16.9 kDa class I heat shock protein 1； Protein ID: P12810

Peptide sequence: VDWKETPEAHVFK

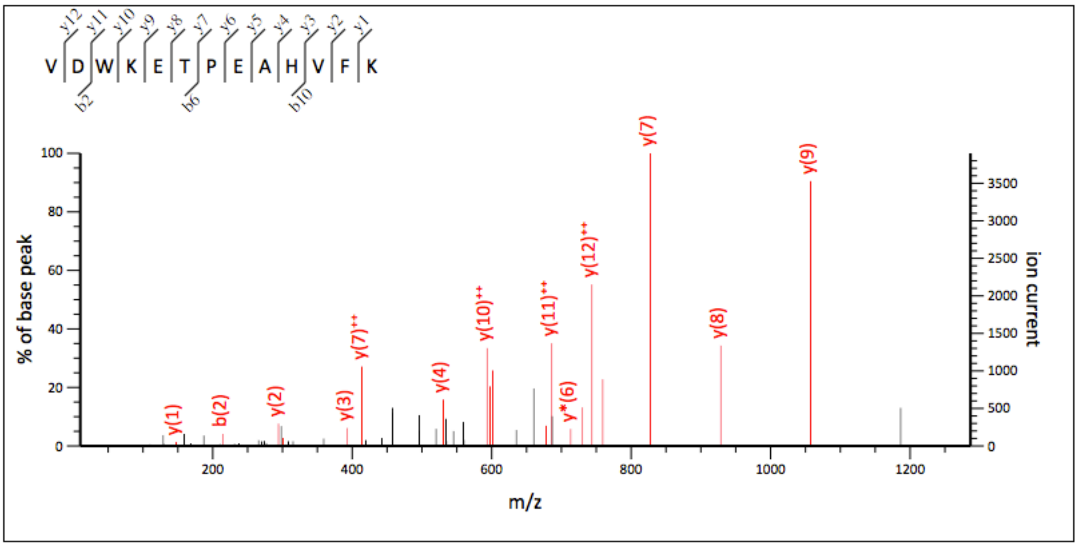

Peptide sequence: KEEVKVEVEDGNVLVVSGER

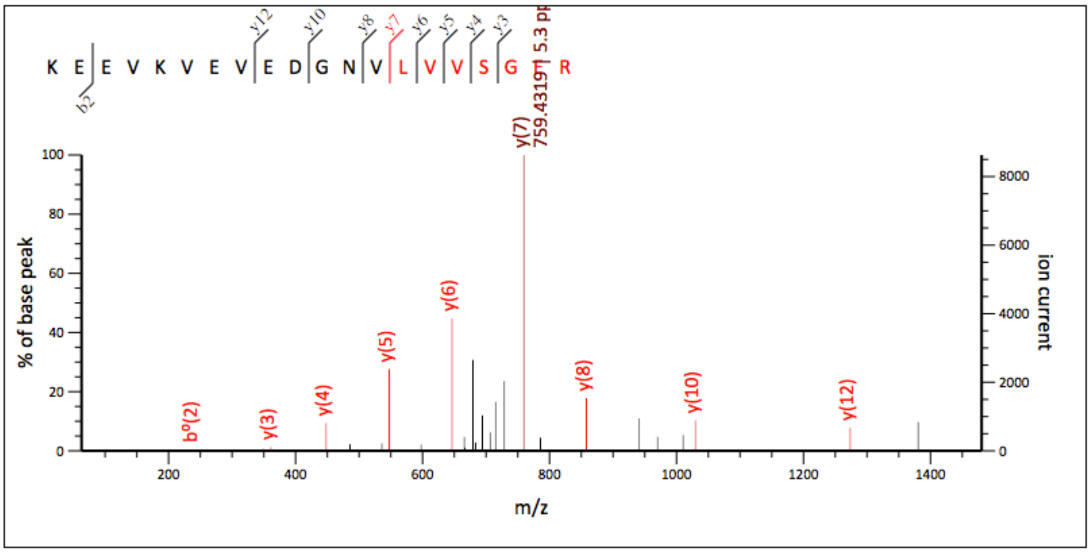

Peptide sequence: VEVEDGNVLVVSGER

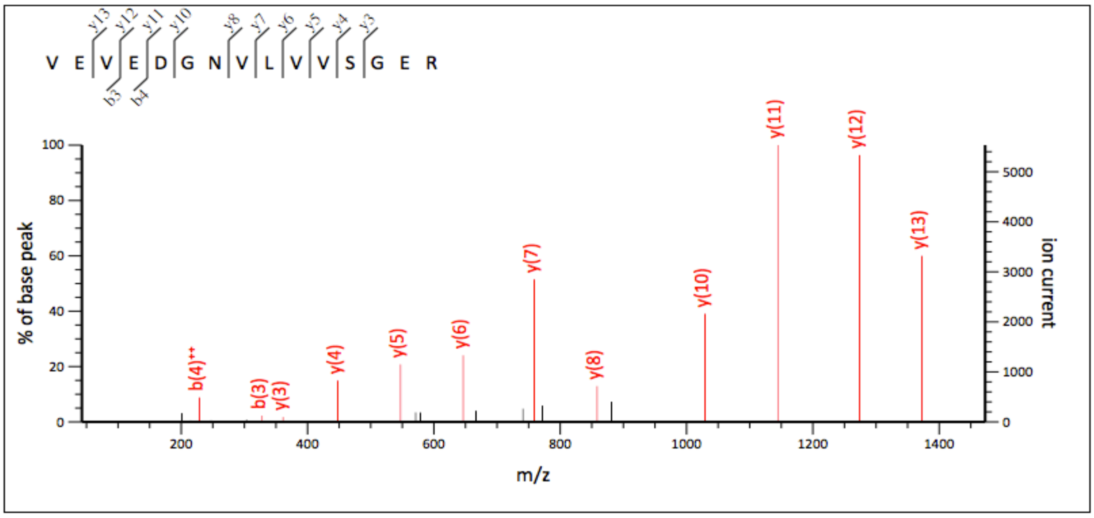

Spot 106, Protein name: Eukaryotic translation initiation factor 1A； Protein ID: P47815

Peptide sequence: ELVFKEDGQEYAQVTR

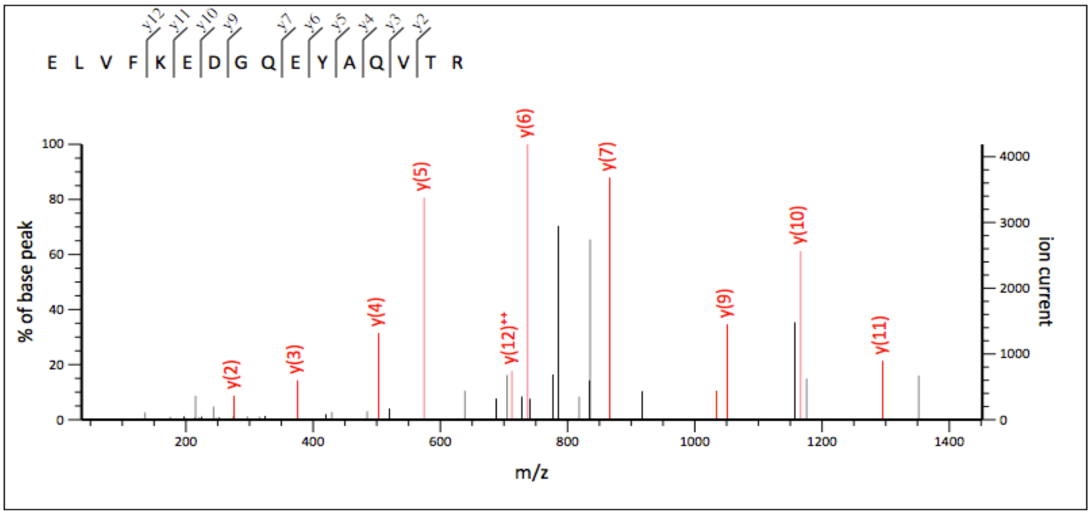

Peptide sequence: CEAICVDGTKR

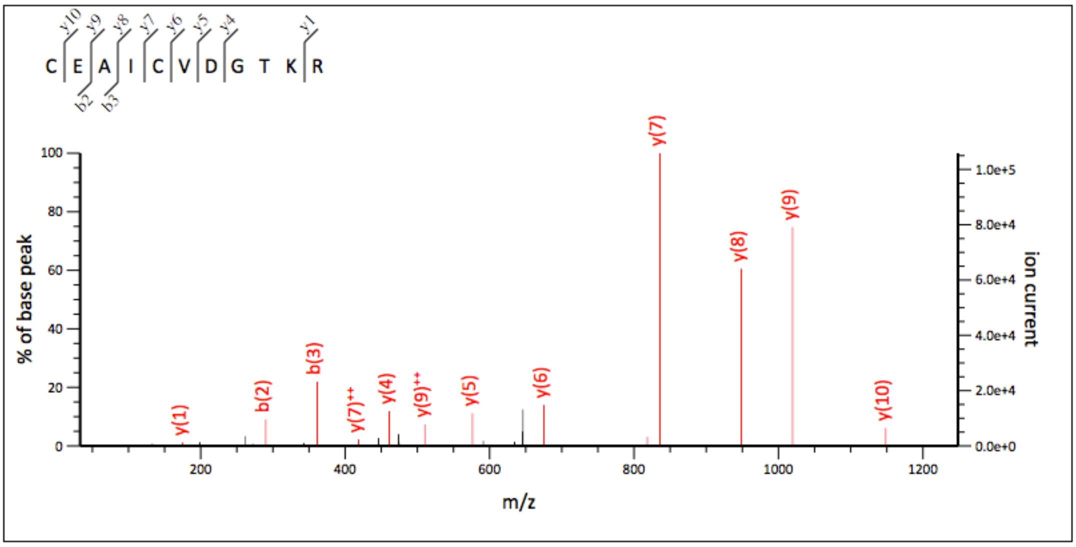

Peptide sequence: DYQDDKADVILK

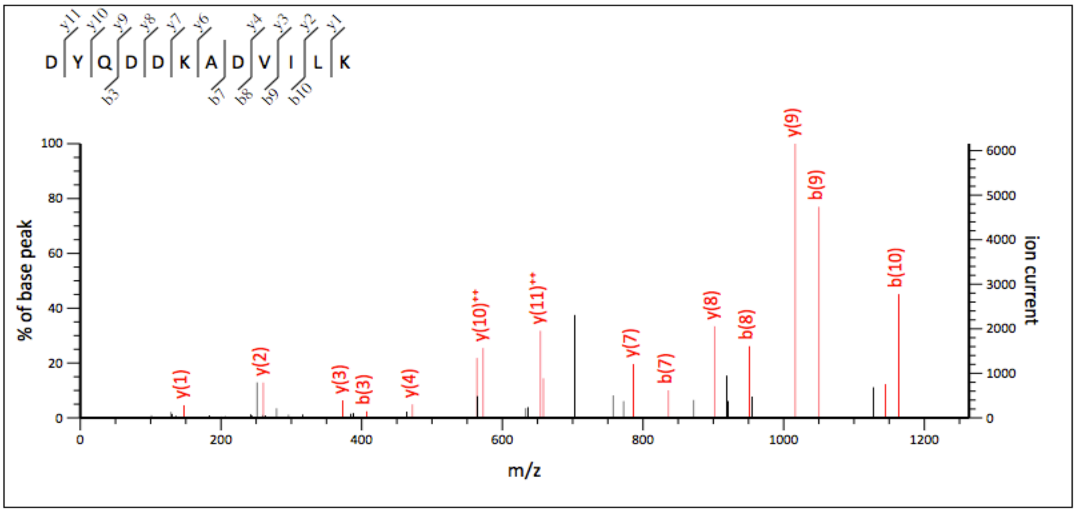

Spot 362, Protein name: 12S seed storage globulin 2; Protein ID: P14812

Peptide sequence: LQAFEPLR

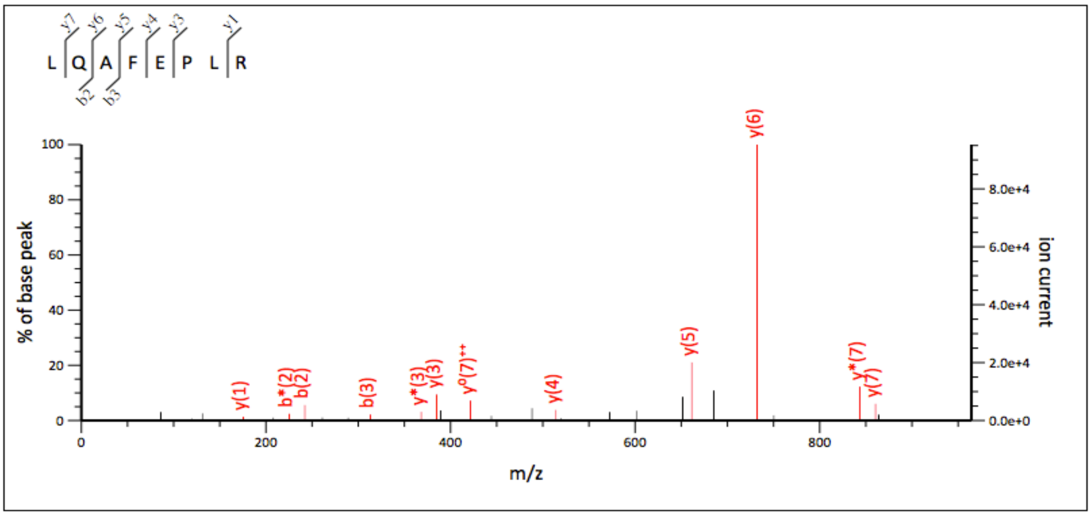

Peptide sequence: CAGVSVIR

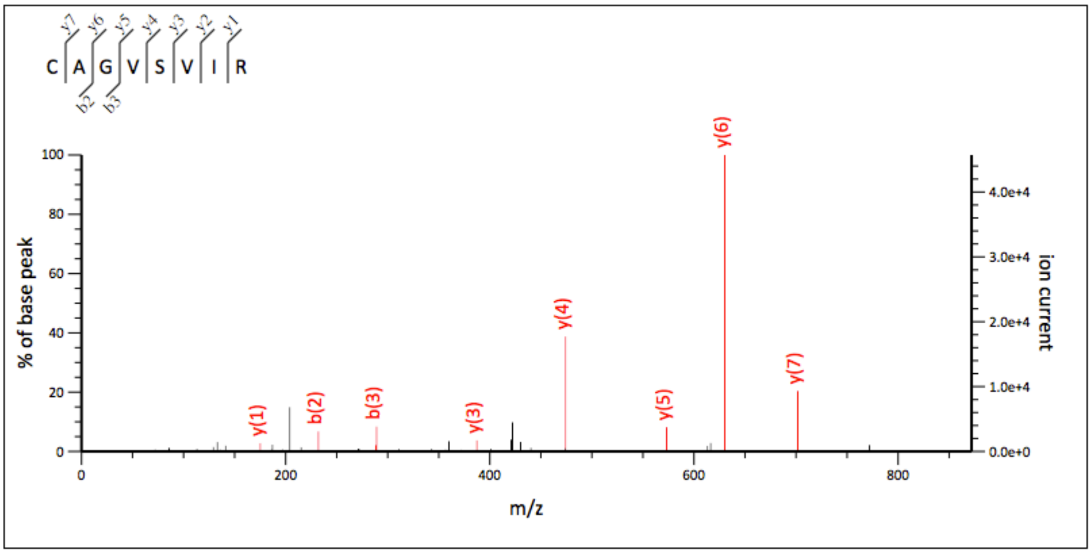

Peptide sequence: VIEPQGLLLPQYHNAPGLVYILQGR

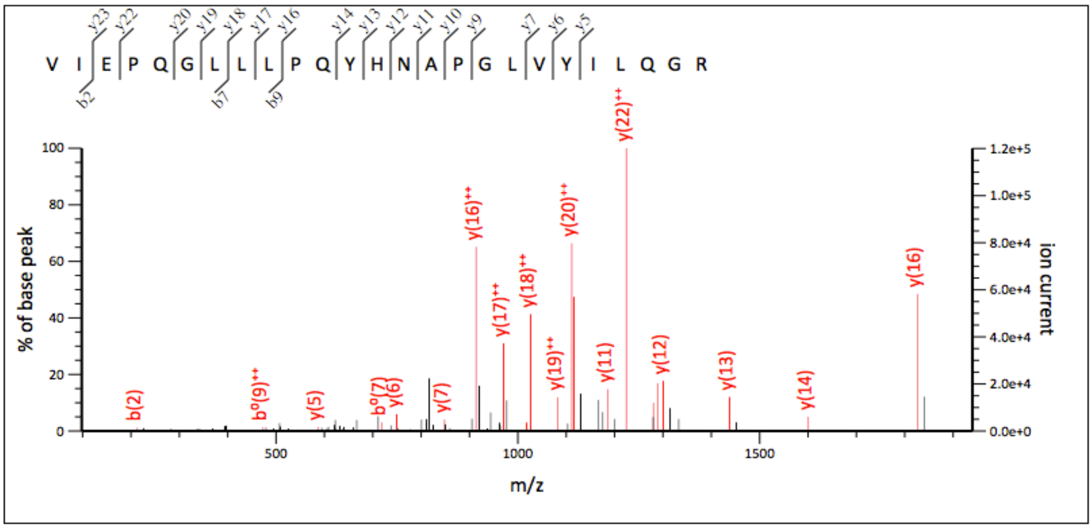

Peptide sequence: EFLLAGNNKR

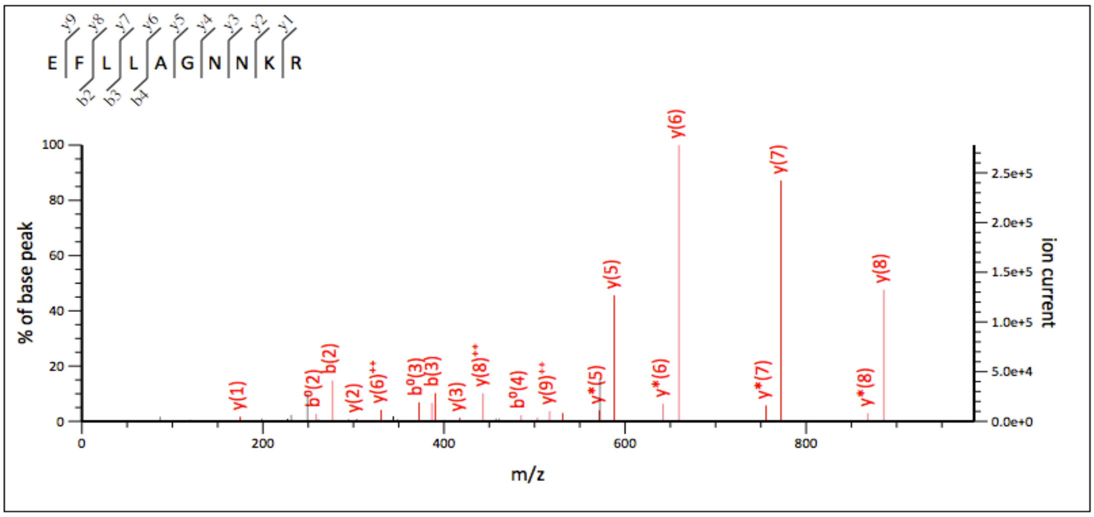

Peptide sequence: IQSQNDQRGEIIR

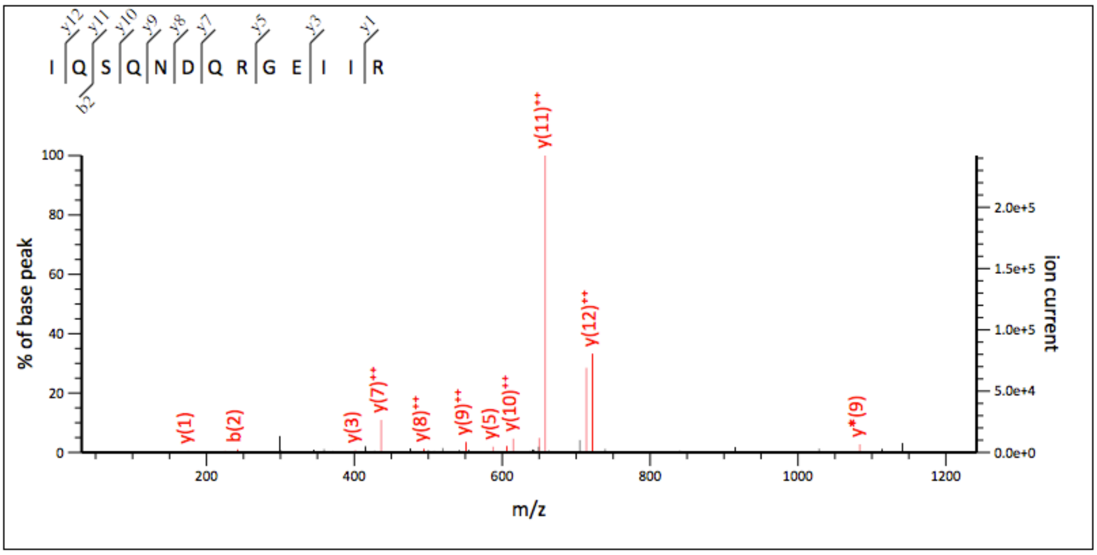

Peptide sequence: NFPTLNLVQMSATR

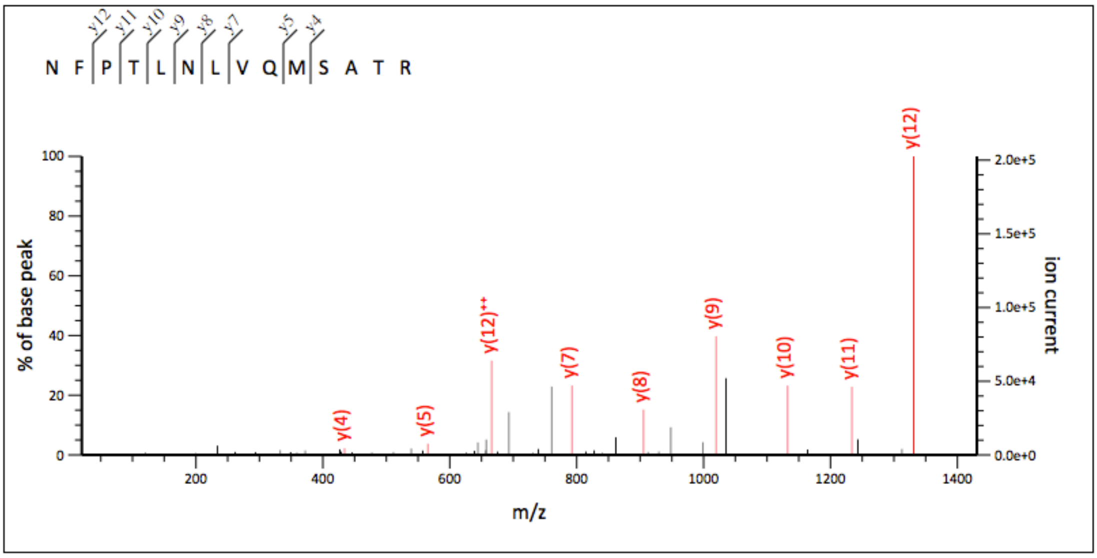

Peptide sequence: VQVVNNHGQTVFNDILR

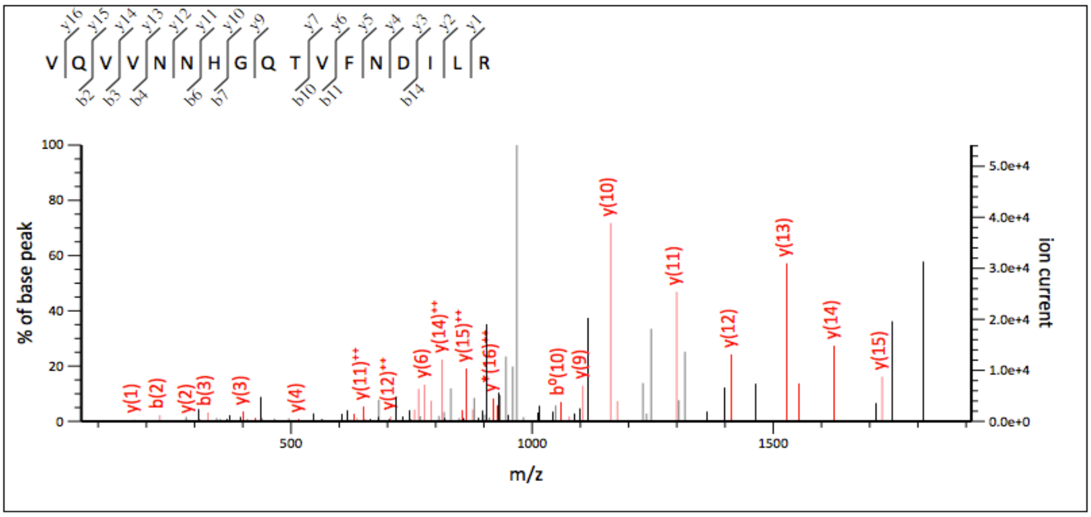

Peptide sequence: GQLLIIPQHYVVLK

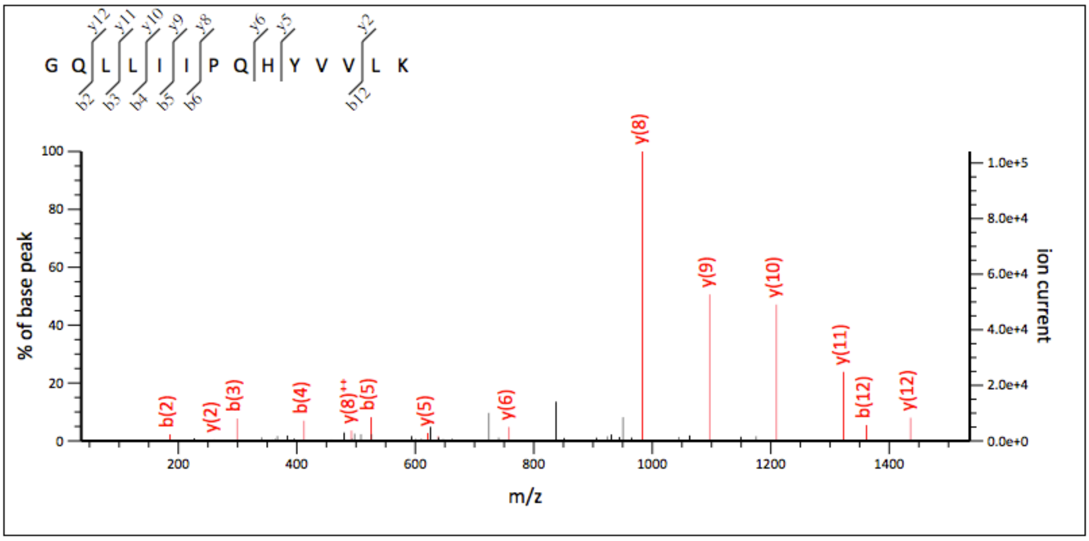

Peptide sequence: EGCQYISFK

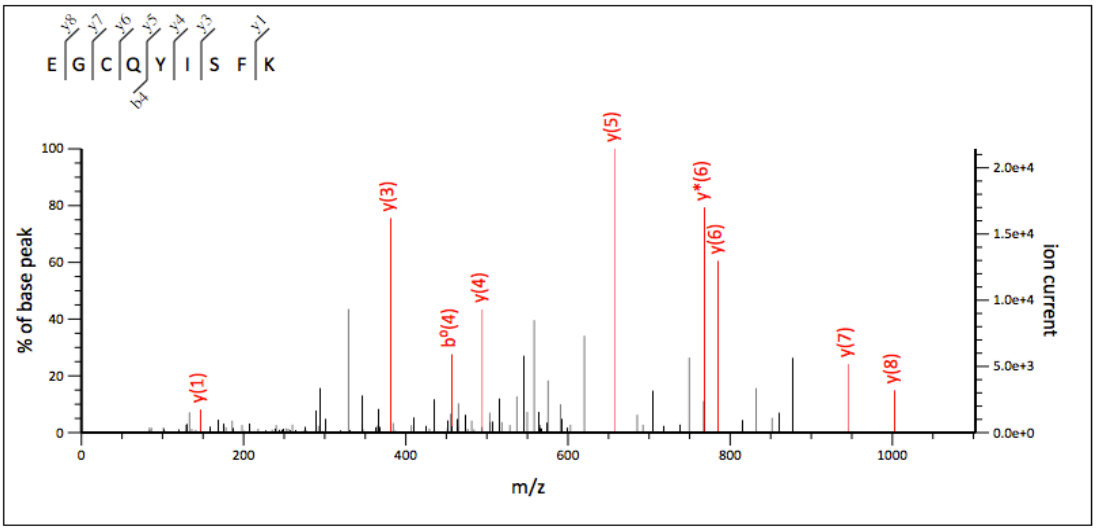

Peptide sequence: TNPNSMVSQIAGK

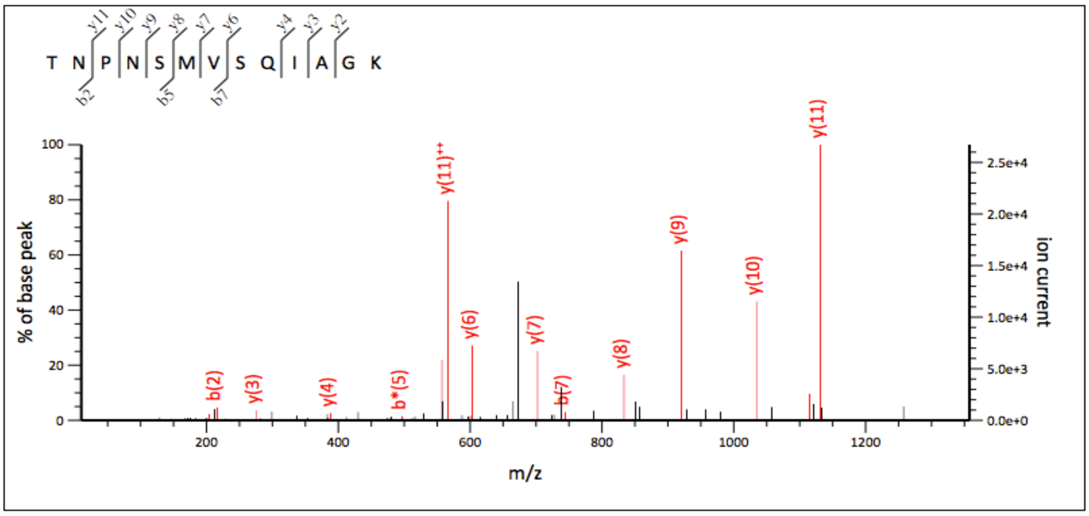

Peptide sequence: ALPVDVLANAYR

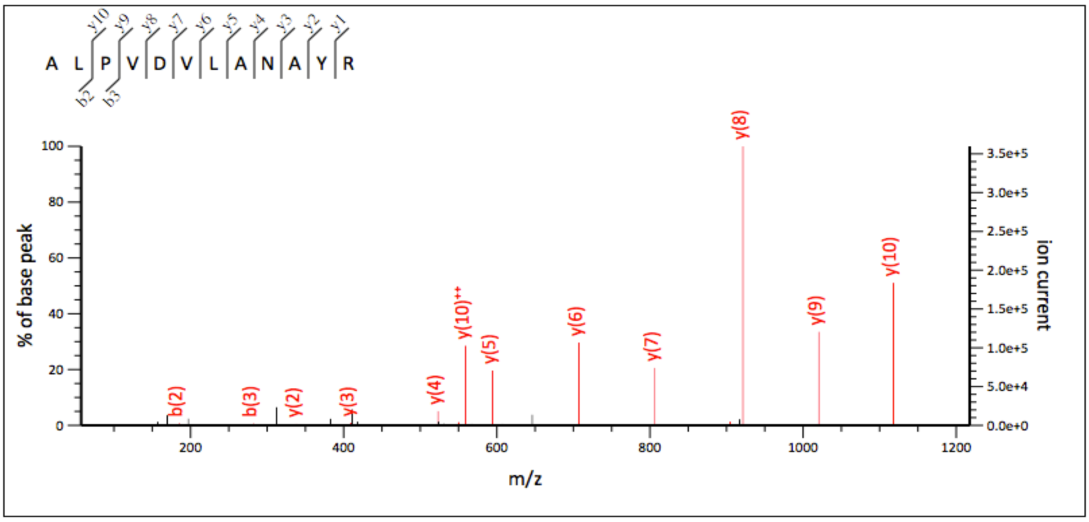

Peptide sequence: NNRGEEFDAFTPK

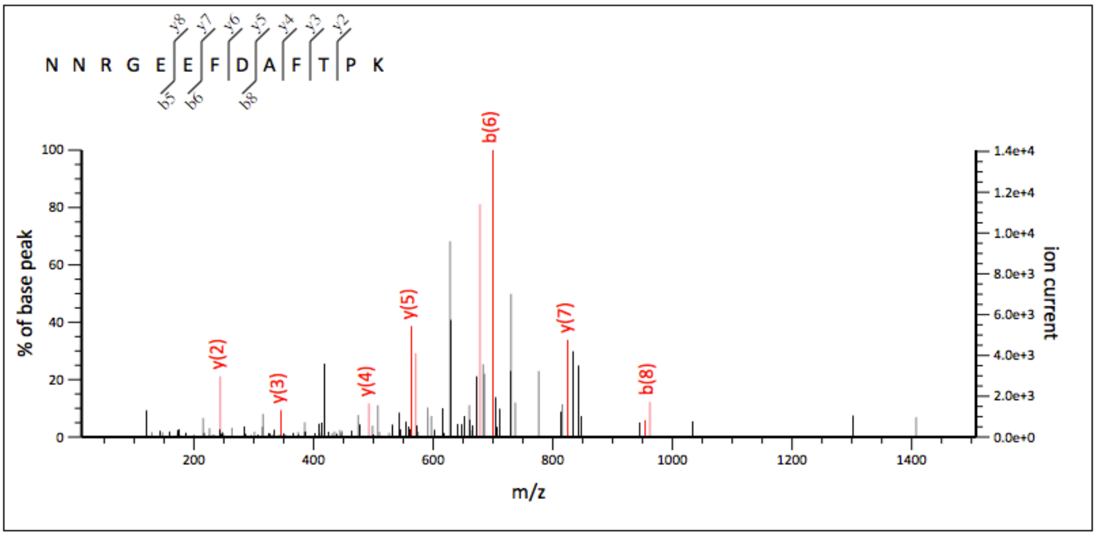

Spot 515, Protein name: EMB-1 protein； Protein ID: P17639

Peptide sequence: QGETVVPGGTGGK

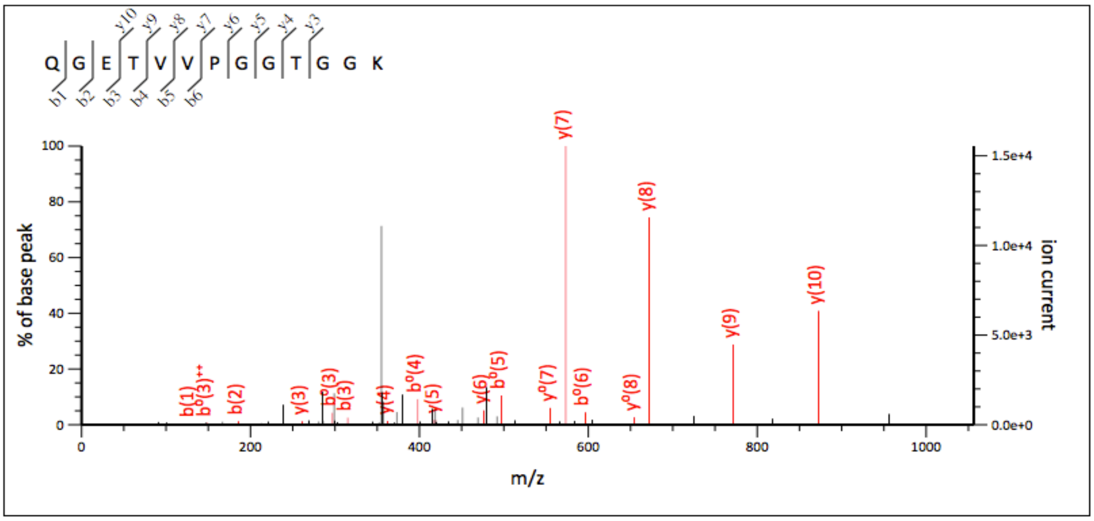

Spot 758, Protein name: Luminal-binding protein 2； Protein ID: P24067

Peptide sequence: NGHVEIIANDQGNR

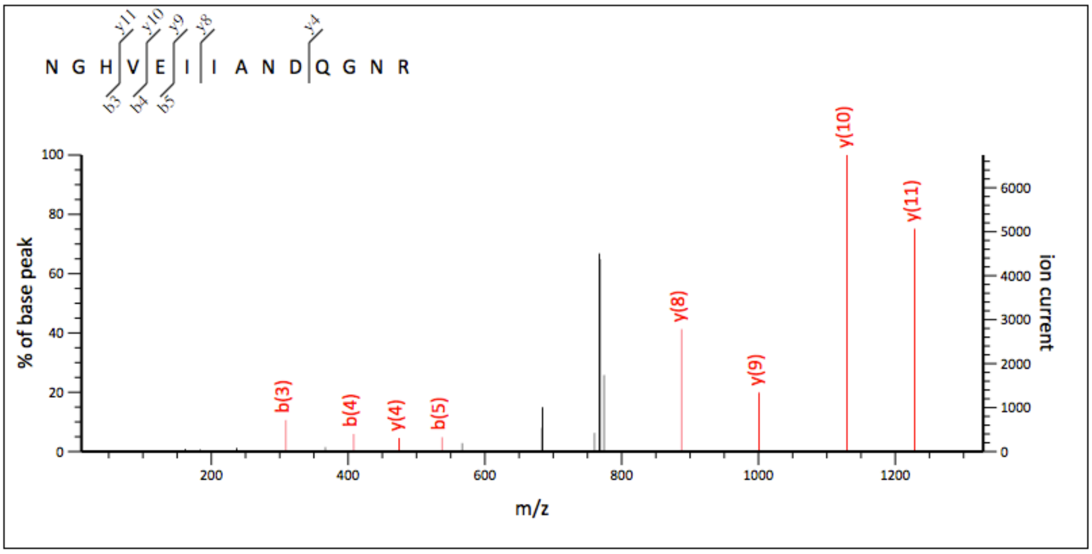

Peptide sequence: VFSPEEISAMILGK

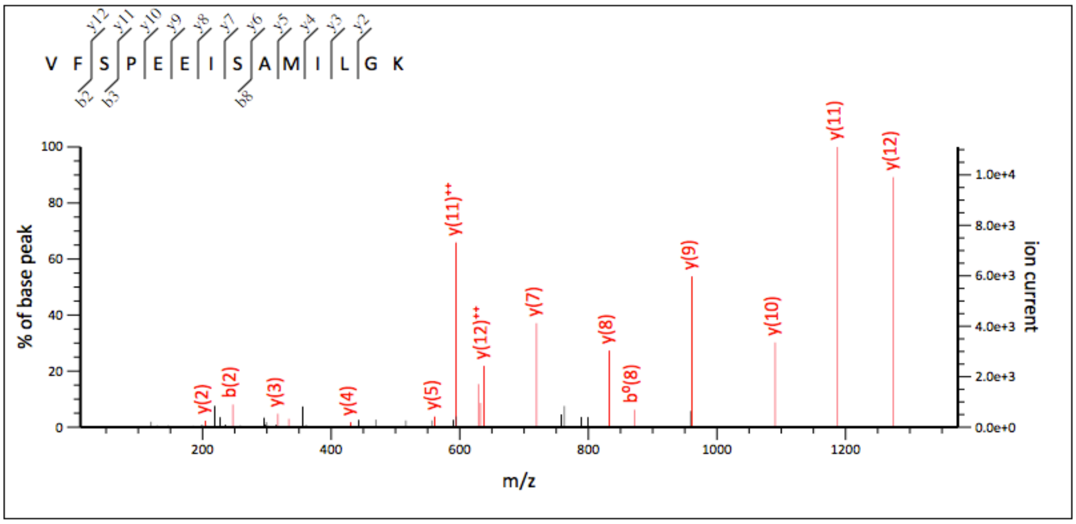

Peptide sequence: INDAVVTVPAYFNDAQR

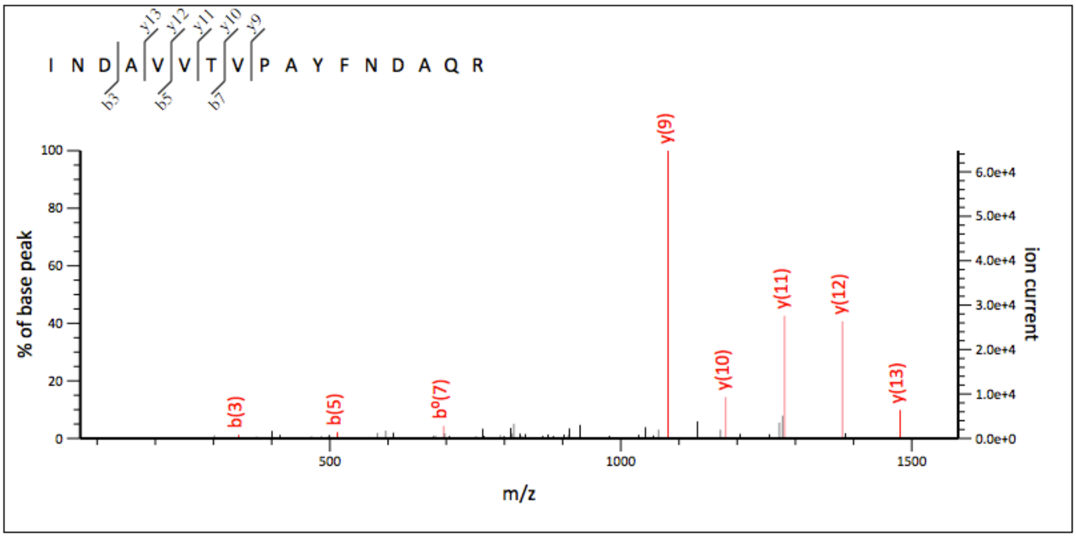

Peptide sequence: IINEPTAAAIAYGLDK

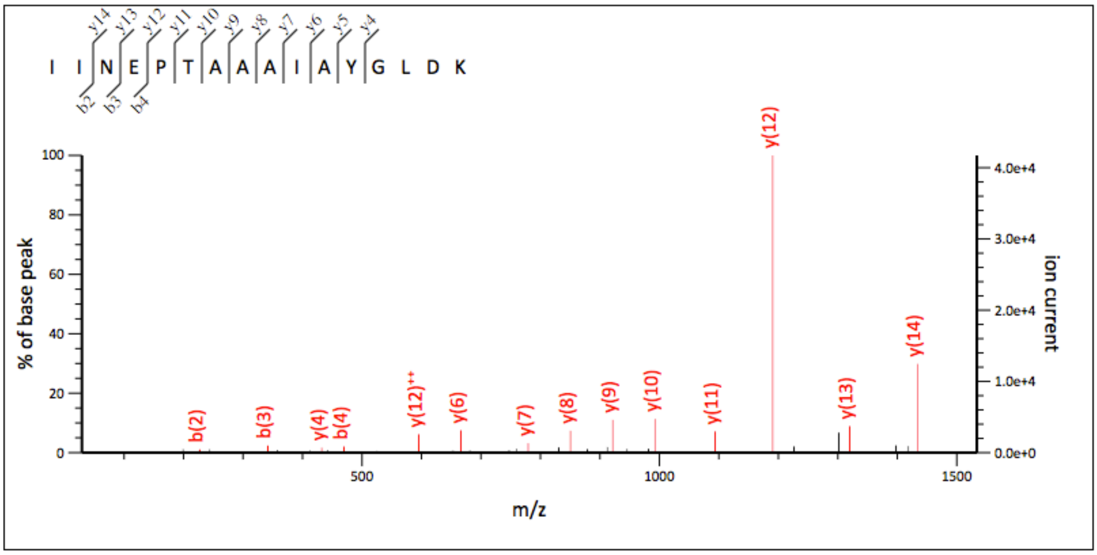

Peptide sequence: VEIESLFDGTDFSEPLTR

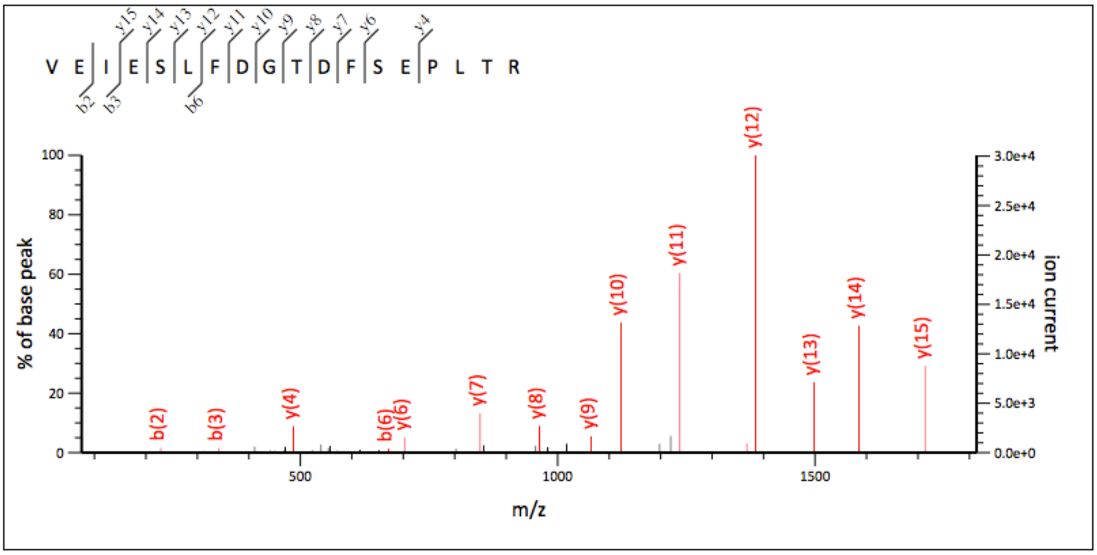

Peptide sequence: SQIHEIVLVGGSTR

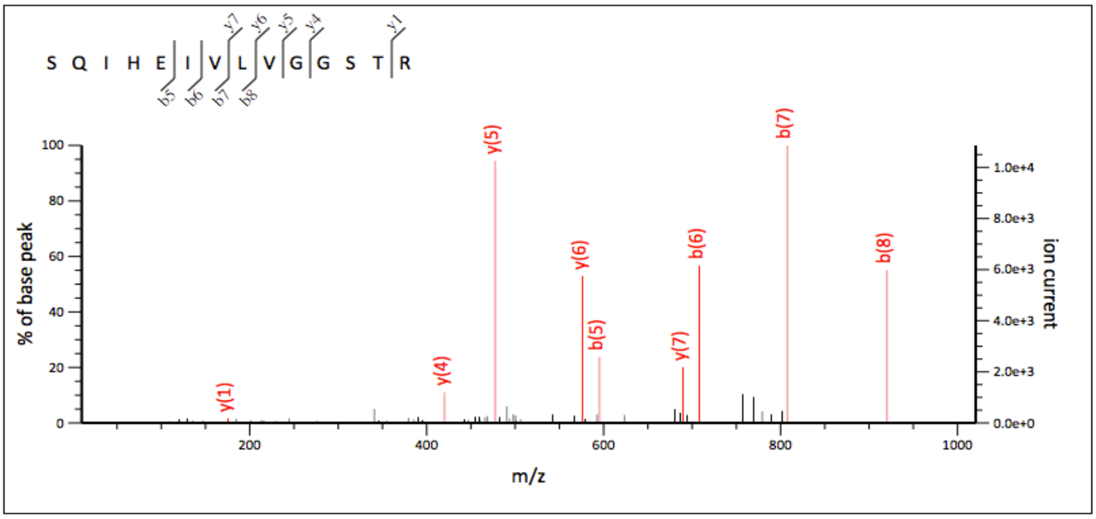

Peptide sequence: ITITNEK

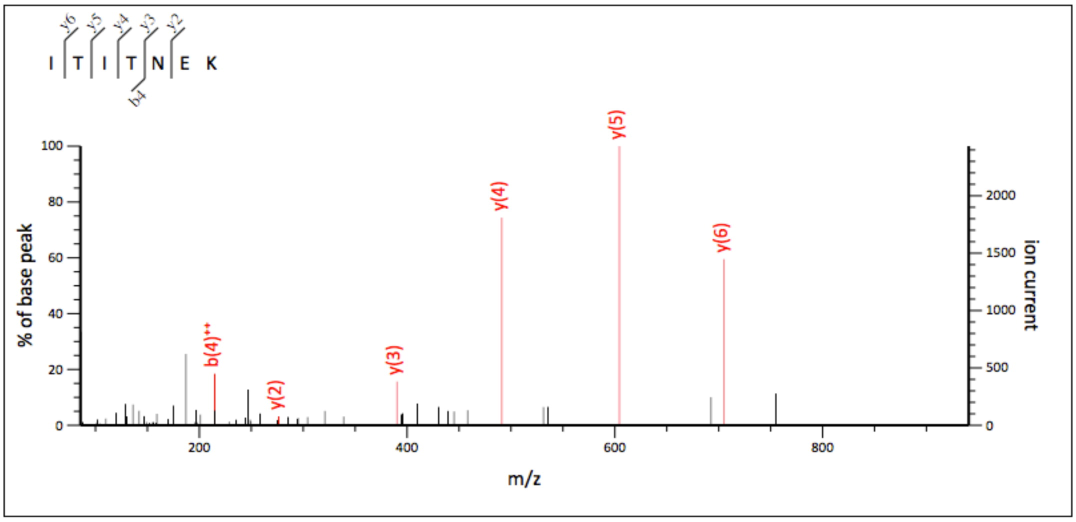

Peptide sequence: EAEEFAEEDKK

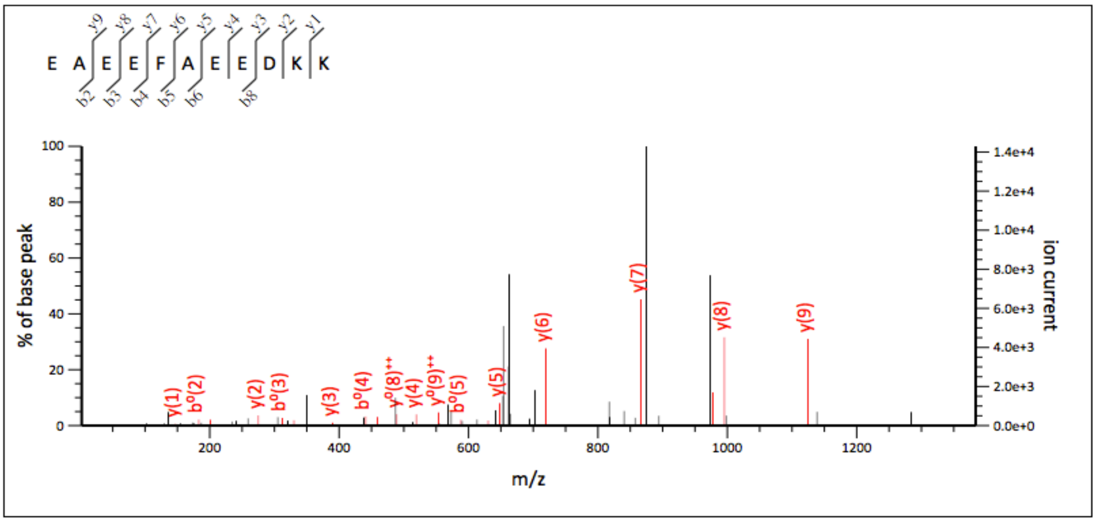

Spot 889, Protein name: ATP synthase subunit d, mitochondrial；   Protein ID: Q9FT52

Peptide sequence: VLVTDEAR

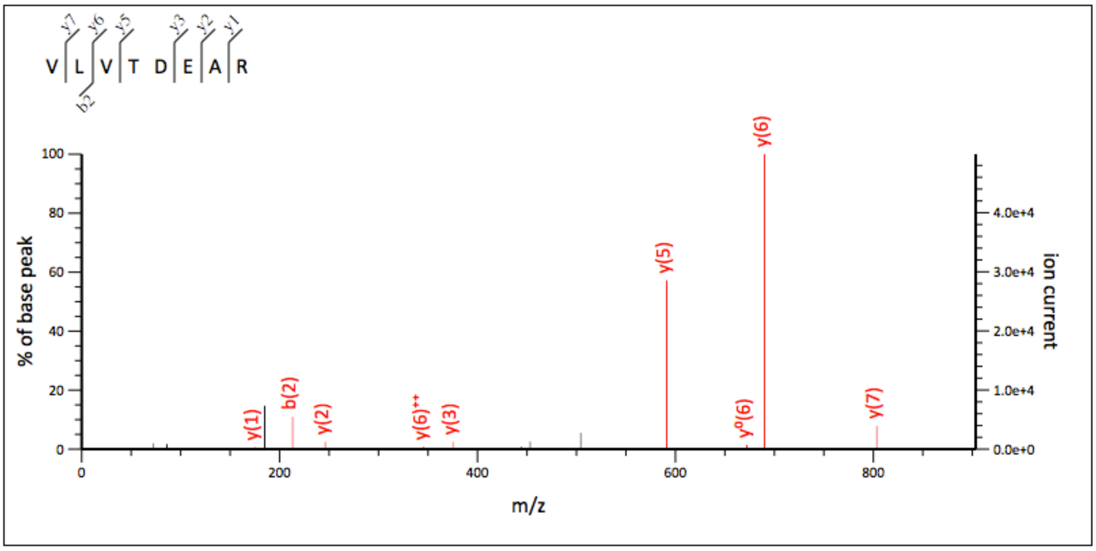

Peptide sequence: EAYDSIEIPK

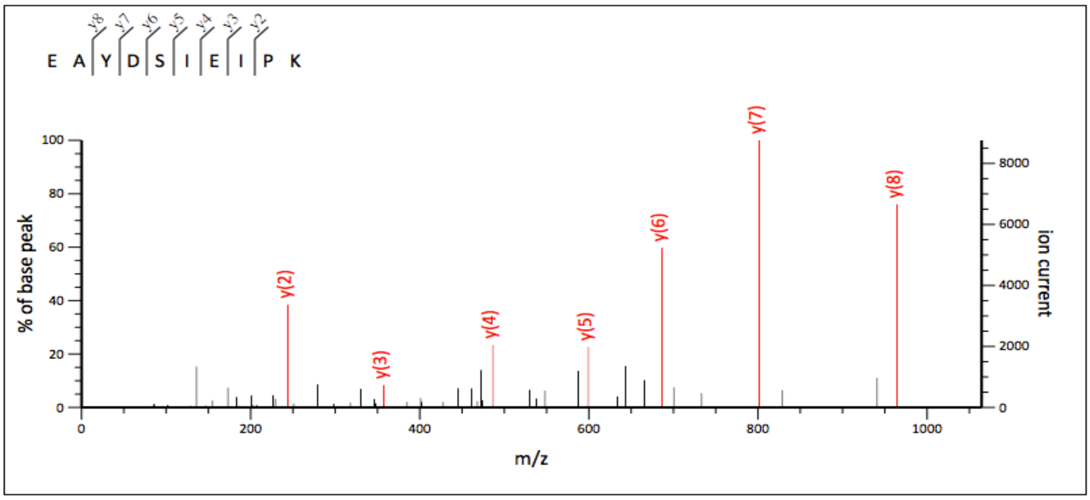

Peptide sequence: FDALLVELK

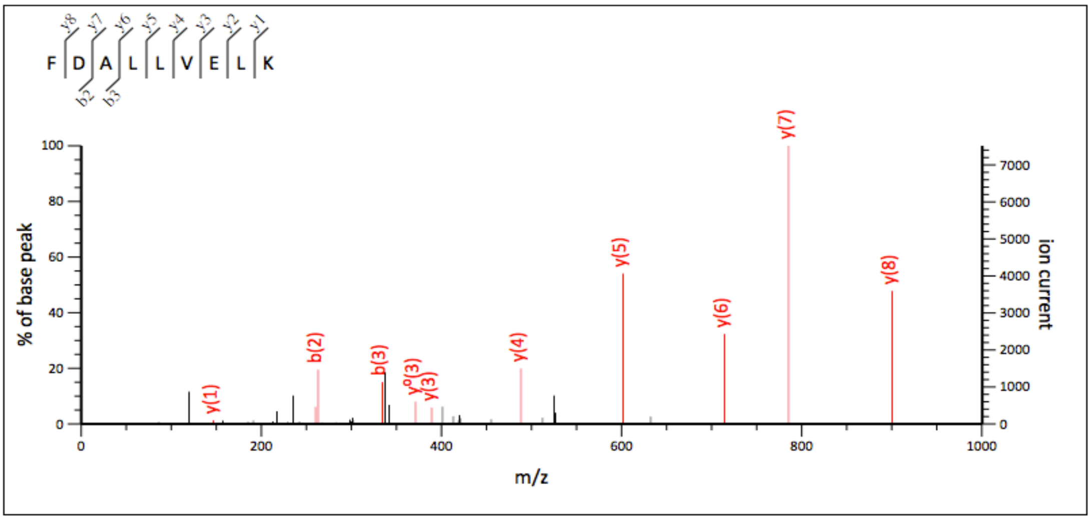

16% moisture content

Spot 15, Protein name: Em protein CS41； Protein ID: P22701

Peptide sequence: EGQTVVPGGTGGK

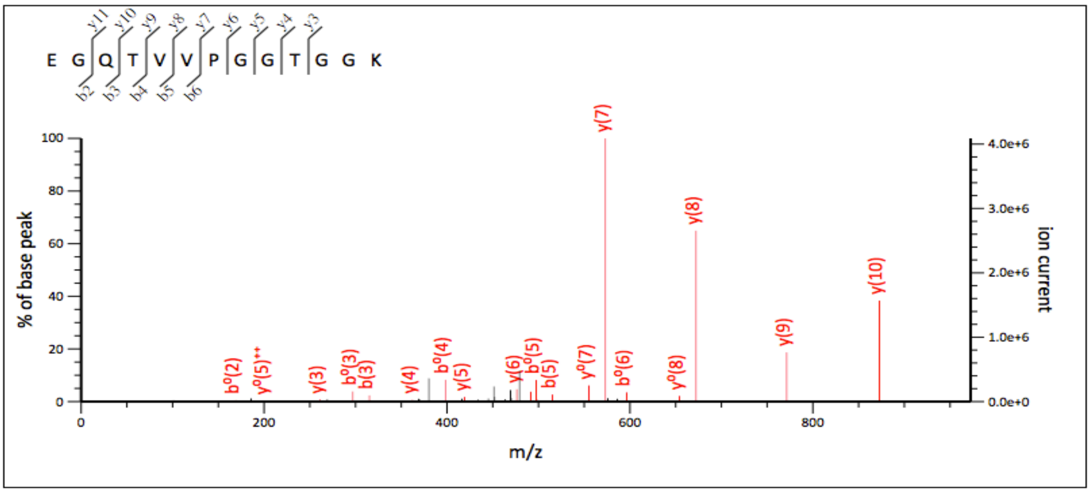

Peptide sequence: KGGLSTNDESGGER

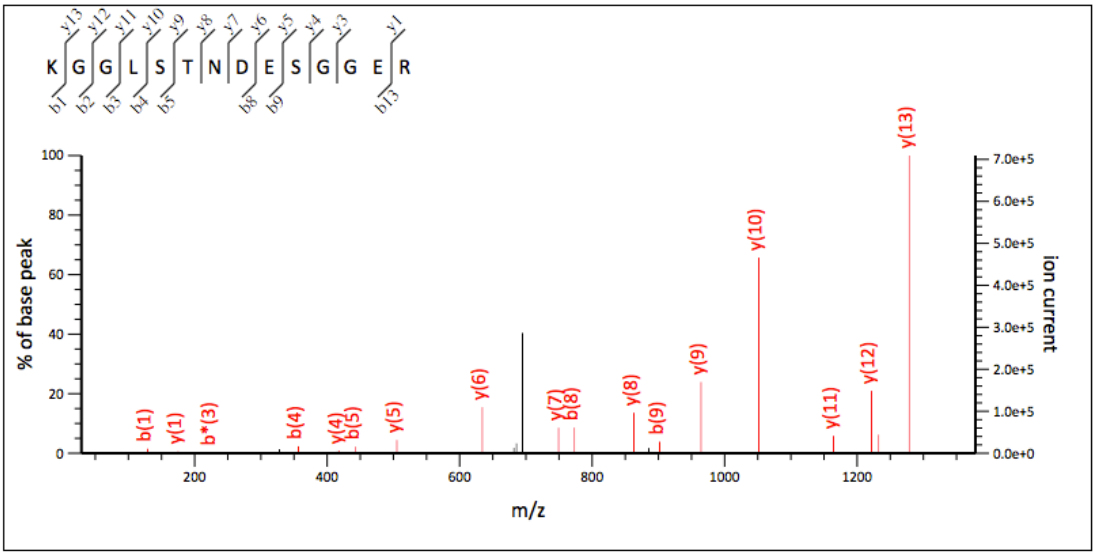

Peptide sequence: EGIDIDESK

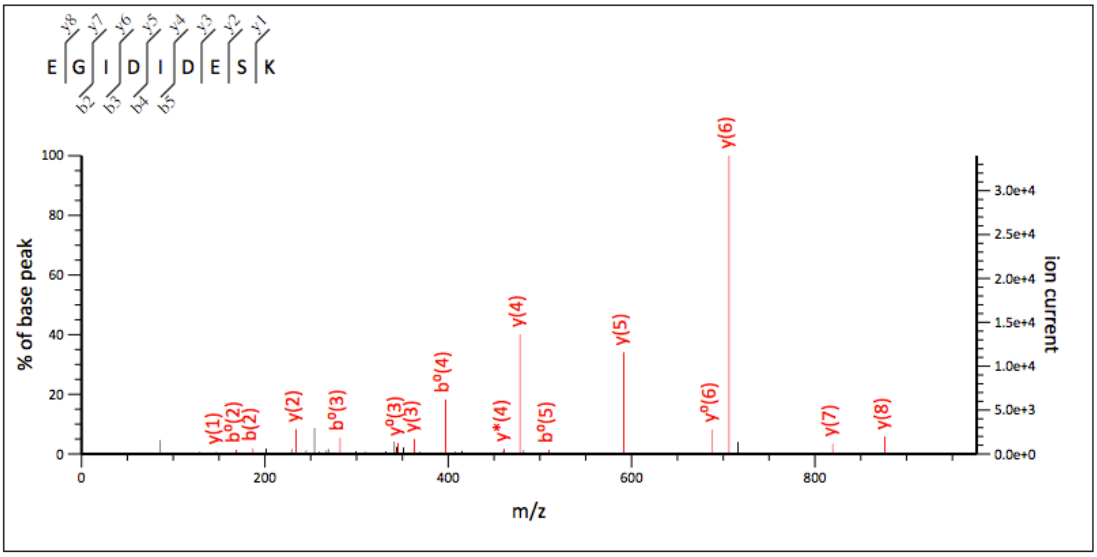

Spot 31(Spot 33), Protein name: Avenin；   Protein ID: Q09072

Peptide sequence: DFPITWPWK

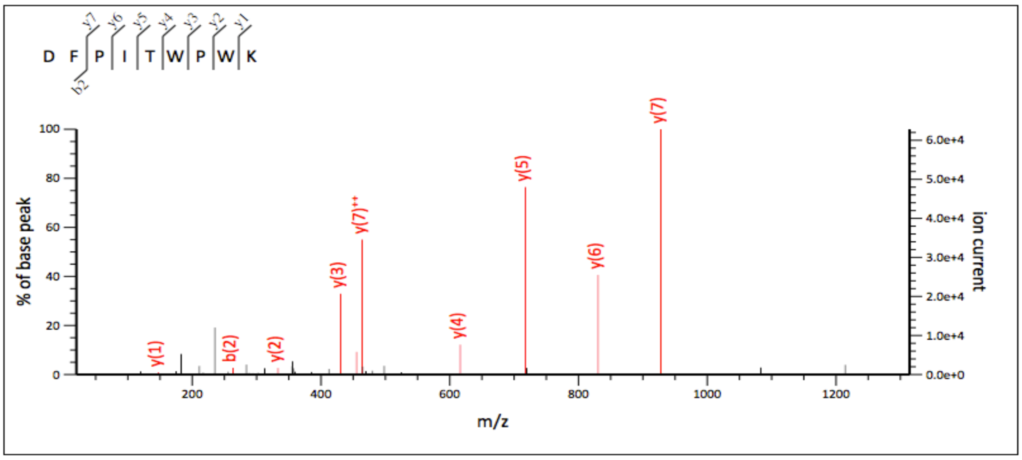

Peptide sequence: NECCQLLGQMPSECR

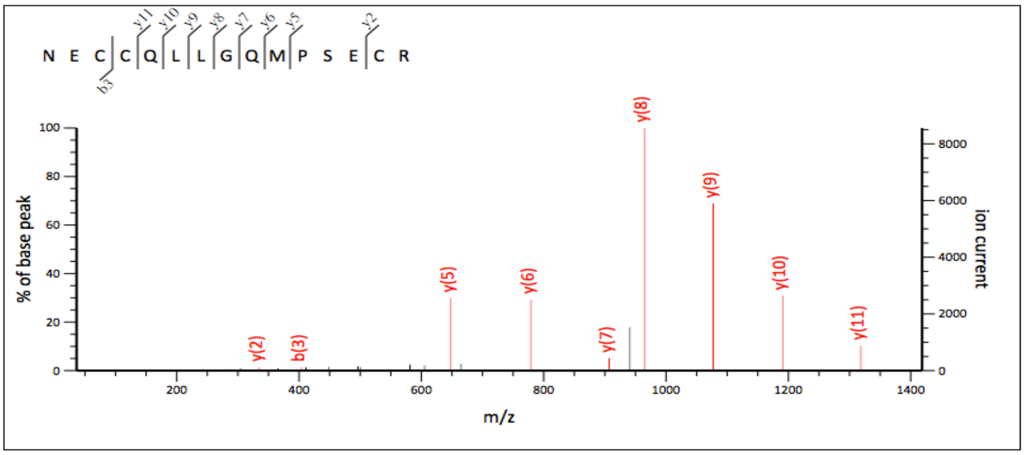

Spot 90, Protein name: 17.3 kDa class I heat shock protein; Protein ID: P02519

Peptide sequence: VDWKETPEAHVFK

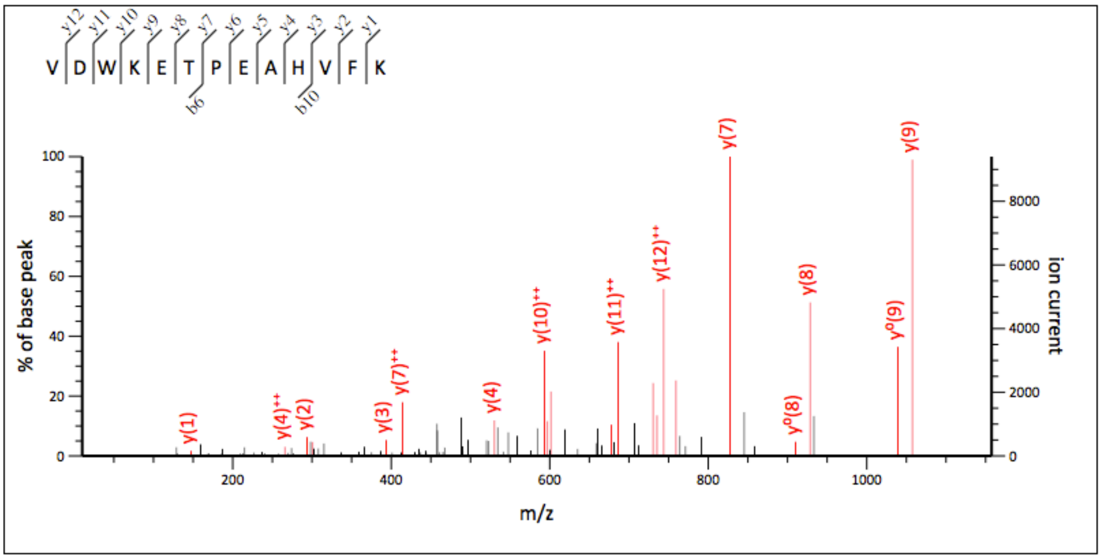

Peptide sequence: FRLPENAK

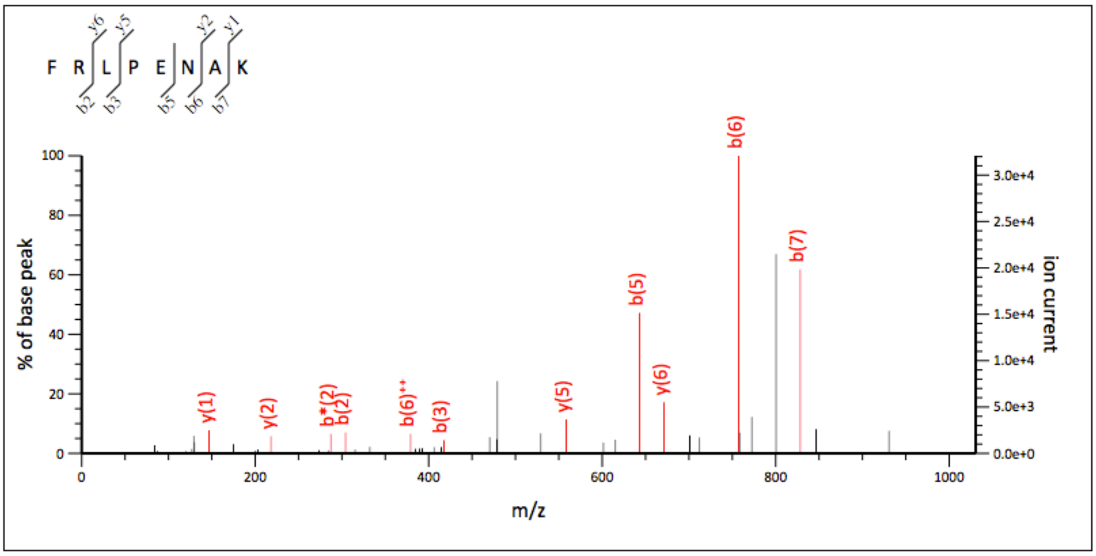

Peptide sequence: ASMENGVLTVTVPKEEIKKPDVK

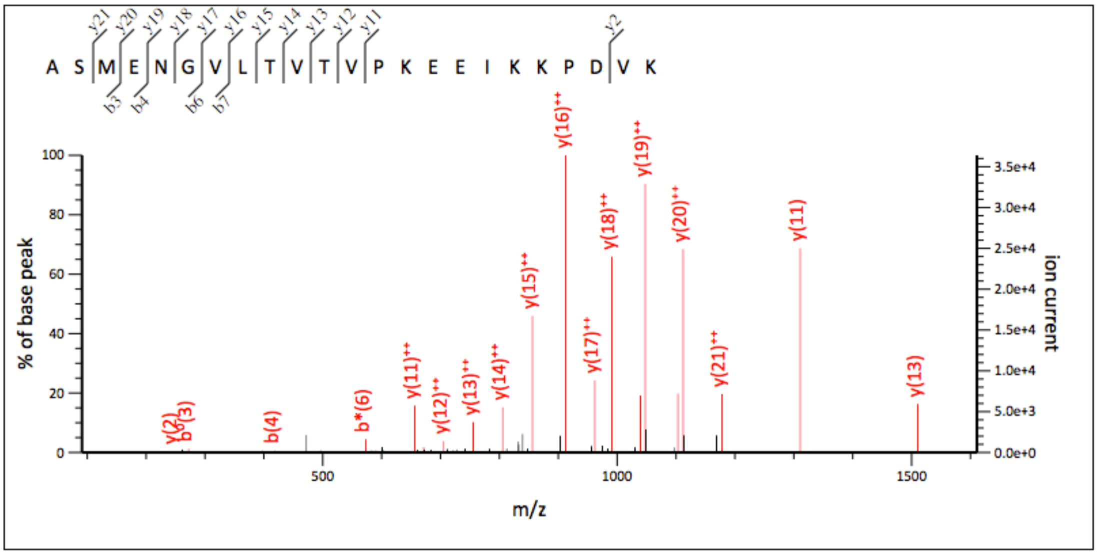

Spot 95 (Spot 78), Protein name: 18.3 kDa class I heat shock protein;   Protein ID: Q05832

Peptide sequence: ETPEAHVFKADLPGVK

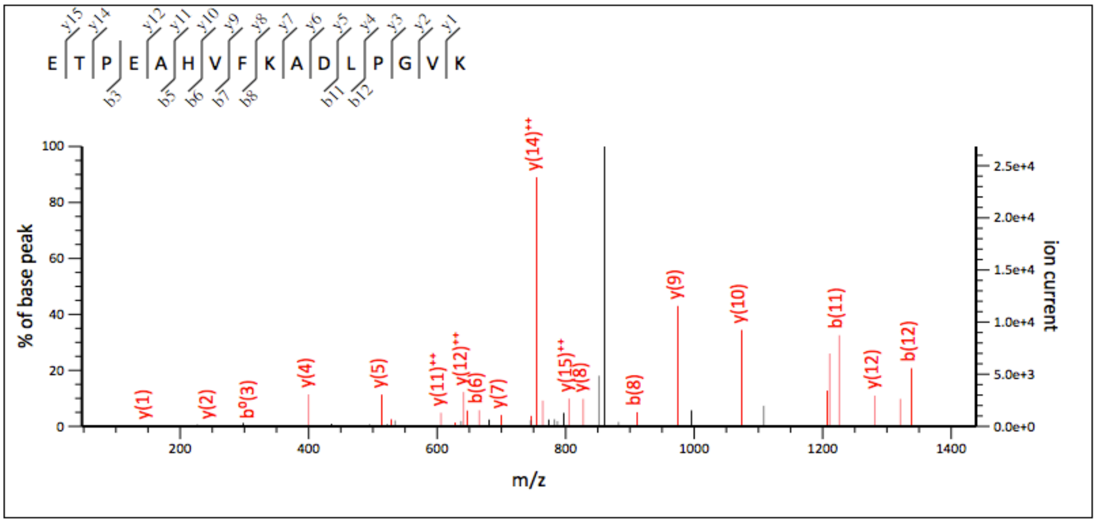

Peptide sequence: ADLPGVKKEEVK

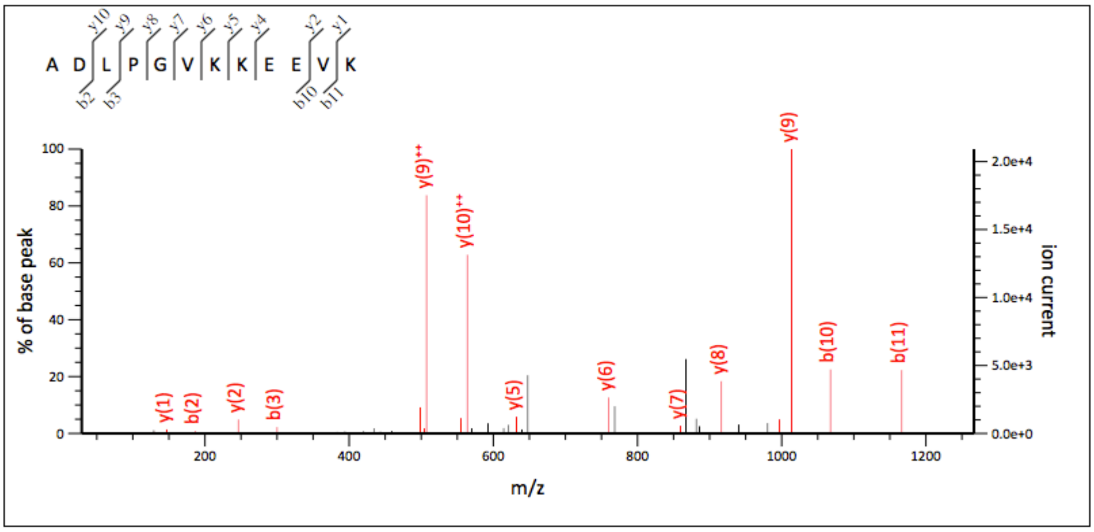

Spot 96, Protein name: 17.9 kDa class I heat shock protein； Protein ID: Q84Q77

Peptide sequence: IDWKETPEAHVFK

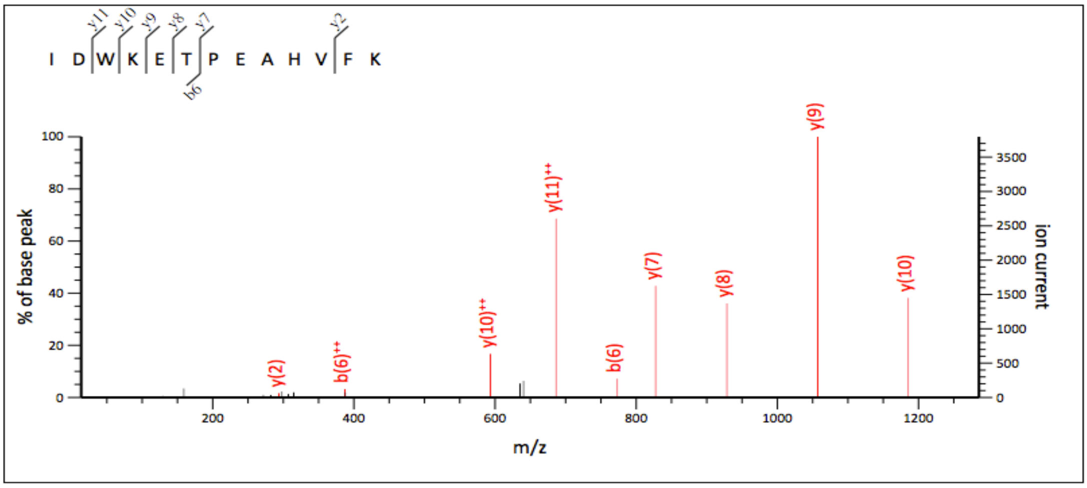

Peptide sequence: ASMENGVLTVTVPKEEAK

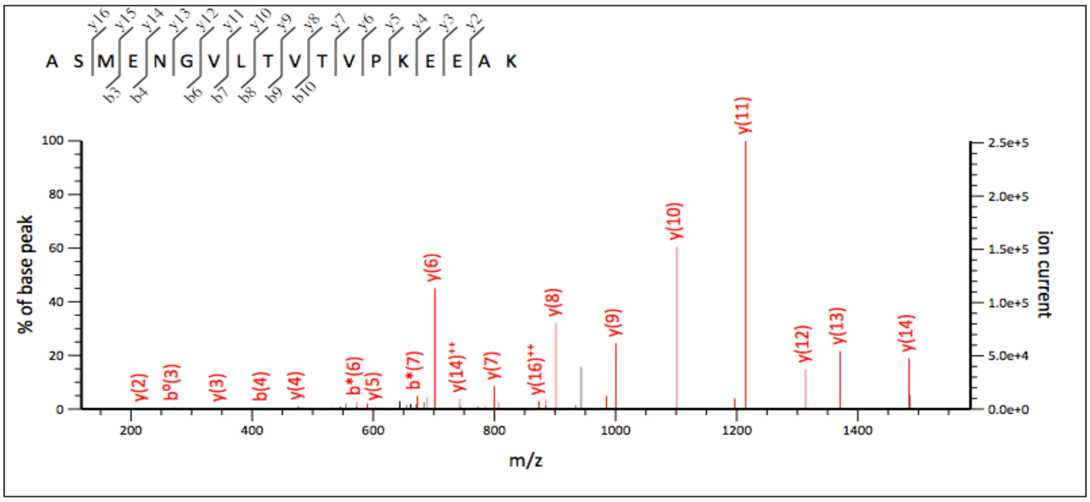

Spot 100, Protein name: 12S seed storage globulin 1； Protein ID: P12615

Peptide sequence: LQAFEPLR

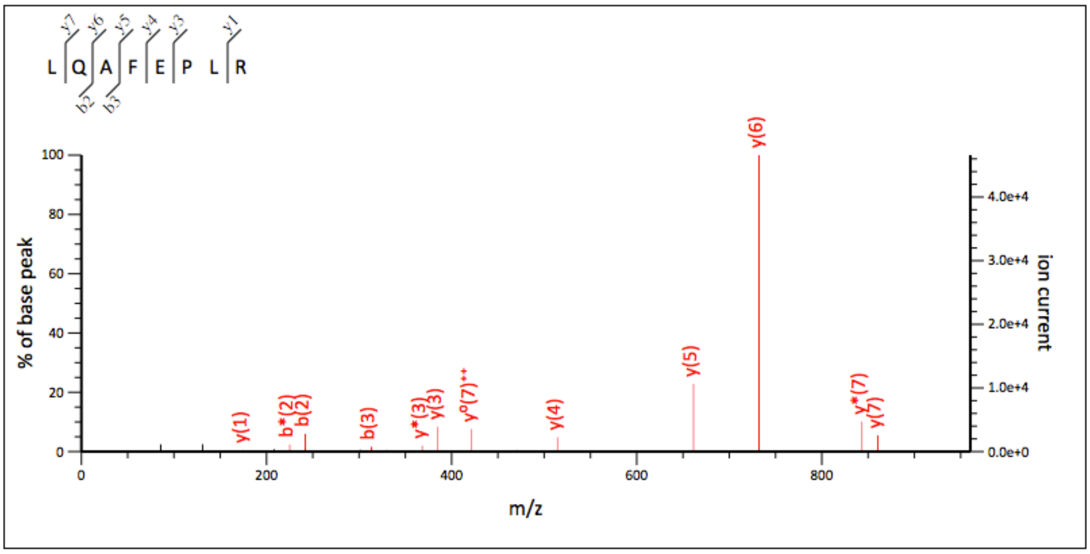

Peptide sequence: SQAGITEYFDEQNEQFR

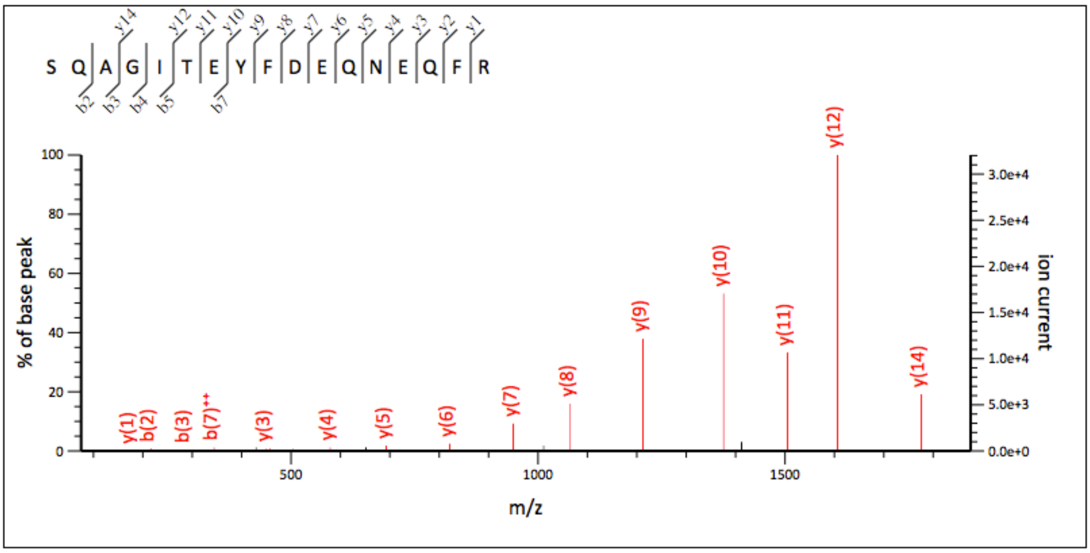

Peptide sequence: QKEFLLAGNNK

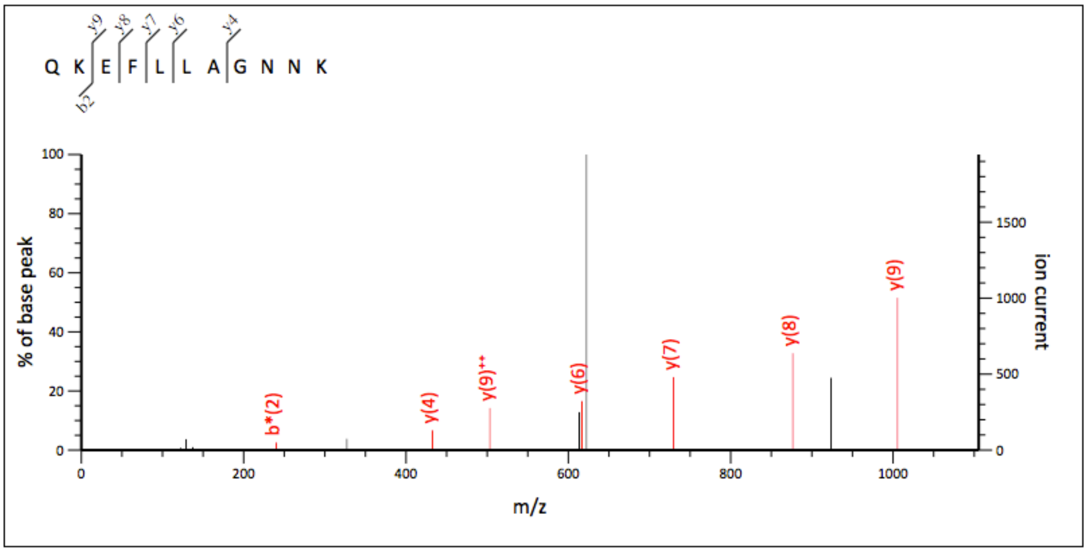

Peptide sequence: ALPVDVLANAYR

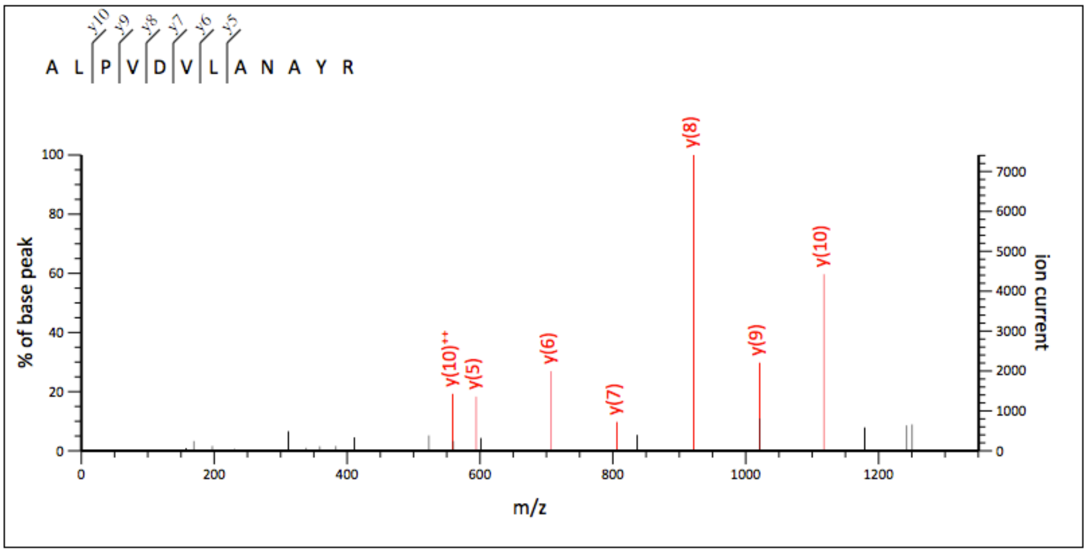

Spot 412 (Spot 362), Protein name: 12S seed storage globulin 2； Protein ID: P14812

Peptide sequence: LQAFEPLR

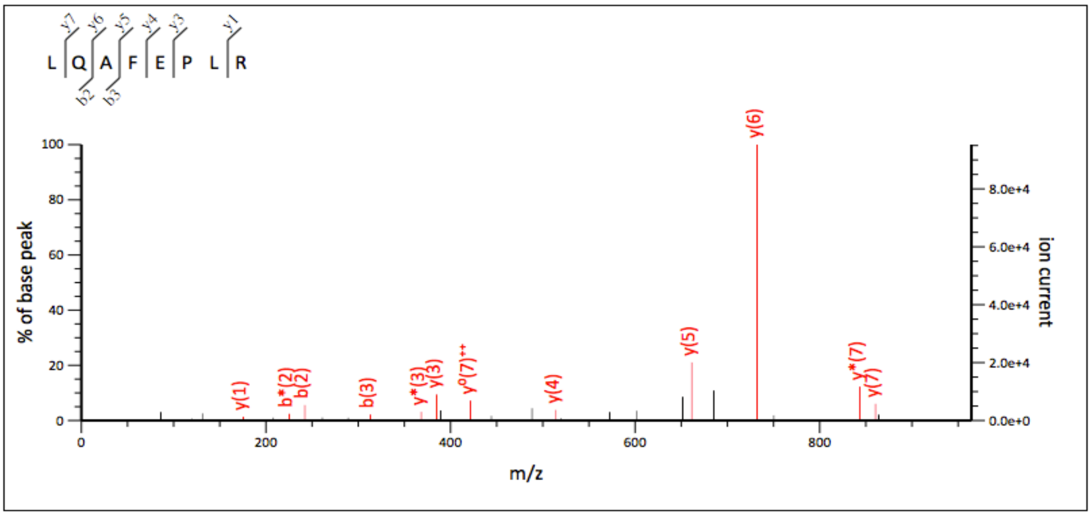

Peptide sequence: CAGVSVIR

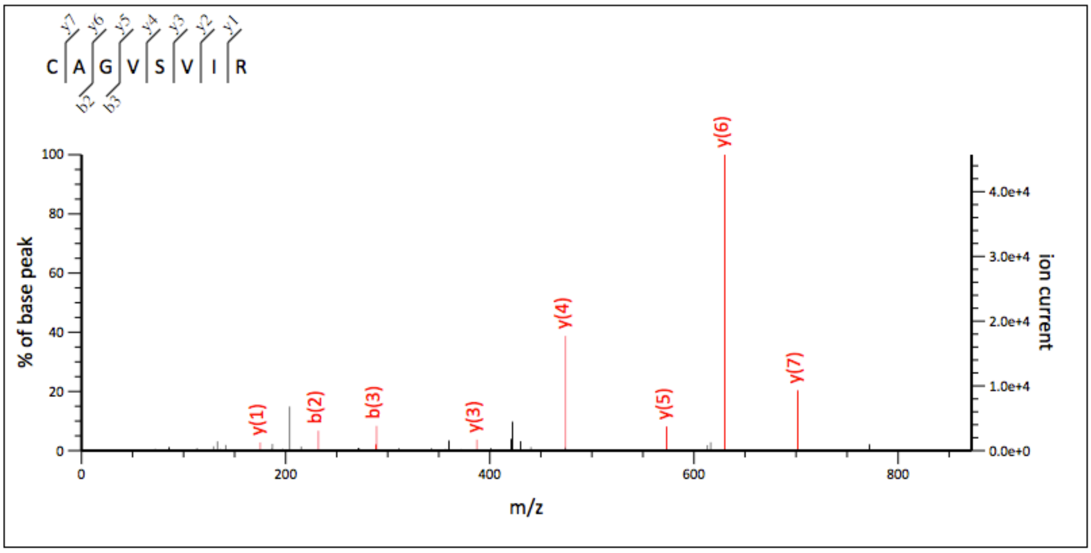

Peptide sequence: VIEPQGLLLPQYHNAPGLVYILQGR

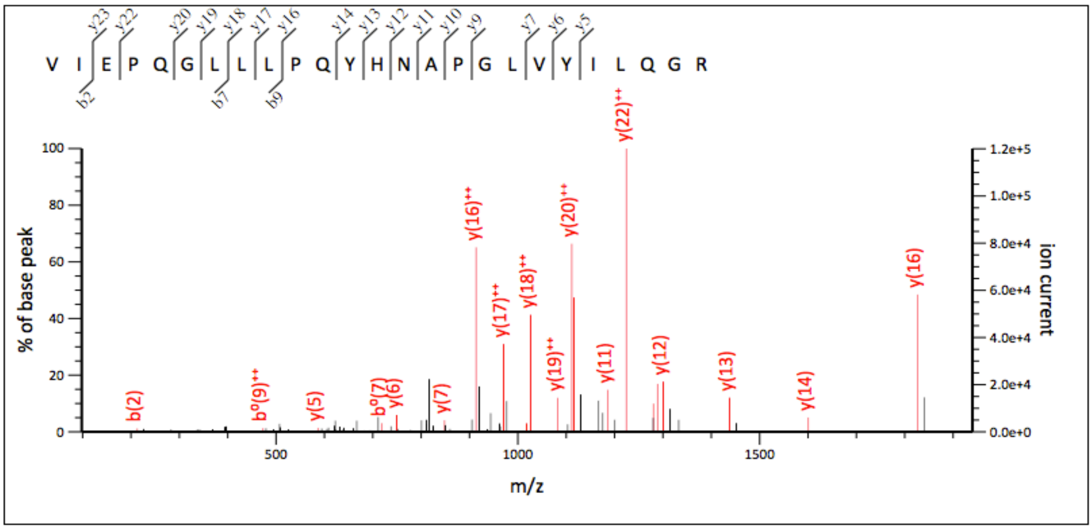

Peptide sequence: EFLLAGNNKR

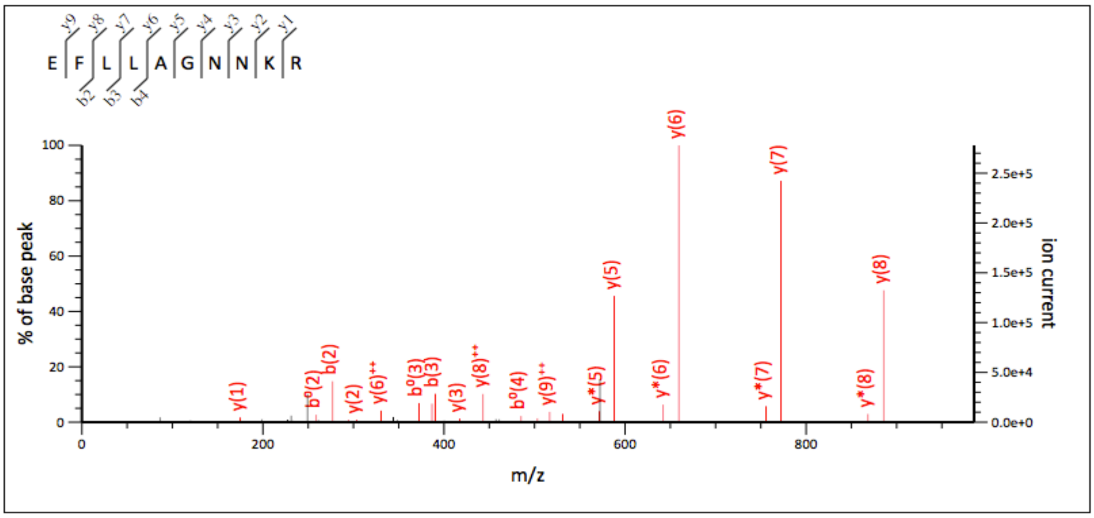

Peptide sequence: IQSQNDQRGEIIR

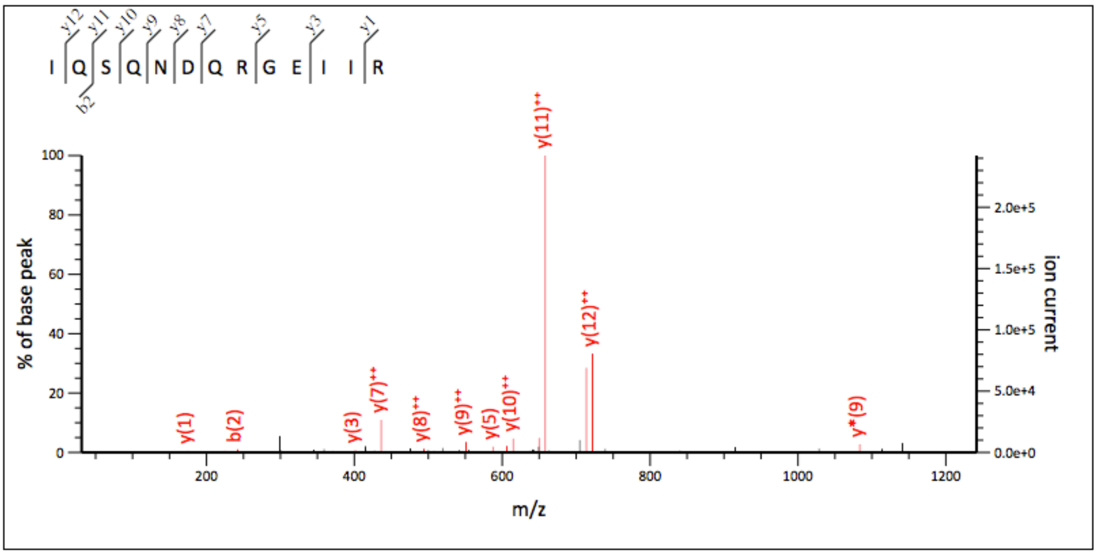

Peptide sequence: NFPTLNLVQMSATR

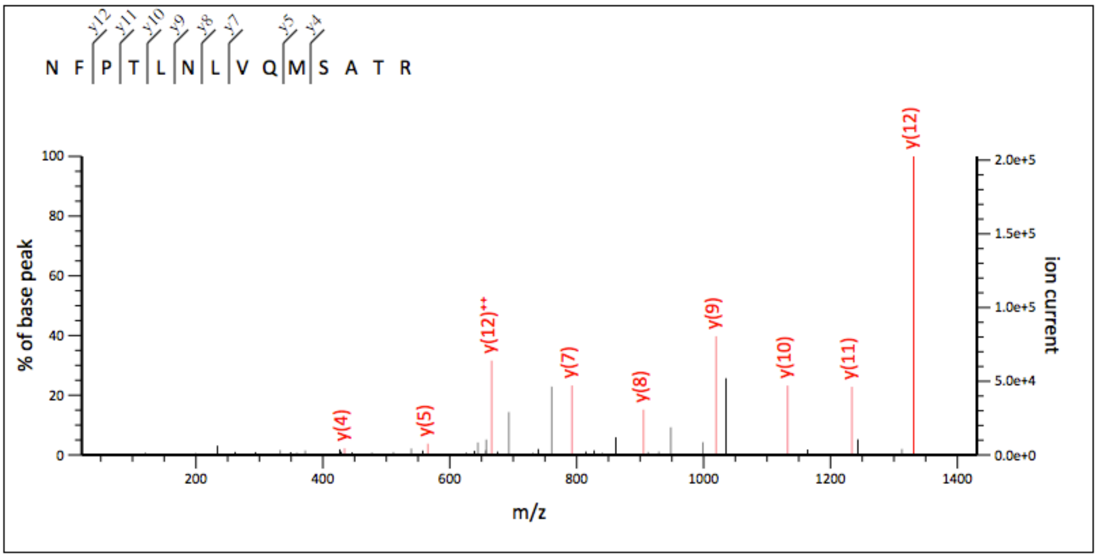

Peptide sequence: VQVVNNHGQTVFNDILR

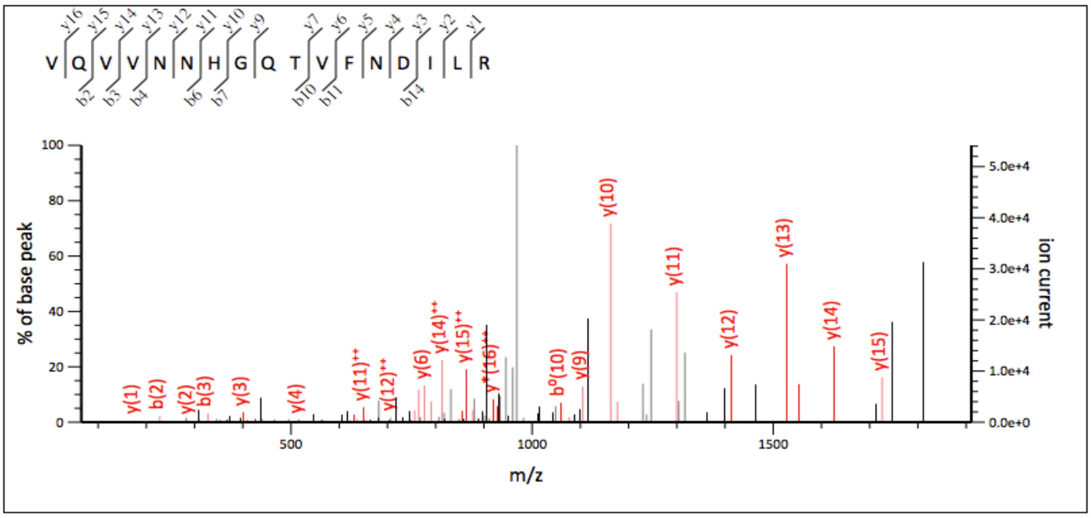

Peptide sequence: GQLLIIPQHYVVLK

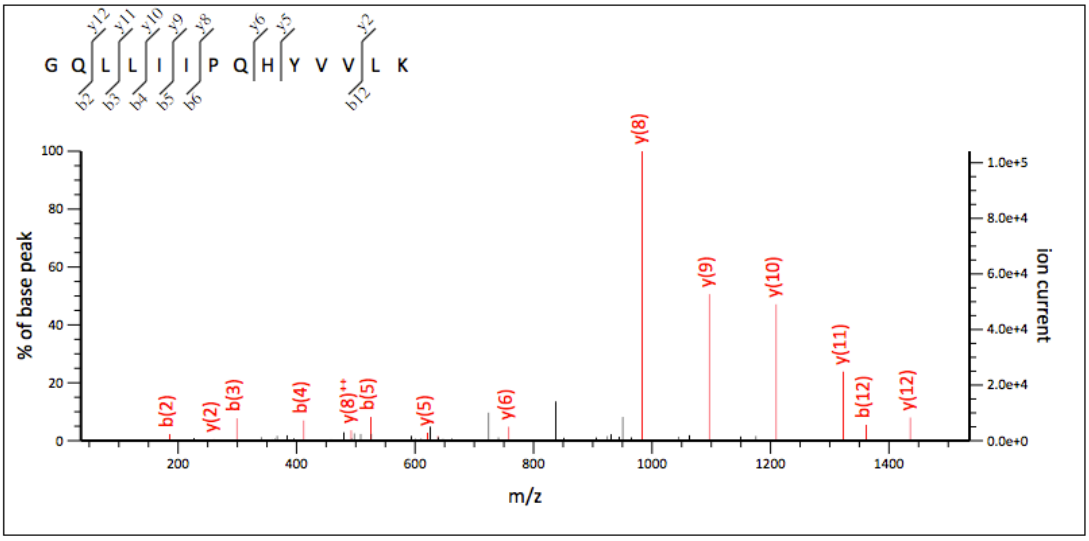

Peptide sequence: EGCQYISFK

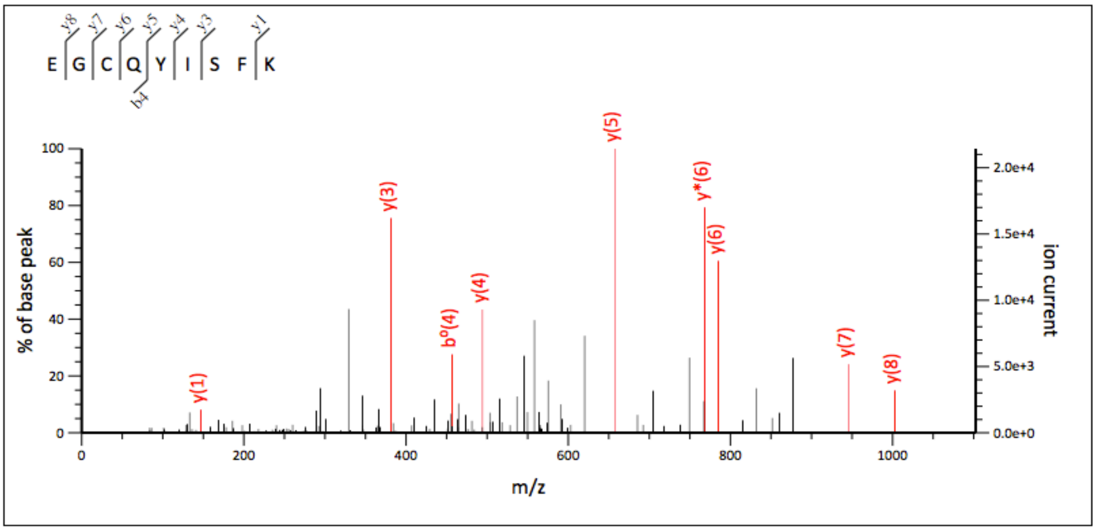

Peptide sequence: TNPNSMVSQIAGK

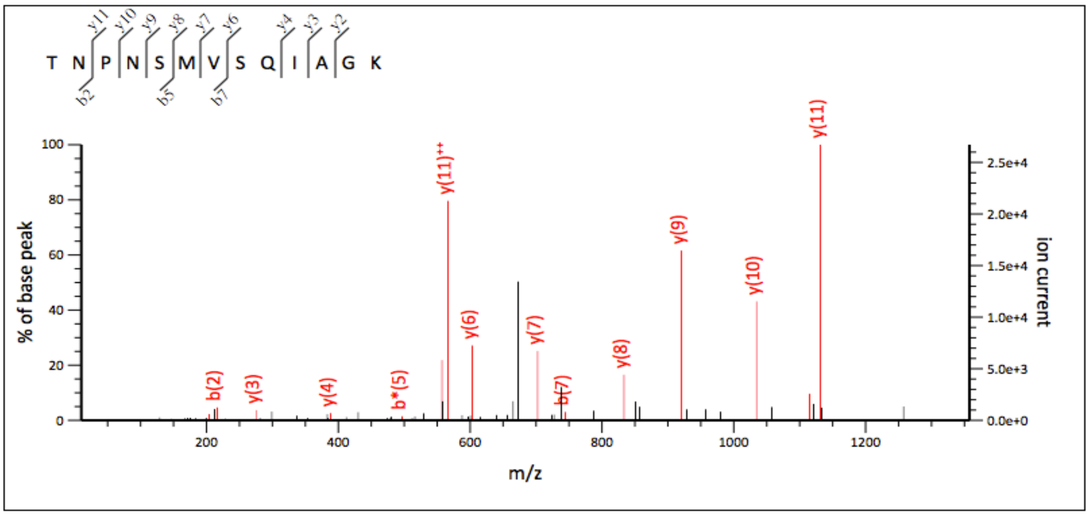

Peptide sequence: ALPVDVLANAYR

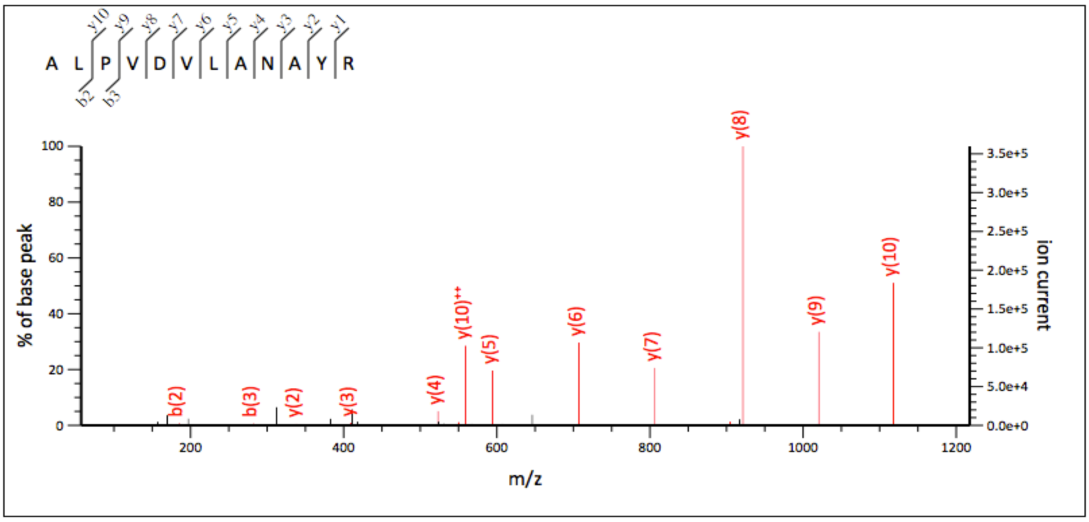

Peptide sequence: NNRGEEFDAFTPK

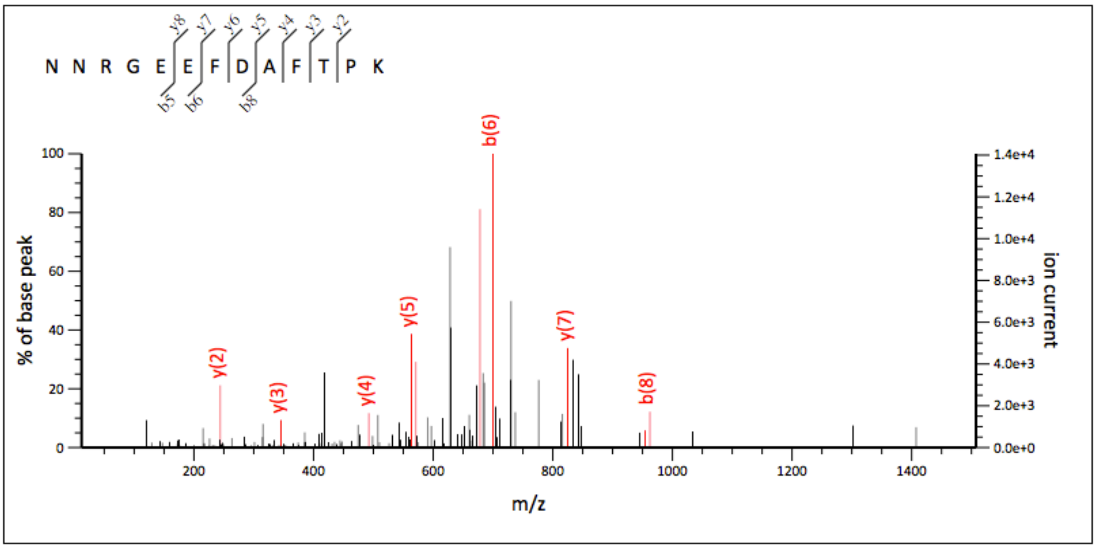

Spot 446, Protein name: Argininosuccinate synthase, chloroplastic； Protein ID: Q9SZX3
Peptide sequence: FELTFFSLNPELK

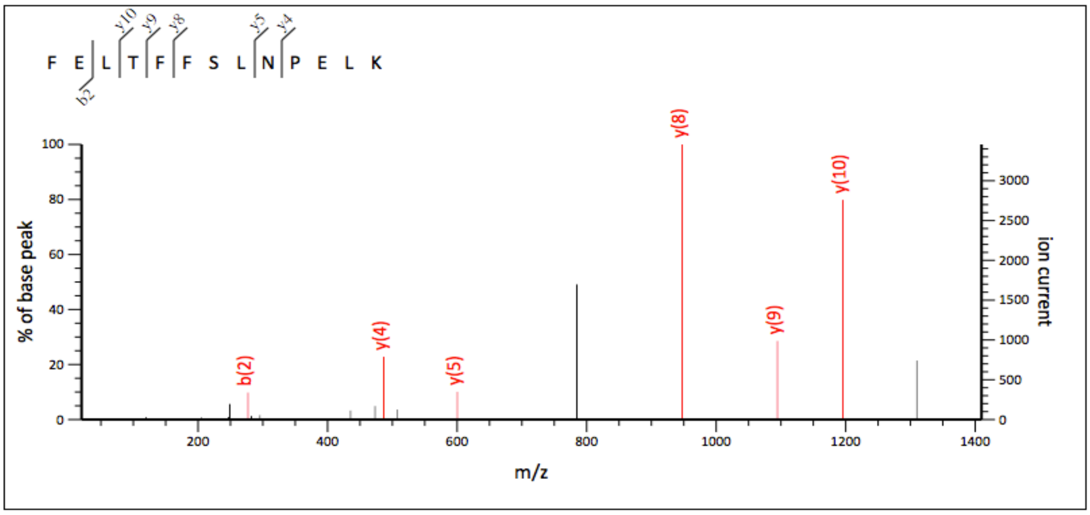

Peptide sequence: IDMVENR

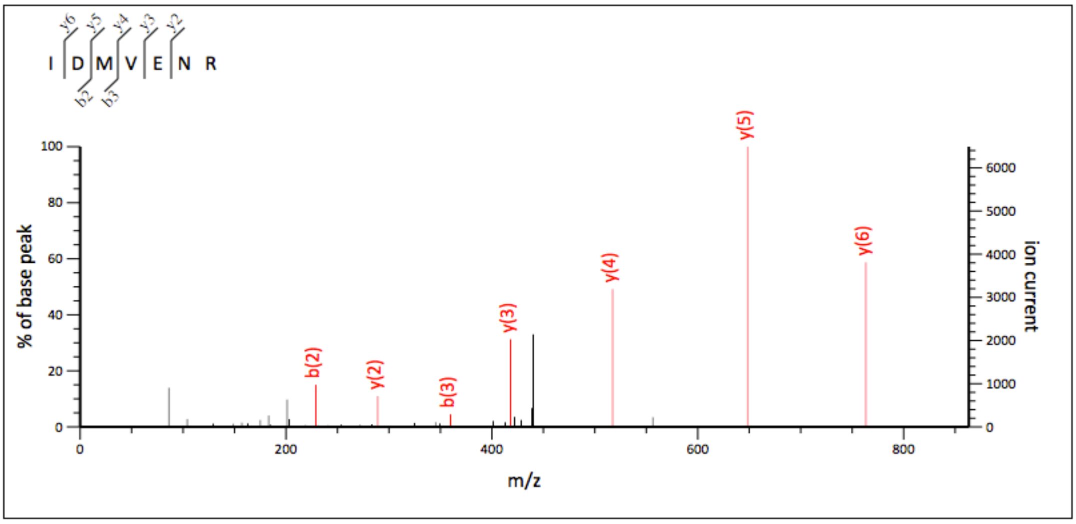

Spot 446, Protein name: ATP synthase subunit alpha, mitochondrial； Protein ID: P0C520
Peptide sequence: AAELTTLLESR

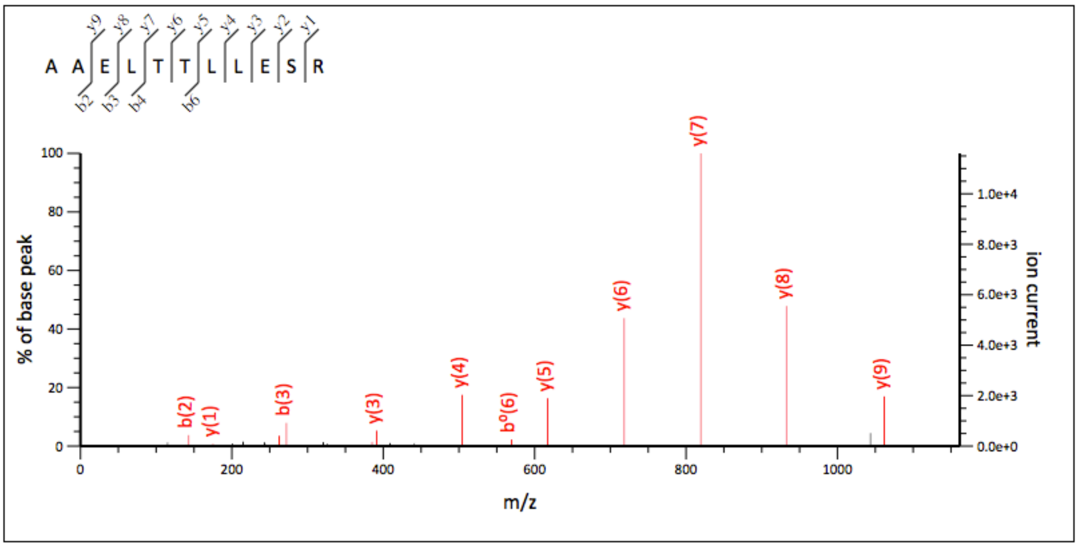

Peptide sequence: MTNFYTNFQVDEIGR

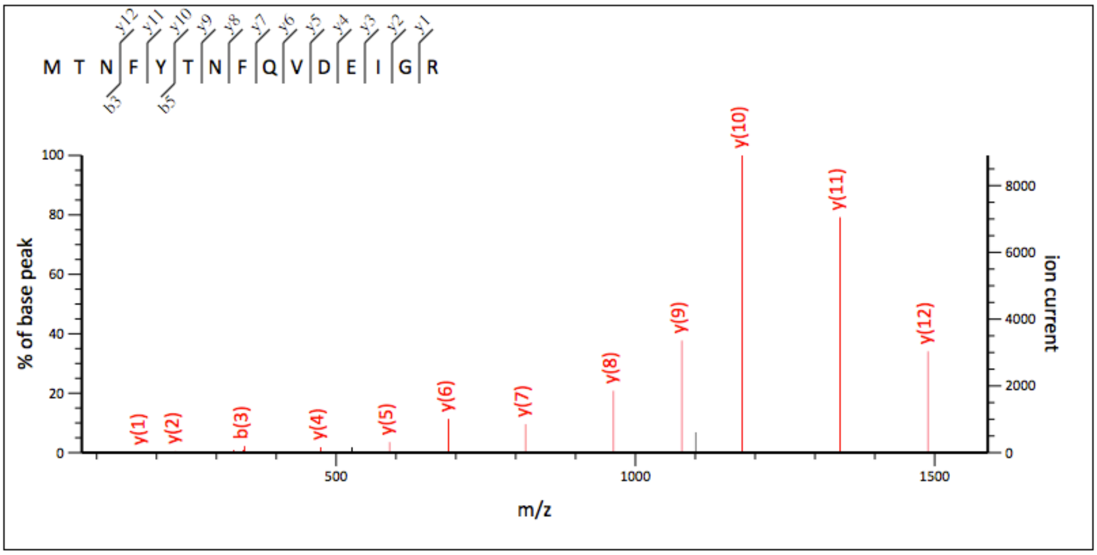

Peptide sequence: VVSVGDGIAR

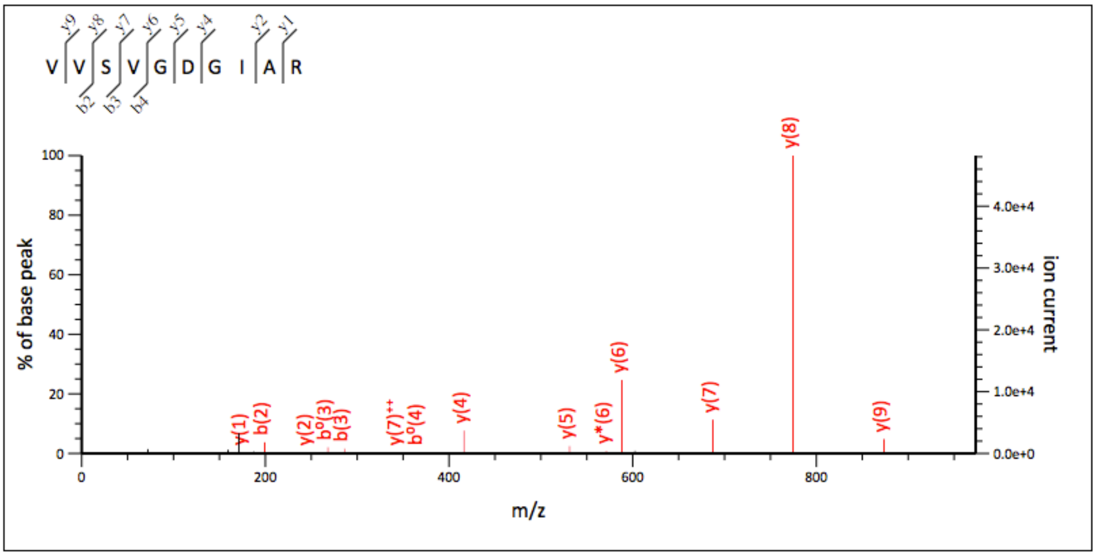

Peptide sequence: TGSIVDVPAGK

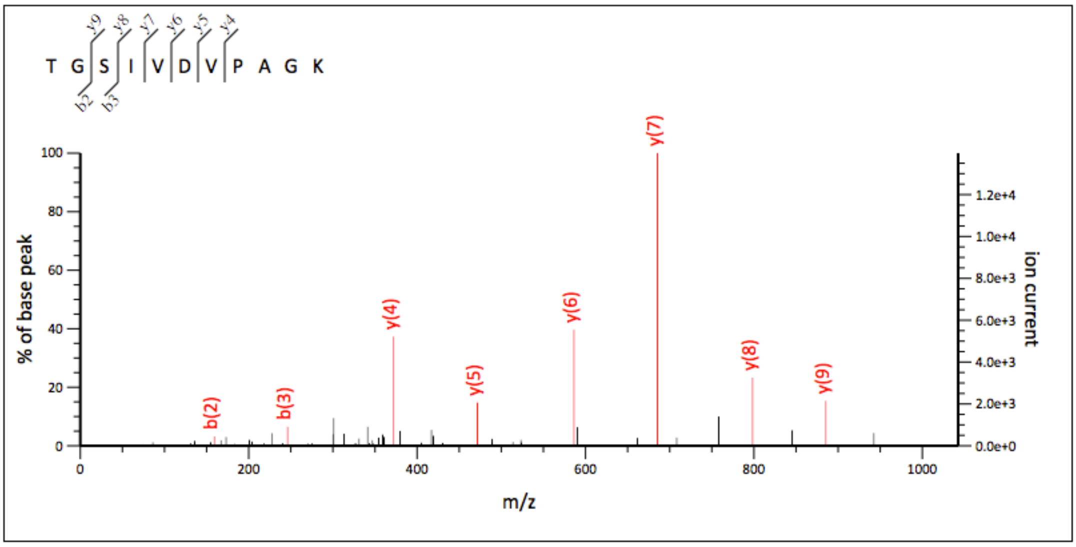

Peptide sequence: VVDALGVPIDGK

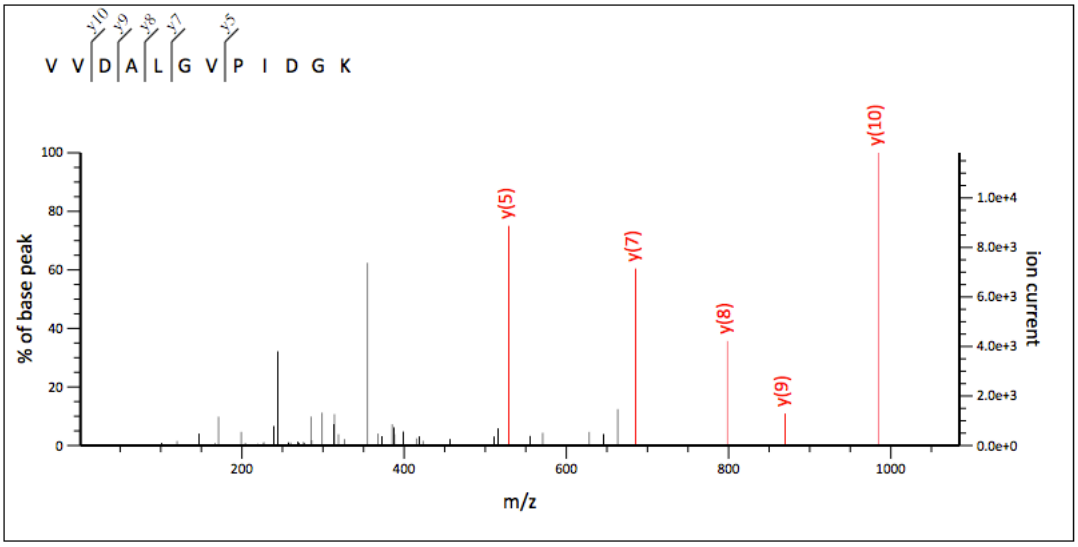

Peptide sequence: AVDSLVPIGR

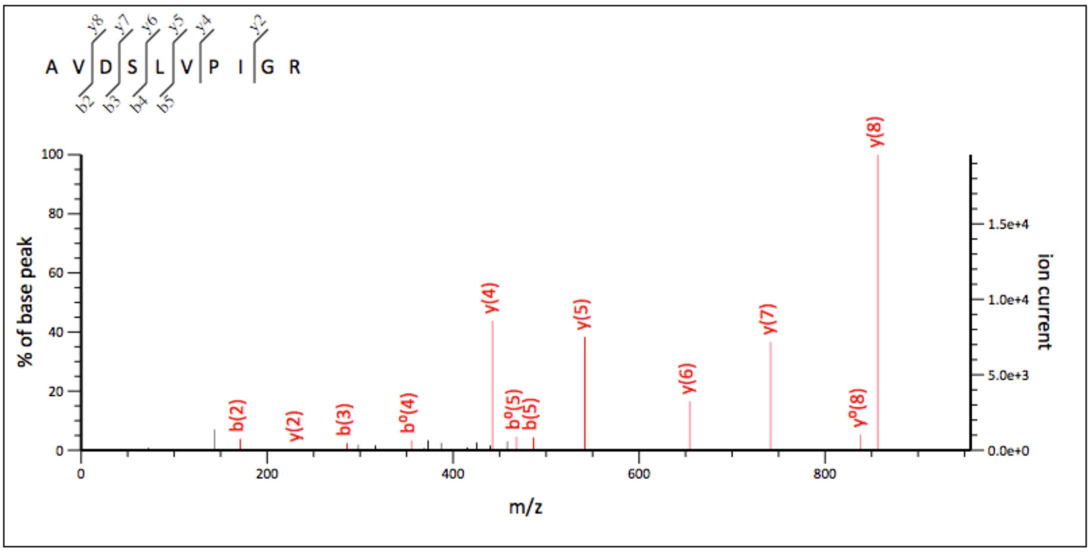

Peptide sequence: TAIAIDTILNQK

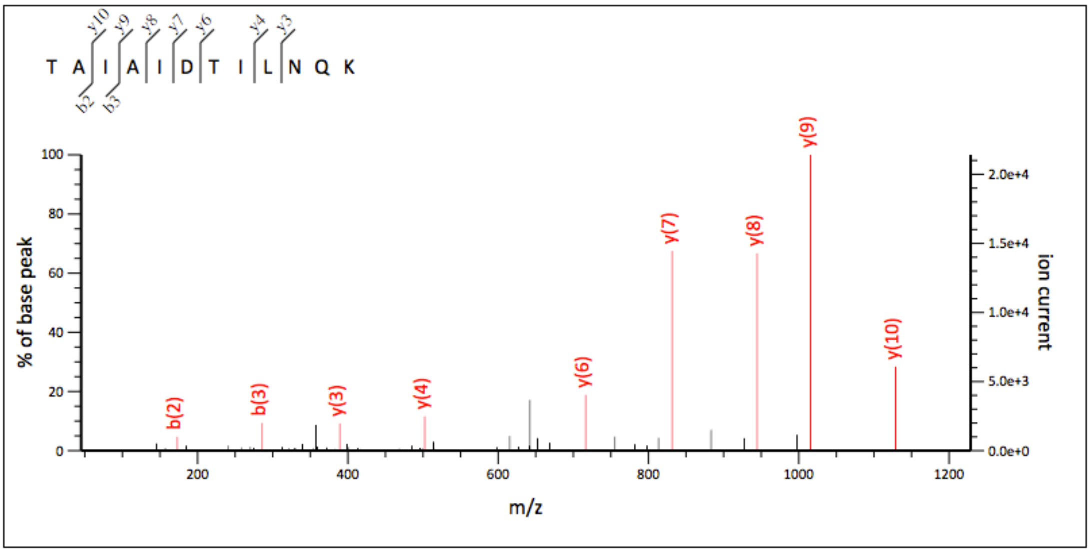

Supplement: Supplementary file 4 [file Image2.PDF]
